# Genetic control of meiosis surveillance mechanisms in mammals

**DOI:** 10.3389/fcell.2023.1127440

**Published:** 2023-02-23

**Authors:** Yan Huang, Ignasi Roig

**Affiliations:** ^1^ Genome Integrity and Instability Group, Institut de Biotecnologia i Biomedicina, Universitat Autònoma de Barcelona, Cerdanyola del Vallès, Spain; ^2^ Histology Unit, Department of Cell Biology, Physiology, and Immunology, Cytology, Universitat Autònoma de Barcelona, Cerdanyola del Vallès, Spain

**Keywords:** meiosis, checkpoint, infertility, human infertility, mammalian

## Abstract

Meiosis is a specialized cell division that generates haploid gametes and is critical for successful sexual reproduction. During the extended meiotic prophase I, homologous chromosomes progressively pair, synapse and desynapse. These chromosomal dynamics are tightly integrated with meiotic recombination (MR), during which programmed DNA double-strand breaks (DSBs) are formed and subsequently repaired. Consequently, parental chromosome arms reciprocally exchange, ultimately ensuring accurate homolog segregation and genetic diversity in the offspring. Surveillance mechanisms carefully monitor the MR and homologous chromosome synapsis during meiotic prophase I to avoid producing aberrant chromosomes and defective gametes. Errors in these critical processes would lead to aneuploidy and/or genetic instability. Studies of mutation in mouse models, coupled with advances in genomic technologies, lead us to more clearly understand how meiosis is controlled and how meiotic errors are linked to mammalian infertility. Here, we review the genetic regulations of these major meiotic events in mice and highlight our current understanding of their surveillance mechanisms. Furthermore, we summarize meiotic prophase genes, the mutations that activate the surveillance system leading to meiotic prophase arrest in mouse models, and their corresponding genetic variants identified in human infertile patients. Finally, we discuss their value for the diagnosis of causes of meiosis-based infertility in humans.

## 1 General aspects of mammalian gametogenesis

The perpetuation of most living beings and their genetic information across generations relies on a critical biological process-gametogenesis. In mammals, this process includes oogenesis and spermatogenesis, through which unipotent diploid precursor cells develop into mature haploid gametes, eggs in females, or sperm in males. After fertilization, the united egg and sperm form the embryo that develops into a new diploid organism carrying maternal and paternal genomic material.

During early mouse embryonic development, primordial germ cells (PGCs) are singled out at the epiblast (at ∼ embryonic day (E) 7.25) (Chiquoine, 1954; Ginsburg et al., 1990), migrate along the developing gut and eventually colonize the future gonads (at ∼ E10.5) (Molyneaux et al., 2001). Soon, PGCs switch from multipotential to bipotential and obtain the competence to initiate sexual differentiation and meiosis (Lesch and Page, 2012). At ∼ E12.5, the expression of the Y chromosome-encoded gene, Sry, determine the gonads to become the testes (Koopman et al., 1991). Consequently, PGCs commit to divergent development based on the cues from the somatic environment: female and male PGCs differentiate to their specialized gamete precursors: oogonia and spermatogonia, which initiate meiosis to form eggs through oogenesis or sperm through spermatogenesis (Edson et al., 2009).

In mammals, meiosis exhibits substantial sexual dimorphism (Handel and Eppig, 1997; Morelli and Cohen, 2005). Female meiosis is initiated roughly simultaneously in all oogonia during fetal development and subsequently arrests at the end of meiotic prophase I (dictyotene stage) around birth. It resumes producing eggs periodically after puberty over a defined reproductive lifetime. Female meiosis I does not complete until ovulation, and meiosis II only occurs under the trigger of fertilization, eventually generating only one haploid oocyte from one oogonium. In contrast, male meiosis is initiated in separate cohorts of spermatogonia after the onset of puberty and provides continuous sperm production throughout most of adult life. The two meiotic cell divisions in males are consecutive and result in four haploid sperm from each spermatogonium that initiates meiosis.

Both spermatogonia and oogonia enter meiosis during preleptotenema but before S phase. The sexually dimorphic timing of meiosis entry depends on the Stimulated by Retinoic Acid gene 8 gene (Stra8) (Handel and Schimenti, 2010). In females, retinoic acid (RA) synthesized in the mesonephric ducts (Bowles et al., 2006) induces Stra8 expression, resulting in meiosis initiation (Koubova et al., 2006; Baltus et al., 2006); however, in males, RA is degraded by CYP26B1 (gene cytochrome P450, family 26, subfamily b, polypeptide 1) from Sertoli cells, preventing the induction of Stra8 and thus blocking the meiotic entry (Bowles et al., 2006). The ability to enter meiosis is gained in males postnatally when the expression of CYP26B1 is repressed in male gonads (Koubova et al., 2006; Bowles et al., 2006; Anderson et al., 2008; Lesch and Page, 2012). The exact RA-Stra8 meiotic initiation pathway remains elusive. This is mainly due to the role of RA as a meiosis-inducing substance is unclear and has been challenged (Kumar et al., 2011; Vernet et al., 2020), particularly by a recent study showing that meiosis can normally occur in the absence of all RA receptors in female mice (Vernet et al., 2020). The role of STRA8 in meiotic initiation is more clear and STRA8 is suggested recently to trigger meiosis initiation in mice together with MEIOSIN in a broad transcriptional network, probably by activating genes responsible for suppressing the mitotic program and establishing a meiosis-specific chromosome structure under the presence of RA (Kojima et al., 2019; Ishiguro et al., 2020). Notably, other pathways are also suggested to mediate meiosis initiation in mice, such as the BMP-ZGLP1 pathway that works in parallel with RA-STRA8 signaling (Nagaoka et al., 2020), STRA8-independent RA-REC8 pathway (Koubova et al., 2014; Soh et al., 2015) and epigenetic regulated negative controls (Yamaguchi et al., 2012; Yokobayashi et al., 2013; Endoh et al., 2017).

### 1.1 Spermatogenesis

Mammalian male fertility requires millions of sperm produced daily by continuous spermatogenesis throughout reproductive life. The continual spermatogenesis is founded on a stem cell pool supplied by spermatogonial stem cells (SSCs) (de Rooij and Russell, 2000; Oatley and Brinster, 2008). Spermatogenesis continues with the mitotic expansion of spermatogonia, the meiotic divisions of spermatocytes, and the morphological transformations of spermatids.

SSCs are testis-specific stem cells derived from PGCs. In mice, male PGCs arrested at the G0/G1 phase migrate and differentiate into SSCs around 3 days *postpartum* (dpp) (Bellve et al., 1977; McLean et al., 2003). One subpopulation of these cells (Neurogenin 3 (NGN3)-negative) initiates the first round of spermatogenesis during the second week after birth; the other subpopulation develops into morphologically distinct, NGN3-positiveSSCs and supplies SSCs for spermatogenesis during adulthood (Yoshida et al., 2006). SSCs (As (A-single) spermatogonia) undergo symmetric division to produce SSCs for self-renewal or progenitor spermatogonia (Apr (A-paired) spermatogonia) for differentiation, which marks the beginning of spermatogenesis. SSC self-renewal predominates during the neonatal period to establish a stem cell pool (Shinohara et al., 2001) but only occurs periodically under steady-state conditions during adulthood to maintain the SCC pool (Oatley and Brinster, 2012). Apr spermatogonia undergo seven rounds of mitotic cell divisions to form undifferentiated Aal spermatogonia (Aal (A-aligned) spermatogonia)and differentiated A1, A2, A3, A4, In (Intermediate), and B spermatogonia. B spermatogonia differentiate into preleptotene spermatocytes *via* a final round of mitosis and initiate meiosis (Russell et al., 1993; de Rooij and Russell, 2000; Rato et al., 2012).

Diploid spermatocytes proceed through meiosis, resulting in haploid round spermatids. Subsequently, these round spermatids undergo structural and functional changes, including nuclear remodeling by chromatin condensation, removing the excess cytoplasm, and forming an acrosome and a sperm tail (spermiogenesis) (Hermo et al., 2010; Lehti and Sironen, 2016). As a result, spermatids become motile spermatozoa and are released to the central seminiferous lumen (spermiation). Spermatozoa will complete the final maturation to become fertilizable sperm in the epididymis.

Spermatogenesis occurs within the seminiferous tubules of the testis, in which germ cells in different stages of development are organized into a series of cell associations known as stages. In mouse testis, 12 stages have been defined (Hasegawa and Saga, 2012). RA pulses progressively stagger along the tubule and stimulate the spermatogonia to enter the rigidly timed pathway committed to meiosis. This determines the seminiferous epithelial cycle initiation and eventually enables the continuous release of spermatozoa (de Rooij and Russell, 2000).

In the seminiferous epithelium, Sertoli cells form specialized tight junctions (so-called “blood-testis barrier” (BTB)) at their base to separate the seminiferous epithelium into basal (where the spermatogonial population resides) and the adluminal compartments (where the meiotic and haploid germ cells reside). The BTB blocks the elements from the interstitial space to maintain homeostasis for meiotic and haploid germ cell development in the adluminal compartment (O’Donnell et al., 2000; Oatley and Brinster, 2008). The BTB remodels periodically (controlled by RA) to ensure preleptotene spermatocytes enter the adluminal compartment to initiate meiosis (Hasegawa and Saga, 2012). The steroidogenic Leydig cells reside in interstitial tissue between the seminiferous tubules and secrete testosterone under the influence of LH.

### 1.2 Oogenesis

Mammalian oogenesis begins during embryonic development and generates primary oocytes assembled in primordial follicles perinatally. The establishment of the pool of primordial follicles determines mammalian female fertility. Post-pubertally, primordial follicles are recruited irreversibly and develop into mature follicles during the estrous/menstrual cycle, eventually releasing mature and fertilizable oocytes. As a result, the ovarian reserve is gradually reduced, defining a finite female reproductive life span (Kerr et al., 2013; Li and Albertini, 2013; Wear et al., 2016; Hunter, 2017; Ruth et al., 2021).

In mice, after differentiation of PGCs, oogonia undergo mitotic divisions with incomplete cytokinesis, forming germ cell cysts in which daughter cells are connected by intercellular bridges (McLaren and Monk, 1981; Pepling and Spradling, 1998). On E13.5, oogonia in the cysts initiate meiosis and eventually differentiate into primary oocytes, which will complete the first meiotic prophase and arrest at dictyotene perinatally (Borum, 1961). After cyst breakdown, primary oocytes are enclosed in a layer of somatic pre-granulosa cells, forming primordial follicles by 4dpp (Pepling and Spradling, 2001). The formation of primordial follicles is a complex process. It requires the presence of germ cells (McLaren, 1984) and involves communication between oocytes and pre-granulosa cells (Pepling, 2012).

In mammals, massive oocyte culling accompanies oogenesis. Mouse oocyte numbers begin to decline since E14.5, remain about half at birth, and continue reducing postnatally. At 4dpp, eventually, only 20% of fetal oocytes remain in the ovaries (Malki et al., 2014; Hunter, 2017; Martínez-Marchal et al., 2020). This massive oocyte death might result from oocyte quality control (Hunter, 2017). Oocytes with potential defects due to the activation of LINE1 transposon are eliminated during embryonic development (E15.5-18.5) in mice, leaving only oocytes with limited LINE1 activity (Malki et al., 2014). Postnatally, oocyte culling occurs in response to errors in meiotic prophase I to remove oocytes that might have chromosomal defects (Di Giacomo et al., 2005). Additionally, the loss of oocytes is also suggested to be the self-sacrifice of the so-called nursing oocytes, similarly to a well-characterized process that occurs during oogenesis in *Drosophila* (Lei and Spradling, 2016). In *Drosophila,* during oogenesis, nurse cells surrounding the growing oocyte provide nutrients and other factors required for development. The nurse cells form a syncytium with the egg, where the cytoplasm and organelles are shared among the cells, allowing for efficient transport of substances to the growing egg. Additionally, the nurse cells also help to regulate the developmental program of the egg by providing signals and controlling the expression of specific genes. In this way, the nursing cells play a crucial role in ensuring the proper development and survival of the egg. Thus, the oocyte quality control processes select the most suitable oocytes for the next-generation.

Newly formed primordial follicles remain quiescent until recruited. A cohort of primordial follicles located at the anterior-dorsal region of the mouse ovary is activated to grow during the first week of postnatal development, the first wave of folliculogenesis (Cordeiro et al., 2015). After puberty, quiescent primordial follicles are continually recruited through primordial activation to initiate follicular development, forming primary follicles with a single layer of cuboidal granulosa cells (Lintern-Moore and Moore, 1979).

Primary follicles continue developing through two phases: pre-antral and antral phases. Through the pre-antral phase, primary follicles become secondary/pre-antral follicles with two or more layers of granulosa cells. This development is independent of gonadotropins and is mainly regulated by autocrine and paracrine signaling, specifically, the TGF-β family members such as oocyte-secreted GDF-9 and BMP-15 (Yan et al., 2001; Günesdogan and Surani, 2016; Namwanje and Brown, 2016). Through the antral phase, antral follicles are formed. The presence of an antrum-a granulosa cell-secreted fluid-filled cavity characterizes antral follicles. The follicle development during this phase depends on gonadotropins FSH and LH (Williams and Erickson, 2000). FSH stimulates granulosa cells to proliferate and secrete estrogens. LH stimulates the theca cells to produce progesterone and testosterone. More importantly, the rise of the FSH level during the menstrual cycle allows the selection of dominant follicles, enabling only some of the growing antral follicles to develop into ovulatory follicles (Zeleznik, 2004).

Since the initiation of follicular development, oocytes start to grow in size and are transcriptionally and translationally active (Lintern-Moore and Moore, 1979). However, they remain arrested at the end of the meiotic prophase, marked by a large nucleus-the germinal vesicle-with a prominent nucleolus. This arrest is maintained by the combined effects of the cyclic adenosine monophosphate (cAMP) and cyclic guanosine monophosphate (cGMP) (Norris et al., 2009; Jaffe and Egbert, 2017). When the follicles reach the preovulatory stage, in response to LH surge, oocytes will resume meiosis and complete maturation, as seen by the germinal vesicle breakdown. Subsequently, oocytes complete the first meiotic division but arrest at metaphase II upon ovulation and will resume meiosis if fertilized, eventually generating a mature oocyte with two or three polar bodies that will undergo apoptosis. Besides the nuclear maturation, which involves the haploidization of the genome, the oocyte cytoplasm must also mature through major translational, post-translational, and organellar modifications, which are essential for the completion of meiosis, fertilization, and early embryonic development (reviewed in Li and Albertini, 2013).

## 2 Meiosis

Meiosis is a specialized cell division critical for gametogenesis in all sexually reproducing organisms. Through meiosis, a diploid parental cell gives rise to haploid daughter cells, and this is achieved by a single round of DNA replication followed by two rounds of cell divisions (Kleckner, 1996). Homologous chromosomes separate during the first division (meiosis I), and sister chromatids separate during the second division (meiosis II) analogously to mitosis, resulting in the generation of haploid cells. A canonical meiotic program present in most organisms (e.g., mammals, budding yeast, plants, *etc.*) will be briefly described in this section and expanded in detail, focusing on the two major meiotic events, synapsis and meiotic recombination (MR), in the following sections. Findings in mice will be prioritized to discuss in line with the scope of this review. However, data from other species, particularly yeast, will be addressed whenever necessary or of interest.

**FIGURE 1:**
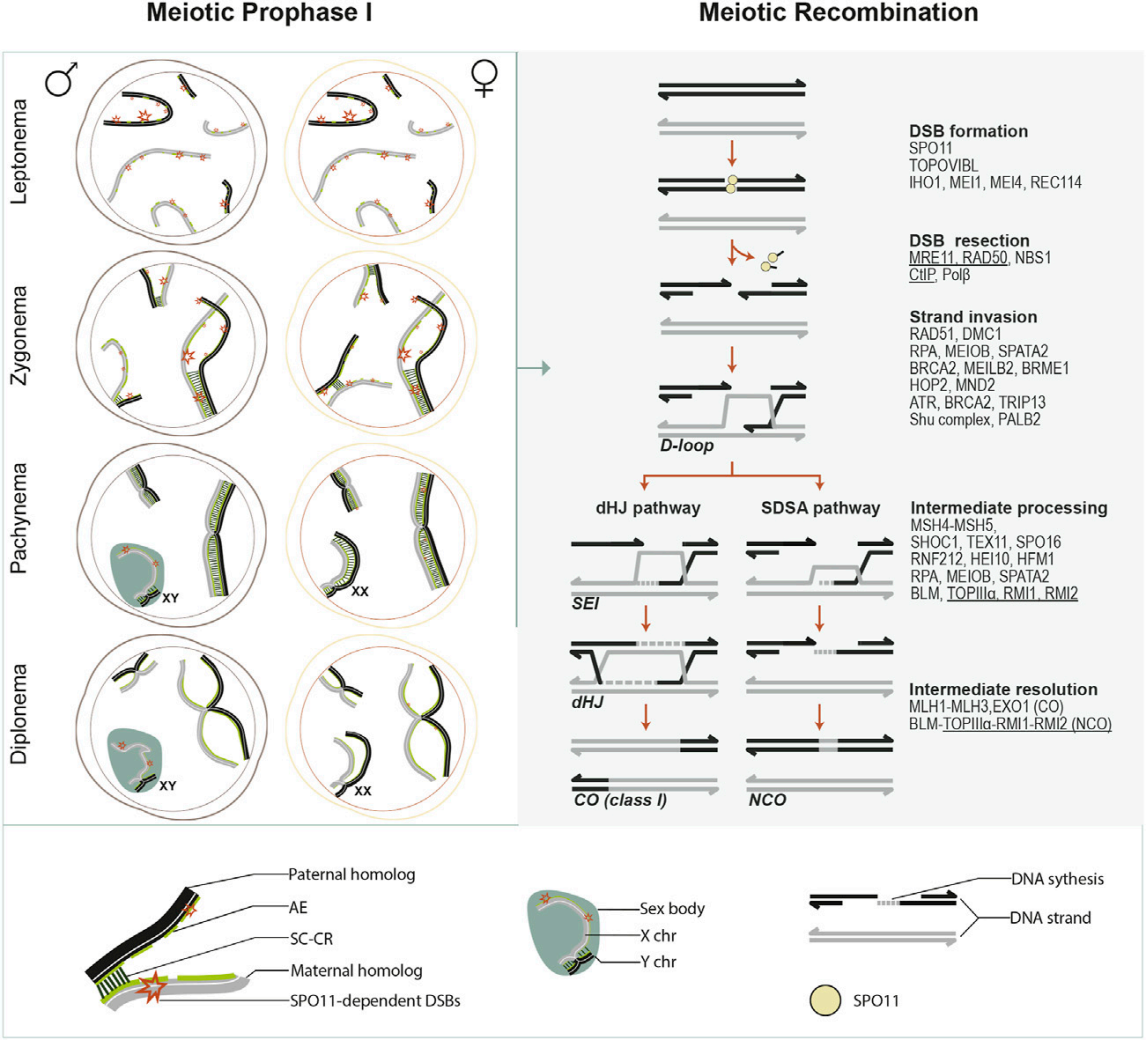
Meiotic prophase I overview. This schematic illustrates chromosome dynamics during meiotic prophase I in spermatocytes and oocytes (left panel) and the meiotic recombination pathway (right panel). Left panel, in meiotic prophase I, each paternal (black) or maternal (grey) homologous chromosome is organized around a chromosomal axis. During leptonema, axial elements (AEs) develop for each chromosome, and programmed SPO11-induced DSBs are generated as recombination initiates. During zygonema, synapsis initiates between paired homologs. Then it spreads along with the entire AEs as the SC central region (CR) proteins (consisting of the transverse filaments (TFs) and the central element (CE)) are installed between the AE. AEs are then designated as lateral elements (LEs) of the SC. In the meantime, DSBs are gradually repaired as recombination progresses. By pachynema, homologous chromosomes are fully synapsed, except for the heteromorphic X and Y chromosomes in the spermatocytes, which synapse only in a short pseudoautosomal region and form a transcriptionally silent chromatin compartment known as the sex body. By the end of pachynema in spermatocytes or during late pachynema/early diplonema in oocytes, meiotic recombination completes as DSBs on autosomes are all repaired, and crossovers (COs) are generated. During diplonema, the CR is disassembled, and homologous chromosomes are only held together at the CO sites (chiasmata). From diplonema, spermatocytes progress to metaphase I, completing meiotic divisions without interruption. In contrast, oocytes arrest at the dictyate stage until meiotic resumption after puberty. Right panel, several major events and critical transitions occur during meiotic recombination. Mammalian proteins that are, or are predicted (underlined) to be, involved in each event are listed. SPO11 catalyzes DSB formation in association with its accessory proteins. DSB ends are further resected through a series of nucleolytic activities mediated by the MRN complex (MRE11- RAD50-NBS1) and others. As a result, a short oligonucleotide covalently attached to SPO11 (SPO11 oligo) is released, and 3′ ssDNA tails are generated, which are immediately coated by ssDNA binding proteins (such as RPA, MEIOB, SPATA22, *etc.*). Recombinases DMC1 and RAD51 assemble at resected 3’ ssDNA tails, promoted by recombination proteins such as MEILB2, BRCA2, BRME1, *etc.* RAD51 and DMC1 coated ssDNA are stabilized by HOP2-MND1 and engage in homology search and strand exchange, resulting in D-loop formation. The repair can proceed by either a double Holliday junction (dHJ) pathway or synthesis-dependent strand annealing (SDSA). ZMM proteins and other factors control this by processing and stabilizing the recombination intermediates. In the dHJ pathway, D-loops are further stabilized by MutSγ homologs (MSH4 and MSH5), and the second end of the DSB is captured to form a dHJ, requiring RPA-MEIOB-SPTATA22 complex. ZMM proteins such as HEI10 and RNF212 facilitate the recruitment of mismatch repair factors MutLβ homologs (MLH1, MLH3). MutLβ and EXO1 mediate the resolution of dHJ, primarily giving rise to crossover (CO) products. In SDSA, the invading strand is displaced after DNA synthesis and reanneals to the other end of the DSB, followed by further DNA synthesis and nick ligation, ultimately giving rise to non-crossover (NCO) products.

Meiosis is characterized by an extended prophase I, during which MR occurs (Figure 1). MR initiates in early prophase I with the formation of numerous DNA double-strand breaks (DSBs) catalyzed by the conserved SPO11 protein (Keeney, 2001). DSB ends undergo resection and generate 3′ssDNA ends, subsequently bound by the RecA family of strand exchange proteins (DMC1, RAD51) (San Filippo et al., 2008). This protein nucleofilament searches and invades homologous repair templates, initiating the repair pathways to form crossovers (COs), with reciprocal exchange of chromosome arms flanking the DSB site, or non-crossovers (NCOs), with no exchange of flanking parental sequences (Keeney and Neale, 2006; Hunter, 2015). NCOs promote homolog pairing while COs establish the connections between homologous chromosomes to ensure accurate segregation at meiosis I and reshuffle parental alleles to increase genetic diversity in offspring (Hunter, 2015; Lam and Keeney, 2015).

MR is tightly integrated with a highly-organized and dynamic chromosome structure throughout the five substages of meiotic prophase I (leptonema, zygonema, pachynema, diplonema, and diakinesis) (Zickler and Kleckner, 1999). During leptonema, the chromatin condenses at the developing chromosomal axes, and recombination initiates. The axes provide a rod-like center for the loops of every pair of chromatids to anchor, defining a loop-axis structure essential for DSB formation and repair template choice (Subramanian and Hochwagen, 2014). During zygonema, maternal and paternal homologs progressively pair. The loop-axis organization makes this close alignment of homolog axes possible. However, understanding how base-pair resolution pairing is achieved in the context of the complex meiotic chromosome architecture is limited. Several regulation layers, including meiotic recombination and dynamic chromosome movement, are suggested to promote homolog pairing (Bolcun-Filas and Handel, 2018). Synapsis initiates as a tripartite proteinaceous scaffold–the synaptonemal complex (SC)- which starts to form between the paired homologous chromosome axes to create an intimate association between them. While in some organisms (e.g., *Neurospora*, and *Coprinus*), synapsis initiates only after all homologs complete pairing, in budding yeast and mammals, synapsis begins concomitantly with homolog pairing at zygonema. The telomeres and several interstitial sites of DSB-mediated inter-homolog associations are often where synapsis initiates (Fung et al., 2004), while in mice and several organisms with metacentric chromosomes, including humans, centromeres are often the last to synapse (Roig et al., 2010; Bisig et al., 2012; Qiao et al., 2012). Once it initiates, synapsis quickly spreads along the chromosomes in both directions in a zipper-like manner. At pachynema, the SC is fully installed along the entire length of all homologous chromosomes (Fraune et al., 2012). The last recombination steps after strand invasion occur in the SC context, which further helps keep the homologs in association, generating COs at the end of pachynema. Subsequently, the SC disassembles asymmetrically between homologs throughout diplonema and diakinesis, accompanied by changes in chromosome compaction (Gao and Colaiácovo, 2018). By late diakinesis, the highly condensed bivalents only remain connected by chiasmata, the cytological manifestation of COs. These inter-homologous connections ensure correct segregation under tension by allowing homolog pairs to stably bi-orient at the metaphase I spindle (Handel and Schimenti, 2010). As Meiosis I completes, maternal and paternal chromosomes are separated into daughter cells. Then in Meiosis II, sister chromatids separate, ensured by their centromeric cohesion, resulting in the generation of haploid cells (Ishiguro, 2019).

Meiosis must be carefully monitored to preserve the order of meiotic events and avoid producing aberrant chromosomes and defective gametes (Subramanian and Hochwagen, 2014). In mice, surveillance mechanisms monitor recombination and synapsis at the pachytene stage (meiotic checkpoint) (Roeder, 2000) and control bipolar attachment to the spindle at metaphase I (the spindle assembly checkpoint, SAC) (Touati and Wassmann, 2016). Recent mice findings have revealed new mechanistic insights on how meiotic checkpoints monitor these meiotic prophase events in mammals, which will be mainly discussed below. The roles of the meiotic checkpoint machinery in preserving the order of chromosomal events during the meiotic prophase I will also be presented in the following sections whenever necessary.

### 2.1 The synaptonemal complex and synapsis

The SC is a highly conserved meiosis-specific feature. This is likely attributed to a conserved SC organization, e.g., the coiled-coil domains (Gao and Colaiácovo, 2018), whereas its component proteins share little similarity at the amino acid sequence level (Grishaeva and Bogdanov, 2014; Fraune et al., 2016). The SC serves as the scaffold for the close juxtaposition of homologous chromosomes and is intimately associated with chromosome pairing, synapsis, and recombination (Fraune et al., 2012; Cahoon and Hawley, 2016; Geisinger and Benavente, 2017; Gao and Colaiácovo, 2018). Fully formed SC is revealed as a tripartite structure by electron microscopy, consisting of two LEs that run along the electron-dense chromatin and flank a CR (Moses, 1969), composed of a central element (CE) and numerous transverse filaments (TFs). In mammals, eight meiotic-specific SC proteins have been identified and characterized so far (Schücker et al., 2018): SYCP2 and SYCP3 as the LE proteins (Lammers et al., 1994; Offenberg et al., 1998); SYCP1 as the TF protein (Meuwissen et al., 1992), and SYCE1, SYCE2, SYCE3, TEX12, and SIX6OS1 as the CE proteins (Costa et al., 2005; Hamer et al., 2006; Schramm et al., 2011; Gómez-H et al., 2016).

The SC plays a universal role, as providing order within the nucleus during prophase, in all species. But it may also have diverse roles in many organisms. Notably, it is essential for multiple steps during MR (Zickler and Kleckner, 2015). The SC regulates programmed DSB formation as synapsis shuts off the SPO11 activity (Kauppi et al., 2013). The AE proteins are closely associated with the development of recombination protein complexes. The CR plays a significant structural role in these complexes’ assembly, maintenance, and turnover, thereby enabling the maturation of the DSBs into COs subject to interference. In mice, recombination can not be completed without the CR proteins (Bolcun-Filas et al., 2007; Schramm et al., 2011; Fraune et al., 2012; Gómez-H et al., 2016). Moreover, the SC is responsible for holding homologs after the repair of NCO-fated DSBs and maintaining interhomolog interactions until COs are formed (Zickler and Kleckner, 1999; Qiao et al., 2012). Finally, the SC might be centrally important in the surveillance of meiotic recombination and HORMAD-regulated monitoring of synapsis.

The SC undergoes a dynamic cycle through its assembly, a highly dynamic steady-state, and disassembly (Gao and Colaiácovo, 2018). Its assembly is through integrating the CR proteins to connect two LEs, a poorly understood process that might differ in various organisms due to the divergent SC component proteins (Cahoon and Hawley, 2016).

In mice, a picture of how the SC proteins are assembled in order has been inferred from mouse knockout studies (Fraune et al., 2012; Geisinger and Benavente, 2017). After DNA replication in the pre-meiotic S-phase, each pair of sister chromatids are tightly held together by cohesin complexes. The chromatin of sister chromatids is organized in a linear array of loops emanating from the chromosome axis, forming the meiotic axis-loop organization, which allows the close juxtaposition of homolog axes during meiotic prophase (Zickler and Kleckner, 2015). During preleptotene stage, the AE/LE proteins: SYCP2 and SYCP3 load onto the cohesin complex together with the HORMA domain-containing proteins (HORMAD1 and HORMAD2) (Wojtasz et al., 2009), forming the chromosome axis during meiotic prophase I (Zickler and Kleckner, 1999; Yuan et al., 2000; Yang et al., 2006; Fujiwara et al., 2020). Recent studies suggest that SYCP2 mediates the anchoring of chromatin loops to the axis by associating with the cohesin complex (Feng et al., 2017; Xu et al., 2019). Moreover, SYCP2 possesses putative ‘closure motifs’ that might be responsible for HORMADs recruitment (West et al., 2019). Then, the CR proteins: SYCP1, SYCE3, and SYCE1, which are essential for synapsis initiation, are assembled between the AEs in sequence: the TF protein SYCP1 first associates with the AEs, likely through interacting with SYCP2 (Winkel et al., 2009; Schücker et al., 2015); and then recruits SYCE3 through direct interaction (Schramm et al., 2011; Hernández-Hernández et al., 2016). Subsequently, SYCE1 is loaded likely through interacting with SYCE3 (Lu et al., 2014). SYCE1 also interacts with and stabilizes SYCP1 (Costa et al., 2005). Recently, a novel CE protein, SIX6OS1, has been shown to be required downstream of SYCP1 at a similar hierarchy level to SYCE3 (Gómez -H et al., 2016b). Finally, synapsis spread along the entire length of homolog axes with the required loading of SYCE2 and TEX12 (Hamer et al., 2006). These proteins interact with the SC through SYCE2 binding to SYCP1, SYCE3, and SYCE1 (Costa et al., 2005; Bolcun-Filas et al., 2007; Schramm et al., 2011) and interact interdependently to promote the assembly and stabilization of the SC (Cahoon and Hawley, 2016; Geisinger and Benavente, 2017). All these CR proteins are required for fertility in female and male mice, unlike LE proteins, whereas knockout SYCP2 or SYCP3 leads to sterility in males but subfertility in females (Yang et al., 2006; Bolcun-Filas et al., 2007; Gómez-H et al., 2016).

The SC is completely assembled between all the lengthwise-aligned homologs at the pachytene stage. Interestingly, this SC structure is highly dynamic during early pachytene in yeast and C. elegans as the SC subunit composition are constantly changing (Voelkel-Meiman et al., 2012; Pattabiraman et al., 2017) and shifts to a more stable state in late pachytene as recombination progresses.

After CO formation, the SC disassembles as SYCP1 is lost from chromosome arms in diplotene. However, SC fragments remain at centromeres and CO sites, presumably to coordinate local chromosome organization and separate the homologous axes, until diakinesis (Bisig et al., 2012; Qiao et al., 2012). After removing SYCP1 from the centromeres, SYCP3 accumulates and persists in these regions until late diplotene, before the nuclear-envelope breakdown, likely to promote proper homologous centromere bi-orientation, ensuring appropriate homolog segregation (Bisig et al., 2012; Qiao et al., 2012).

Multiple layers of regulation are imposed on the formation and disassembly of the SC to coordinate these mechanisms with the MR in various organisms (Zickler and Kleckner, 2015; Gao and Colaiácovo, 2018). These include the regulation from structural axial protein (cohesin and HORMADs), the transcriptional regulation of the SC genes, translational control of SC proteins mRNAs, the association of non-structural regulators with SC components, protein modifications, *etc.* (Zickler and Kleckner, 2015; Gao and Colaiácovo, 2018).

In mice, AE formation depends on meiotic cohesion with different contributions from different cohesin (reviewed in (Ishiguro, 2019). HORMAD1 is essential for both homolog pairing and synapsis (Shin et al., 2010; Kogo et al., 2012a; Paigen and Petkov, 2018) as HORMAD1 promotes efficient DSB formation and enables DSB-mediated homology search (Shin et al., 2010; Kogo et al., 2012a; Paigen and Petkov, 2018). Moreover, HORMAD1 might also have a direct role in SC formation (Paigen and Petkov, 2018). Additionally, both HORMAD1 and HORMAD2 are required to surveil homolog synapsis (Wojtasz et al., 2009; Kogo et al., 2012b; Paigen and Petkov, 2018). Their absence rescues the loss of asynaptic oocytes in the SPO11-deficient background (details below).

In yeast, synapsis initiation is controlled by the ‘ZMM’ proteins. Also, the SUMOylation of several SC components is required for SC assembly (Humphryes et al., 2013; Leung et al., 2015). In mice, SC initiation depends on the total number of interhomolog engagements. Reduced DSBs levels lead to fewer interhomolog engagements, causing delayed synapsis (Kauppi et al., 2013). Whether SUMOylation is involved in SC assembly in mice is unclear, although similar to yeast Red1, mouse SYCP3 can also be SUMOylated (Xiao et al., 2016). A positive feedback system in yeast controls SC polymerization. The initial assembly of the transverse filament recruits central-element proteins, which recruit more transverse filaments. The mechanism controlling SC polymerization in mice remains unknown (Cahoon and Hawley, 2016). The control of the timing between the formation of a CO and SC disassembly is vital for proper chromosome segregation. In mice, this relies on cell-cycle kinases (PLK1, Aurora B, CDK1-Cyclin B1), which are regulated through transcriptional and translational mechanisms (Gao and Colaiácovo, 2018).

### 2.2 Meiotic recombination

Meiotic recombination is homologous recombination (HR)-where the homologous chromosomes are used as the template for DSB repair, generating NCO and CO products and impacting several other meiotic events during meiosis (Keeney, 2008; Lam and Keeney, 2015). In many organisms, including mammals, MR promotes the close juxtaposition of each pair of homologous chromosomes, thus facilitating chromosome synapsis. DSB-mediated interhomolog interactions generate CO products in the context of synapsed chromosomes, resulting in the exchange of alleles between homologs. Besides, COs facilitate the proper orientation of homologous pairs at metaphase and thus ensure they segregate accurately at the first meiotic division, eventually supporting functional gametes formation (Hunter, 2015; Lam and Keeney, 2015; Marsolier-Kergoat et al., 2018) (Figure 1).

**FIGURE 2:**
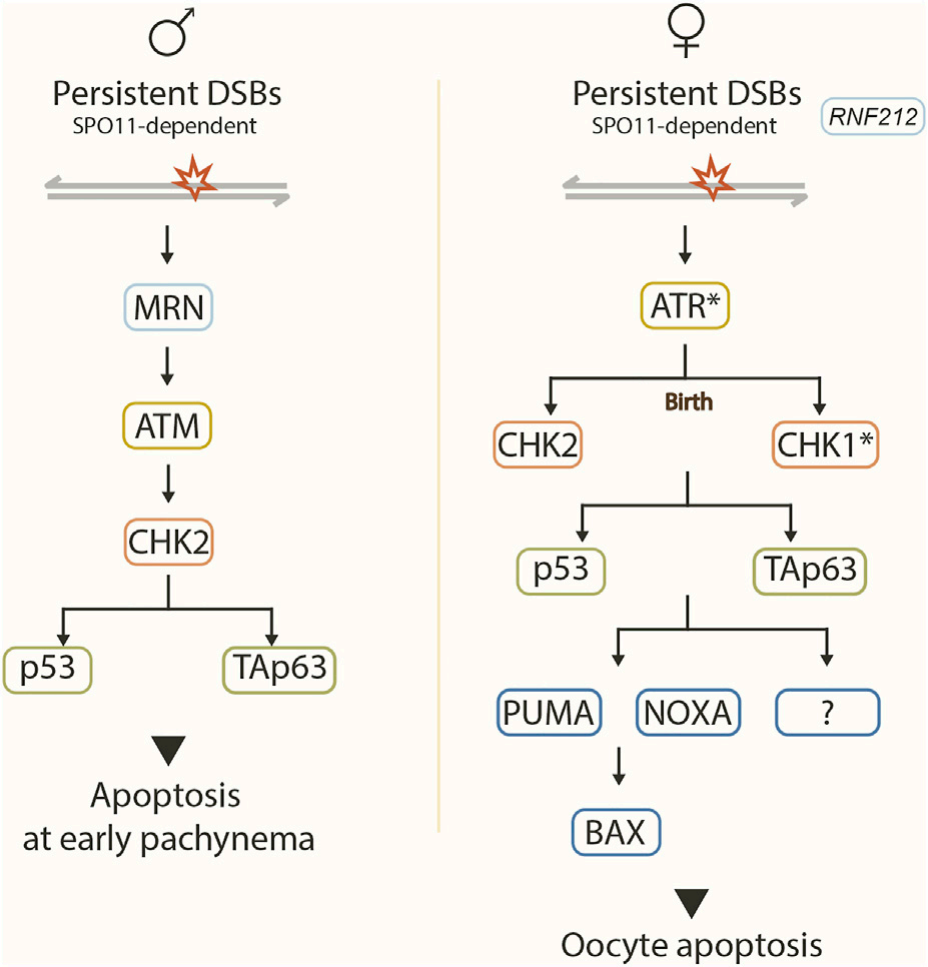
Recombination checkpoint pathway. Model showing the proposed signaling pathway in response to unrepaired SPO11-dependent DSBs in male (left) and female (right) mice. Asterisks represent predicted checkpoint factors. In males, DSBs are sensed by the MRN complex, leading to the activation of ATM, which in turn activates effector CHK2. CHK2 acts on target proteins p53 and TAp63, which implement the recombination-dependent arrest that blocks progression to mid/late pachynema. In females, RNF212 prevents the repair of residual DSBs in the late prophase. Unrepaired DSBs likely activate ATR, which may activate CHK2 before birth and CHK1 after birth. CHK1 and CHK2 signal to p53 and TAp3. Pro-apoptotic BCL-2 pathway components PUMA, NOXA, BAX, and other unknown factors act downstream to trigger oocyte apoptosis.

**FIGURE 3:**
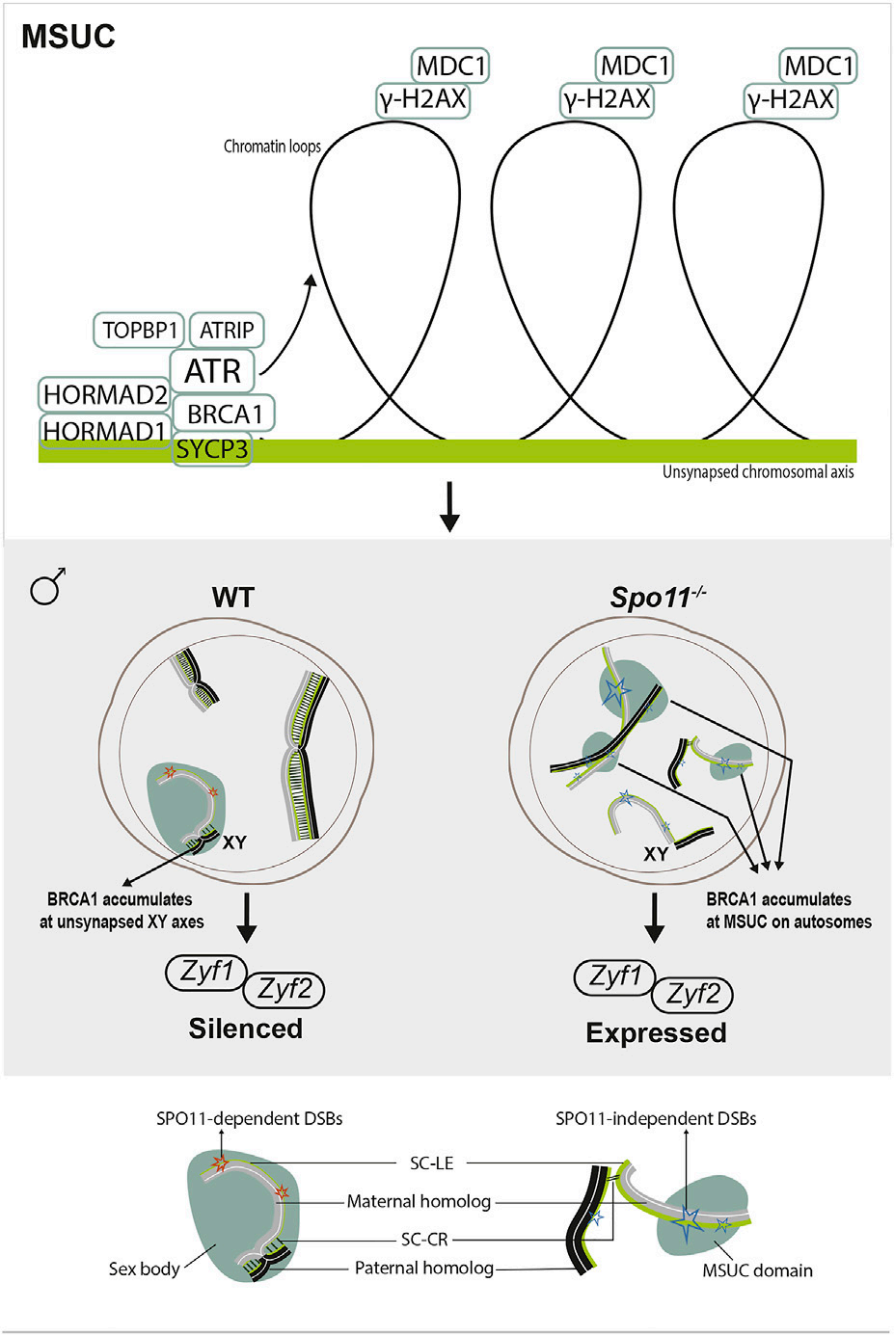
Meiotic response to asynapsis in male. Upper panel, meiotic silencing of the unsynapsed chromatin (MSUC). Axially located proteins signal asynapsis and recruit ATR with cofactors, such as BRCA1. If asynapsis persists, ATR translocates to chromatin loops, phosphorylating H2AX (γH2AX). This signaling spreads all over the chromatin with the help of MDC1, leading to the recruitment of silencing factors for the irreversible silencing of this region. Lower panel, in wild-type spermatocytes, the physiological MSUC occurs at XY chromosomes (MSCI), resulting in the silencing of sex chromosome-linked lethal genes (*Zfy1* and *Zfy2*)*.* However, in *Spo11* ^*−/−*^ spermatocytes, despite extensive asynapsis, localized MSUC are triggered by SPO11-independent DSBs on unsynapsed autosomes. As BRCA1 accumulates at these DSB sites, MSCI fails to form, and lethal genes are expressed.

**FIGURE 4:**
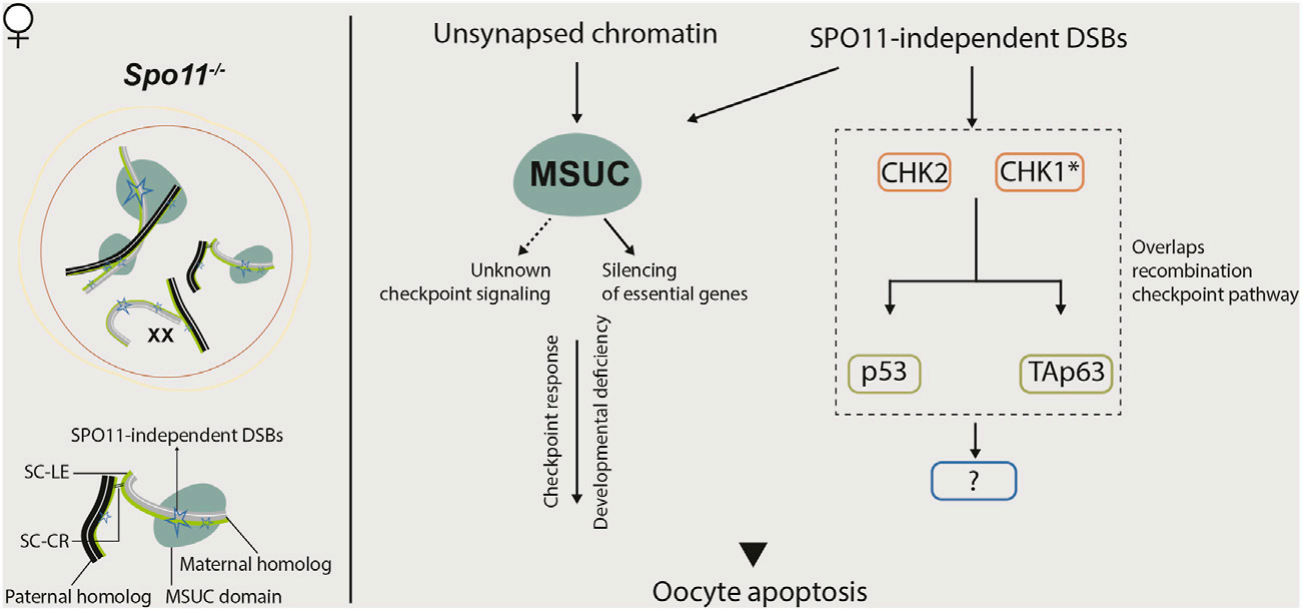
Meiotic response to asynapsis in female. Several mechanisms are proposed to be responsible for eliminating *Spo11* ^*−/−*^ oocytes. MSUC might trigger an unknown checkpoint signaling pathway in these oocytes or silence essential genes for development, leading to oocyte apoptosis. In parallel, in response to SPO11-independent DSBs, CHK2-mediated DNA damage signaling, which partially overlaps the recombination checkpoint pathway, also contributes to the elimination of *Spo11* ^*−/−*^ oocytes.

MR initiates when numerous programmed DSBs are induced by the conserved SPO11 protein, the ortholog of subunit A of TopoVI DNA topoisomerase (TopoVIA) (Bergerat et al., 1997; Keeney et al., 1997). It catalyzes DNA cleavage *via* a transesterification reaction, generating meiotic DSBs covalently bound by SPO11 at the 5′end (De Massy et al., 1995; Liu et al., 1995) (Figures 1–5).

In many organisms, accessory DSB proteins are also required for SPO11-mediated DSB formation (Lam and Keeney, 2015). Notably, a TopoVIB-like subunit (TOPOVIBL), structurally similar to the TopoVIB subunit of Topo VI topoisomerase, is also essential for meiotic DSB formation in mice and most likely in most eukaryotic species (Robert et al., 2016; Vrielynck et al., 2016).

In budding yeast, nine other accessory proteins form different subcomplexes, directly or indirectly interacting with SPO11, and are all required for DSB formation, including Ski8, Rec102-Rec104 complex, Rec114-Mei4-Mer2 complex, Mre11–Rad50–Xrs2 (MRX) complex (Lam and Keeney, 2015; Yadav and Claeys Bouuaert, 2021). In mice, three evolutionarily conserved proteins have also been identified to be required for SPO11-mediated DSB formation, including IHO1, MEI4, and REC114, the mouse orthologs of yeast Mer2, Mei4, and Rec114, respectively. These three proteins colocalize on the axes of the meiotic chromosome independently of SPO11 activity (Kumar et al., 2010; Stanzione et al., 2016; Kumar et al., 2018).

IHO1 is a direct interactor of the axial component protein HORMAD1 in mice (Stanzione et al., 2016). It is required for the axis-localization of REC114 and MEI4 *in vivo*. However, its axial localization is independent of MEI4 or REC114. Thus, IHO1 might act as a platform to recruit REC114 and MEI4 to the axes (Stanzione et al., 2016; Kumar et al., 2018). REC114 directly interacts with TOPOVIBL in mice, regulating the SPO11/TOPOVIBL catalytic activity (Nore et al., 2022). It is also inferred to perform this function *via* ATM-dependent inhibition of DSBs (Subramanian and Hochwagen, 2014; Boekhout et al., 2019). ATM might target REC114 directly by phosphorylating it, as in *S. cerevisiae* (Carballo et al., 2013), or indirectly by phosphorylating ANKRD31, a novel interactor of REC114 (Boekhout et al., 2019; Papanikos et al., 2019).

DSBs are non-randomly distributed along the chromosomes. They tend to accumulate preferentially at regions called recombination hot spots (Székvölgyi et al., 2015), which are determined by PRDM9 in most mammals (Paigen and Petkov, 2018). PRDM9 binds to specific DNA sequences in the genome through its zinc finger array. It then methylates histone H3 lysines 4 and 36 (H3K4me3 and H3K36me3) of nearby nucleosomes using its PR/SET domain, activating hot spots (Grey et al., 2018). Activated hot spots are believed to mainly locate at the DNA loops. It is not fully understood how they are further associated with the chromosomal axis where SPO11 and the accessory proteins are located. Studies have speculated that EWSR1, CDYL, EHMT2, and CXXC1 proteins might mediate this association through binding the KRAB domain of PRDM9 and interacting with the DSB proteins (Imai et al., 2017; Parvanov et al., 2017). As a result, PRDM9 targets SPO11 to specific genome regions, generating DSBs. Nevertheless, some DSB sites are targeted independently of PRDM9 in meiosis, e.g., the pseudoautosomal region (PAR) in male meiosis (Brick et al., 2012).

DSB formation is tightly controlled to occur in a narrow time window within prophase I, and in yeast, ATM plays an essential role in this by regulating further DSB formation *via* a negative feedback loop both in trans and cis (Barchi et al., 2008; Lange et al., 2011; Zhang et al., 2011; Garcia et al., 2015; Pacheco et al., 2015). Depleting ATM leads to significantly increased DSBs in multiple organisms, including mice (Joyce et al., 2011; Lange et al., 2011; Kurzbauer et al., 2012; Pacheco et al., 2015). ATM might prevent repeated DSB formation at the same chromosomal locus in mice as in yeast (La Salle and Trasler, 2006; Barchi et al., 2008; Lange et al., 2011; Garcia et al., 2015; Lukaszewicz et al., 2021). Besides**,** ATM might be involved in other feedback circuits to ensure enough DSBs are formed to support homolog interactions and recombination (Cooper et al., 2014).

After DSB formation, DSB ends are resected to generate ssDNA tails (Baudat et al., 2013; Lam and Keeney, 2015) (Figure 1).

The DSB resection is well elucidated in budding yeast. The MRX complex recognizes DNA-bound Spo11 and generates nicks nearby with Sae2, leading to the release of Spo11 bound to short oligonucleotides (*Spo11 oligos*) (Neale et al., 2005; Cannavo and Cejka, 2014). The nicks serve as entry points for short-range 3′→5′resection, mediated by Mre11 exonuclease activity, and long-range 5′→3′resection, mediated by Exo1 exonuclease activity and Dna2 nuclease (Manfrini et al., 2010; Zakharyevich et al., 2010; Garcia et al., 2011). The consequence is the generation of 3′ssDNA tails on both sides of the DSB. The full-length resection requires the DSB-responsive kinase Tel1, which promotes resection initiation, likely through Sae2 phosphorylation (Cartagena-Lirola et al., 2008), and regulates resection length (Mimitou et al., 2017).

In mammals, EXO1 is dispensable for DSB resection (Wei et al., 2003), and the nucleotide-excision repair factor, DNA polymerase-β, is implicated in SPO11 removal (Kidane et al., 2010). However, the role of the mammalian MRX complex and Sae2 homologs, the MRN complex (MRE11-RAD50-NBS1) and CtIP, respectively, in meiotic DSB repair is poorly understood due to the embryonic lethality of knocked-out mice of any MRN component (Pacheco et al., 2015; Zhang et al., 2020a). A recent study has demonstrated that conditional disruption of NBS1 in mouse testis causes a dramatic reduction of DNA end resection and severe defect in chromosome synapsis, eventually leading to meiotic arrest and infertility (Zhang et al., 2020a). Thus, like MRX in yeast, the MRN complex is likely essential for mammalian DSB resection.

Resected 3′ssDNA tails are immediately bound by replication protein A (RPA) and RPA1-related protein MEIOB and its associated factor, SPATA22. The recombinases DMC1 and RAD51 further replace these. Then, one of the RAD51/DMC1-coated ssDNA commences engaging in homology search and interhomolog interactions. Consequently, unstable nascent D-loop intermediates are likely generated *in vivo*. These are either destabilized in the NCO pathway or stabilized in the CO pathway (Brown and Bishop, 2015; Hunter, 2015) (Figure 1).

DMC1 and RAD51 are strand-exchange proteins. RAD51 functions in somatic and meiotic cell cycles, whereas DMC1 is meiosis-specific (Brown and Bishop, 2015). DMC1 is the essential DNA strand–exchange factor in meiosis, while RAD51 could be dispensable but performs a critical regulatory role in yeast and mammals (Cloud et al., 2012; Hinch et al., 2020).

The assembly of both recombinases is ATP-dependent and promoted by several recombination factors in mammals such as ATR, breast cancer 2 protein (BRCA2), TRIP13, the Shu complex SWS1-SWSAP1, and PALB2, *etc.* (Zelensky, Kanaar, and Wyman, 2014; Abreu et al., 2018; Roig et al., 2010; Pacheco et al., 2018; Felipe-Medina et al., 2020; Zhang et al., 2020b; Zhang et al., 2019a; Widger et al., 2018). Several recent studies identified BRCA2 localizer (MEILB2) and MEILB2’s stabilizer (BRME1), both of which form a complex with BRCA2 and function as the recruiter of RAD51 and DMC1 onto ssDNA (Zhang et al., 2019a; 2020b; Felipe-Medina et al., 2020). The activity of the DMC1-RAD51 complex to promote homology search and strand exchange is driven by the stability of the formed nucleoprotein filament (Brown and Bishop, 2015), which is enhanced by the HOP2-MND1 complex (Petukhova et al., 2003; Petukhova et al., 2005; Chi et al., 2007; Pezza et al., 2007).

In stark contrast to the exclusive inter-sister (IS) recombination interactions occurring in the somatic cell cycle, MR interactions are biased towards homologous chromosomes, thereby promoting pairing, synapsis, and formation of chiasmata between homologous chromosomes. The precise mechanism of this meiotic inter-homolog (IH) bias is unclear but is likely achieved both by inhibiting IS bias and promoting IH bias. The so-far best-understood mechanism was uncovered in yeast, involving Tel1/Mec1 (ATM/ATR), Hop1 (homolog of HORMAD1/2), effector kinase Mek1 (homolog of CHK2), and RAD54, an SWI/SNF-family ATPase (Subramanian and Hochwagen, 2014).

In the contemporary meiotic recombination models that are largely built on yeast studies, single-strand invasions result in less stable nascent joint molecules, presumably D-loops. The differentiation of D-loops leads to either NCOs *via* synthesis-dependent strand annealing (SDSA) or class I COs subject to interference (details of CO interference will be discussed below) *via* forming CO-specific intermediates single-end invasions (SEIs) and double Holliday junctions (dHJs) (Figure 1). D-loops are stabilized along the CO pathway to form SEIs, which are the earliest detectable CO-specific joint molecules. Subsequently, SEIs become more stable dHJs joint molecules through a second-end capture coupled with DNA synthesis. Eventually, dHJs are resolved exclusively into class I COs. By contrast, unstable D-loops are not stabilized in the NCO pathway after the invading strand extends. The nascent DNA is annealed to the other end of the broken DNA molecule resulting in NCOs. Additionally, a minority of D-loops escape from these two pathways and generate NCOs and non-interfering class II COs (Baudat et al., 2013; Hunter, 2015; Ranjha et al., 2018).

The differentiation of the CO and NCO pathways is controlled by a panel of factors through processing and stabilizing the recombination intermediates, including ZMM proteins and a helicase complex, STR/BTR (yeast Sgs1–Top3–Rmi1, metazoan BLM-TOPIIIα-RMI1-RMI2) (Hunter, 2015). ZMMs stabilize recombinational joint molecules and promote the formation of SC, ultimately required for class I CO formation. In budding yeast, ZMMs are CO-specific. However, in mice and several other species, ZMMs’ stabilization of recombinational interactions may be a prerequisite for CO designation, and D-loops bound by ZMMs could also form NCOs products (De Vries et al., 1999; Edelmann et al., 1999; Kneitz et al., 2000; Higgins et al., 2008; Yokoo et al., 2012; De Muyt et al., 2014; Zhang et al. Liang, 2014).

ZMM is a group of functionally diverse proteins, and several mammalian ZMM proteins have been identified to have a role in the CO/NCO decision: MSH4, MSH5, TEX11, RNF212, HEI10, HFM1, SHOC1, and SPO16. All of these proteins partially colocalize with recombination foci (defined by RAD51 and DMC1) on synapsed axes (De Vries et al., 1999; Edelmann et al., 1999; Kneitz et al., 2000; Adelman and Petrini, 2008; Guiraldelli et al., 2013; Guiraldelli et al., 2018; Qiao et al., 2014; Prasada Rao et al., 2017; Zhang et al., 2019b).

MSH4 and MSH5 are homologs of the bacterial MutS family of mismatch repair proteins with no known function in mismatch repair and form the MutSγ heterodimer (Pochart et al., 1997), which is essential for chromosome synapsis, CO formation, and thus fertility in mice (De Vries et al., 1999; Edelmann et al., 1999; Kneitz et al., 2000). HFM1 is essential for mammalian fertility as mutated HFM1 was found in human patients with azoospermia or POI syndromes (Baudat et al., 2013; Wang et al., 2014; Zhang et al., 2017) and removing HFM1 causes a drastic reduction of COs and partially affects synapsis in mice (Guiraldelli et al., 2013). The deficiency of SHOC1, TEX11, and SPO16 causes reduced COs with a relatively minor synapsis defect in mice, suggesting a conserved role in CO formation as in yeast (Adelman and Petrini, 2008; De Muyt et al., 2018; Guiraldelli et al., 2018; Zhang et al., 2019b). Mouse RNF212 and HEI10, a ubiquitin-ligase, regulate CO by modifying recombination factors (MutSγ) at CO-designated sites in an antagonistic manner. Subsequently, stabilized recombination factors enable the recruitment of CO-specific factors (MLH1-MLH3, MutLγ) for CO maturation (Reynolds et al., 2013; Qiao et al., 2014; Hunter, 2015; Gray and Cohen, 2016; Prasada Rao et al., 2017).

During early recombination steps, STR/BTR is required for channeling early joint molecules into CO and non-CO pathways. Later, STR/BTR promotes the resolution of the final recombination intermediates into NCOs by its dissolution activity *via* SDSA (Hunter, 2015). Distinguishingly, in the CO pathway, the resolution of joint molecules is mediated by the endonuclease activity of mismatch repair factors MLH1, MLH3, and EXO1 to generate class I COs. MLH1 and MLH3 are invaluable markers of crossovers in the cytological analysis as they localize precisely to future CO sites in many organisms (Kolas and Cohen, 2004). Additional factors are found to be required for class I CO formation in mice, including HEI10 (discussed above), CNTD1, PRR19 and CDK2 (Holloway et al., 2014; Qiao et al., 2014; Bondarieva et al., 2020).

For non-interfering class II COs, the resolution of joint molecules is mediated by structure-specific endonucleases, Mus81, Yen1, and Slx1/4 in yeast and MUS81 in mice (Holloway et al., 2008; De Muyt et al., 2012; Zakharyevich et al., 2012). In mice, interfering COs are estimated to account for ∼90% of COs (Holloway et al., 2008; Serrentino and Borde, 2012), and consistently, the deletion of MLH1, MLH3, or EXO1 causes significant loss of chiasmata and, consequently, mice sterility (Baker et al., 1996; Edelmann et al., 1996; Lipkin et al., 2002; Wei et al., 2003).

Finally, another layer of control tightly regulates the outcome of DSB repair. CO numbers per meiosis show a low variation despite a much more considerable variation in the numbers of recombinational interactions. This phenomenon is called CO homeostasis, which is underpinned by the lower and upper limits for the CO numbers regulated by CO assurance and interference (Martini et al., 2006; Rosu et al., 2011; Cole et al., 2012; Yokoo et al., 2012; Hunter, 2015). CO assurance guarantees that each homolog pair obtains at least one CO to segregate properly at meiosis I. Meanwhile, interference is defined by an inhibitory zone around CO-designated sites where other DSBs are prevented from becoming COs. Interference results in COs being widely and evenly spaced along the genome (Hillers, 2004; Berchowitz and Copenhaver, 2010; Zhang and Liang, 2014).

The molecular mechanisms responsible for CO assurance and interference have been long elusive. Studies in various species have described different mechanisms regulating CO interference (Zhang and Liang, 2014; Zhang and Wang, 2014; Fowler et al., 2018; Capilla-Pérez et al., 2021; France et al., 2021; Morgan et al., 2021). A study of fission yeast *S. pombe* suggests a clustering model, emphasizing DSB interference as the basis for CO interference (Fowler et al., 2018). In this model, in each cluster containing several DSB hotspots, only one single DSB is formed. Given that DSBs are the precursors to COs, consequently, at most, a single CO is made in the chromosomal interval corresponding to the DSB hotspot-clustered interval (Fowler et al., 2018). Studies in budding yeast described a stress-and-stress relief mechanism for CO interference (the ‘beam-film’ model), which is SC independent and requires topoisomerase II (Zhang and Liang, 2014; Zhang and Wang, 2014). Distinctly, recent work in *Arabidopsis* demonstrated that the SC is essential for CO interference (Capilla-Pérez et al., 2021; France et al., 2021). Finally, a new model (the diffusion-mediated coarsening model) is proposed to explain CO interference (Morgan et al., 2021). These models may apply to some but likely not all species since the mechanism and control of meiotic recombination varies among species.

How the outcome of DSB repair is regulated in mice is poorly understood, and ATM may have a role in forming the obligate CO in the small pseudoautosomal region of homology between sex chromosomes and controlling the numbers and distributions of COs on autosomes (Barchi et al., 2008). However, this molecular mechanism elucidated in *S. pombe* is likely conserved in diverse organisms, including flies and mice, based on the features of meiotic recombination and pericentric regions in these species (Prieto et al., 2001; Manheim and McKim, 2003; Fukuda et al., 2012; Bhattacharyya et al., 2019; Hartmann et al., 2019; Smith and Nambiar, 2020).

### 2.3 Meiotic prophase surveillance mechanisms

DSB repair and synapsis are carefully monitored during the meiotic prophase to choreograph nuclear dynamics and cell division programs. An intricate meiotic checkpoint network has emerged to create dependencies between independent processes when homologous chromosomes pair, synapse, and recombine. The machinery of this meiotic checkpoint involves many canonical DNA damage response (DDR) signaling proteins, among which the two evolutionarily conserved sensor kinases, ATM and ATR, play a central role (MacQueen and Hochwagen, 2011). They detect and respond to DSBs with the help of checkpoint cofactors in many organisms. Once activated, ATM and ATR phosphorylate a large set of substrates, preferentially containing serine/threonine-glutamine (S/TQ) cluster domains (Traven and Heierhorst, 2005). Many of these target proteins act directly to implement the checkpoint response, while others work as transmitters to relay the checkpoint signals to downstream effectors, such as CHK1 and CHK2 kinases (Subramanian and Hochwagen, 2014). This section will discuss how the surveillance mechanisms of the meiotic prophase checkpoint monitor these meiotic events, particularly in mammals.

In response to DSB repair or synapsis defects, the cells trigger a cell cycle arrest at the pachytene stage to provide sufficient time to fix the errors. If errors persist, this mechanism can eventually activate apoptosis to cull meiocytes in various organisms (Roeder, 2000; Bhalla and Dernburg, 2005; Di Giacomo et al., 2005; Lu et al., 2010)**.** In mammals, observations in mutant mice deficient in meiotic recombination suggest that two genetically distinct surveillance mechanisms contribute to the activation of the arrest in both males and females: the recombination (DNA damage) checkpoint monitoring the DSB repair process and the synapsis checkpoint monitoring SC formation (Roeder, 2000; MacQueen and Hochwagen, 2011; Subramanian and Hochwagen, 2014; Joshi et al., 2015).

In males defective in DSB repair, like *Trip13* ^*mod/mod*^ and *Dmc1* ^*−/−*^ mice, most spermatocytes arrest before incorporating the testis-specific histone 1t (H1t) at pachynema (Barchi et al., 2005; Marcet-Ortega et al., 2017; Testa et al., 2018). In contrast, *Spo11* ^*−/−*^ spermatocytes, which do not have programmed DSBs, incorporate H1t and progress further, reaching mid/late pachytene. These cells arrest before completing the meiotic prophase and ultimately apoptose (Barchi et al., 2005; Pacheco et al., 2015). Therefore, irrespective of the common apoptosis consequence, spermatocytes respond differently to these two meiotic defects. Furthermore, the removal of DSBs confers a *Spo11*-like phenotype to those DSB repair-deficient mutants (*Dmc1* ^*−/−*^ and *Trip13* ^*mod/mod*^) (Barchi et al., 2005; Li and Schimenti, 2007), indicating that separate checkpoints act sequentially to mediate the apoptosis of these defective spermatocytes.

Likewise, in females, the elimination of oocytes defective for DSB repair (*Trip13* ^*mod/mod*^) or both DSB repair and synapsis (*Dmc1* ^*−/−*^, *Msh5* ^*−/−*^) occurs earlier (around birth) than those defective for synapsis alone (*Spo11* ^*−/−*^, up to 2 months *postpartum*) (Di Giacomo et al., 2005; Li and Schimenti, 2007). Also, mutations disrupting DSB formation (*Spo11* and *Mei1*) are epistatic to those affecting DSB repair (*Dmc1*, *Atm*, *Trip13*, and *Mcmdc2*) (Di Giacomo et al., 2005; Reinholdt and Schimenti, 2005; Li and Schimenti, 2007; Finsterbusch et al., 2016; Martínez-Marchal et al., 2020). These lines of evidence further support the existence of two distinct checkpoint mechanisms in mammals, sensing DNA damage or synapsis errors and resulting in meiotic prophase arrest. However, there are also arguments against a specific “synapsis checkpoint”, at least in females, favoring that a canonical DNA damage checkpoint primarily accounts for the oocyte loss in response to both recombination and synapsis defects (discussed below) (Rinaldi et al., 2017; Rinaldi et al., 2020).

#### 2.3.1 The recombination checkpoint

The recombination checkpoint is likely activated when recombination intermediates persist at pachynema in mammals (Di Giacomo et al., 2005; Burgoyne et al., 2009; MacQueen and Hochwagen, 2011). So far, the study of the recombination checkpoint in mammals has been challenged because most mutations that compromise recombination also affect synapsis. However, a gene-trap-disrupted allele of *Trip13*, *Trip13* ^*mod/mod*^ (also known as *Trip13* ^*RRB047RRB047*^, X. Li and Schimenti, 2007; Roig et al., 2010), which cannot repair DSBs but completes synapsis, has proven to help study the recombination-dependent arrest and meiocyte elimination. Analyses of mice doubly or triply deficient for TRIP13 and other DDR genes uncovered several signaling pathways involved in the recombination checkpoint-mediated arrest and/or apoptosis in both males and females (Bolcun-Filas et al., 2014; Pacheco et al., 2015; Marcet-Ortega et al., 2017; Rinaldi et al., 2017; Rinaldi et al., 2020).

In males, the MRN complex, ATM, CHK2, and the p53 family members, p53 and TAp63, are required to arrest spermatocytes with unrepaired DSBs at early pachynema before incorporating H1t into the chromatin (Pacheco et al., 2015; Marcet-Ortega et al., 2017; Marcet-Ortega et al., 2022) (Figure 2).

In *Trip13* ^*mod/mod*^ mice, spermatocytes enter pachynema with homologous chromosomes completely synapsed but with persisting recombination intermediates. Thus, most spermatocytes arrest and undergo apoptosis at epithelial stage IV before incorporating the mid-pachytene histone marker H1t (Li and Schimenti, 2007; Roig et al., 2010; Pacheco et al., 2015). In comparison, in *Trip13* ^*mod/mod*^ *Spo11* ^*+/−*^ *Atm* ^*−/−*^ triple mutant mice, where the activity of ATM is removed, a significant proportion of spermatocytes accumulate H1t despite containing high levels of unrepaired DSBs. Thus, eliminating ATM activity allows spermatocytes to progress further, from an H1t-negative to an H1t-positive stage, despite having significant amounts of unrepaired DSBs. These findings suggest that ATM may be required for the recombination-dependent arrest at early pachynema (Barchi et al., 2005; Pacheco et al., 2015).

The MRN complex is responsible for DSBs sensing and activating ATM in somatic cells (Stracker and Petrini, 2011). It is also required for meiotic recombination in many organisms, including mammals (Keeney and Neale, 2006; Cherry et al., 2007). CHK2 is an effector kinase of the ATM signaling pathway activated in response to ionizing radiation (Matsuoka et al., 1998). Interestingly, disruption of the MRN complex or the CHK2 kinase in *Trip13* ^*mod/mod*^ mutants confers a meiotic progression phenotype similar to *Trip13* ^*mod/mod*^ *Spo11* ^*+/−*^ *Atm* ^*−/−*^ mutants (Pacheco et al., 2015). Thus, the MRN-ATM-CHK2 signaling cascade is likely to respond to persistent unrepaired DSBs, mediating the recombination-dependent pachytene arrest in male mice (Pacheco et al., 2015). Similarly, p53 and TAp63, two canonical CHK2’s downstream targets (Lu et al., 2010; Bolcun-Filas et al., 2014), have been inferred to act in the recombination-dependent arrest mechanism. This is based on the observations that *Trip13* ^*mod/mod*^ *p53* ^*−/−*^ and *Trip13* ^*mod/mod*^ *TAp63* ^*−/−*^ spermatocytes can progress to an H1t-positive stage (Marcet-Ortega et al., 2017).

In *Trip13* ^*mod/mod*^ mutants lacking ATM or with defective MRN complex, spermatocytes cannot correctly repair abundant DSBs caused by the disability of ATM’s negative feedback in DSBs formation (Lange et al., 2011; Pacheco et al., 2015). Thus these spermatocytes fail to complete synapsis, which impedes the sex body formation (Barchi et al., 2008; Burgoyne et al., 2009; Roig et al., 2010; Pacheco et al., 2015). On the contrary, in *Trip13* ^*mod/mod*^ *Chk2* ^*−/−*^, *Trip13* ^*mod/mod*^ *p53* ^*−/−*^, and *Trip13* ^*mod/mod*^ *TAp63* ^*−/−*^ spermatocytes, although the sex body is formed, sex chromosomes are not correctly silenced, which explains why these spermatocytes eventually undergo arrest and apoptosis at late pachynema (Pacheco et al., 2015; Marcet-Ortega et al., 2017). These lines of evidence further support an alternative arrest mechanism mediating sex body defects in male mice (Barchi et al., 2005) (discussed below).

In females, an ATR-CHK1/CHK2-p53/TAp63-PUMA/NOXA-BAX signaling pathway is proposed to mediate the DNA damage checkpoint response in the oocytes (Bolcun-Filas et al., 2014; Rinaldi et al., 2017; ElInati et al., 2020; Martínez-Marchal et al., 2020; Rinaldi et al., 2020) (Figure 2).

Deletion of CHK2 rescues developing oocytes in 3-week-postnatal *Dmc1* ^*−/−*^ mice, although the absence of primordial follicles eventually results in a nearly complete oocyte depletion by 2 months *postpartum*. This pattern of oocyte loss is highly similar to that in *Spo11* ^*−/−*^ or *Spo11* ^*−/−*^ *Dmc1* ^*−/−*^ mice, suggesting that the loss of CHK2 allows the deficient oocytes to surpass the DSB repair but not the synapsis arrest. Moreover, the deletion of CHK2 can reach a more successful rescue in *Trip13* ^*mod/mod*^ mice, which complete synapsis. *Trip13* ^*mod/mod*^ *Chk2* ^*−/−*^ mice have a significant pool of oocytes at 3 weeks *postpartum*, many follicles at 2 months of age, and sustained fertility for many months. Abundant γH2AX staining was detected in all dictyate *Trip13* ^*mod/mod*^ *Chk2* ^*−/−*^ oocytes indicating the persistence of unrepaired DSBs like in *Trip13* ^*mod/mod*^. Thus, CHK2 is required for the DNA-damage checkpoint-mediated oocyte elimination (Bolcun-Filas et al., 2014).

The lack of p53 and TAp63 enables nearly a complete rescue of *Trip13* ^*mod/mod*^ oocytes. Compared to wild-type mice, indistinguishable numbers of primordial and growing follicles are found in the triple mutant *Trip13* ^*mod/mod*^ *p53* ^*−/−*^ *TAp63* ^*−/−*^ mice (Rinaldi et al., 2020). Therefore, like in males, p53 and TAp63 might also act downstream of CHK2 in the DNA-damage checkpoint pathway in females. However, CHK2 deficiency only rescues *Trip13* ^*mod/mod*^ oocytes to around one-third of wild-type levels (Bolcun-Filas et al., 2014), implying other factors might signal these two effectors p53 and TAp63 in parallel with CHK2. Indeed, CHK1 is likely to perform this function (Martínez-Marchal et al., 2020; Rinaldi et al., 2020). Studies have shown that when CHK2 is absent in ovaries, CHK1 is activated by persistent DSBs and is responsible for eliminating *Chk2* ^*−/−*^ oocytes (Martínez-Marchal et al., 2020; Rinaldi et al., 2020) (Figure 2).

Interestingly, the pro-apoptotic BCL-2-dependent pathway acts downstream of CHK2/p53/TAp63 and eliminates recombination-defective oocytes (ElInati et al., 2020). The BCL-2-dependent pathway consists of the known targets of p53 and TAp63 PUMA, NOXA, and BAX (Su et al., 2013). PUMA and NOXA or BAX deletion rescue oocyte numbers in DSB-repair mutants (*Dmc1* ^*−/−*^ and *Msh5* ^*−/−*^). However, like CHK2 deletion, this rescue does not reach wild-type levels, indicating that other components of this pathway also control the oocyte population (Bolcun-Filas et al., 2014; Rinaldi et al., 2017; ElInati et al., 2020) (Figure 2). Indeed, other p53 targets (e.g., BAK, PERP, or CDKN1A) have been proposed to play a role in this mechanism (ElInati et al., 2020).

Non-etheless, the factors acting upstream of CHK2 in the recombination checkpoint pathway are not clearly understood in females. The loss of ATM triggers oocyte elimination by DNA damage checkpoint in mice, which can be rescued by the deficiency of CHK2 to a degree similar to the rescue by CHK2 in *Dmc1* ^*−/−*^ ovaries (Bolcun-Filas et al., 2014; Rinaldi et al., 2020). Thus, it has been proposed that ATR, the other canonical DDR kinase, activates CHK2 in the recombination checkpoint pathway in females.

Furthermore, RNF212, a SUMO ligase required for crossover formation, is also suggested to promote the apoptosis of DSB repair-defective oocytes since *Rnf212* deletion significantly restores the oocyte pool at 18 days *postpartum* in DSB-repair mutant females (*Msh4* ^*−/−*^) (Qiao et al., 2018). RNF212 is proposed to impede DSB repair *via* inter-sister recombination (IS-HR) by stabilizing the association of HORMAD1 along desynapsed chromosome axes during the late prophase. Thus, residual DSBs, the repair of which *via* IS-HR are prevented by RNF212, trigger CHK2-mediated DNA damage checkpoint, resulting in oocyte elimination (Qiao et al., 2018; ElInati et al., 2020).

Notably, in both spermatocytes and oocytes, a certain level of unrepaired DSBs is required to activate the recombination-dependent arrest pathways during the meiotic prophase (Marcet-Ortega et al., 2017; Rinaldi et al., 2017). This is particularly important in spermatocytes, where DSBs on the X chromosome arms lacking homologous partners are repaired using the sister chromatids at mid-late pachytene, later than on autosomes (Page et al., 2012; Baudat et al., 2013). Thus, the DSB threshold level for arrest activation must be high enough, or all wild-type spermatocytes would be arrested. Only spermatocytes reaching the threshold could activate both p53 and p63, which work independently but additively to trigger apoptosis response (Marcet-Ortega et al., 2017). In females, the primordial follicle pool is wholely abolished in wild-type ovaries when the newborn ovaries are exposed to more than 0.3 Gy of irradiation. This dosage induces 10.3 RAD51 foci per oocyte (Rinaldi et al., 2017). Therefore, like in males, a threshold level of DSBs also triggers cell arrest in females.

#### 2.3.2 The syapsis checkpoint

Defects in chromosome axis formation or SC assembly can activate a cell response to asynapsis independently of DSB formation in many organisms, leading to cell cycle arrest and even apoptosis (MacQueen and Hochwagen, 2011). In mammals, this synapsis checkpoint is debated: whether a specific surveillance mechanism monitoring asynapsis exists and how it senses it is unclear. Even if the synapsis checkpoint exists, it might not be like a formal checkpoint response (Cloutier et al., 2015; Turner, 2015; Rinaldi et al., 2017). In any case, the meiotic silencing of the unsynapsed chromatin (MSUC) plays a vital surveillance role in this so-called “synapsis checkpoint” in both males and females (Burgoyne et al., 2009; Cloutier et al., 2015; Turner, 2015).

The MSUC is a chromatin remodeling process by which unsynapsed regions are transcriptionally inactivated during meiotic prophase I (Turner, 2015) (Figure 3). It is achieved through the crosstalk between the axially located sensors signaling asynapsis, such as the axial component proteins HORMAD1/2 proteins (Wojtasz et al., 2009; Fukuda et al., 2010), and the loop-located effectors mediating gene silencing such as the histone variant H2AX (Fernandez-Capetillo et al., 2003)**.** HORMAD1 and HORMAD2 load onto chromosome axes at leptonema and are depleted from the axes by TRIP13 as the homologs synapse (Wojtasz et al., 2009; Fukuda et al., 2010; Roig et al., 2010; Koubova et al., 2014). By the late zygotene stage, HORMAD1/2 acts together with SYCP3 to recruit the breast cancer 1 (BRCA1) protein to the unsynapsed axes (Turner et al., 2004; Kouznetsova et al., 2009; Royo et al., 2013)**.** Then, in a HORMAD1/2- and BRCA1-dependent manner, ATR is recruited to unsynapsed axes (Turner et al., 2004; Wojtasz et al., 2012; Paigen and Petkov, 2018), which further promotes the enrichment of BRCA1 and ATR-activating cofactors: TOPBP1, ATRIP (Perera et al., 2004; Refolio et al., 2011; Royo et al., 2013) as well as regulate phosphorylation of HORMAD1/2 (Fukuda et al., 2012). If asynapsis persists until pachytene, ATR translocates into the chromatin loops and phosphorylates H2AX (γH2AX) with the help of the γ-H2AX-binding factor MDC1, resulting in the irreversible silencing of this region (Ichijima et al., 2011).

In males, spermatocyte loss mediated by the DSB-independent response to asynapsis involves the failure of Meiotic Sex Chromosome Inactivation (MSCI) (Burgoyne et al., 2009) (Figure 3). MSCI is a physiological MSUC process that responds to the unavoidable partial asynapsis of the sex chromosomes (Turner et al., 2006). MSCI is reflected by the formation of the sex body, a specialized subnuclear domain encompassing the asynapsed portions of the X and Y chromosomes in pachytene spermatocytes. The sex body is characterized by the lack of gene expression and sequestration of an array of proteins, which are primarily heterochromatin-related (e.g., H2A, H3meK9, CBX1/3) and recombination-related (e.g., MRE11, γH2AX, and RAD51) (Handel, 2004). In mutant mice with extensive asynapsis (e.g., *Spo11* ^*−/−*^, *Dmc1* ^*−/−*^), MSCI cannot occur, and the sex body fails to form, likely due to the limited association of silencing factors with the XY axes (Mahadevaiah et al., 2008; Kouznetsova et al., 2009). At the zygotene/pachytene transition in wild-type spermatocytes, as DSBs get repaired, BRCA1 is released from the DSB sites and accumulates at the HORMAD1-coated asynapsed XY axes, initiating MSCI response (Mahadevaiah et al., 2008; Burgoyne et al., 2009). However, in mutants with extensive asynapsis, BRCA1 is widely sequestered at unrepaired SPO11-dependent DSB sites (e.g., *Dnmt3l* ^*−/−*^), thus failing to form MSCI (Mahadevaiah et al., 2008) or accumulates at SPO11-independent DSB sites, randomly triggering localized MSUC response at autosomal axes (Carofiglio et al., 2013). As a result, lethal sex chromosome-linked genes (e.g., Zfy1 and Zfy2) are expressed, leading to spermatocyte progression arrest and apoptosis (Royo et al., 2010). Thus, physiological MSCI is required to allow the exit of the pachytene stage.

On the other hand, females possess two X chromosomes; thus, MSCI does not occur in the oocytes. So, the roles of the MSUC in response to asynapsis are different from that in males (Cloutier et al., 2015; Turner, 2015) (Figure 4).

In asynapsis models without associated recombination defects, such as *Spo11* ^*−/−*^ mice and mice harboring chromosome abnormalities, such as Turner syndrome (XO) with only one X chromosome, unsynapsed chromosomes undergo MSUC, and oocytes with these unsynapsed chromosomes are eliminated (Daniel et al., 2011; Wojtasz et al., 2012; Cloutier et al., 2015). Deletion of the MSUC factors HORMAD1 or HORMAD2 in *Spo11* ^*−/−*^ mice (Daniel et al., 2011; Wojtasz et al., 2012) or H2AX in XO mice (Cloutier et al., 2015) restores the oocyte numbers to wild-type levels. Thus, the MSUC is suggested to transduce asynapsis into germ cell arrest. The MSUC factors, HORMAD1 and HORMAD2 would be the putative synapsis checkpoint components in females (Turner, 2015).

However, it seems that the response to asynapsis in females ca not be simply explained only by this checkpoint signaling model. Other mechanisms are also proposed to account for the loss of oocytes harboring asynapsis: the MUSC might render oocytes deficient in multiple gene products required for oocyte survival and development (Cloutier et al., 2015; Cloutier et al., 2016).

In mouse models carrying extra/supernumerary chromosomes, oocytes with asynapsed chromosomes are not eliminated as in XO females, despite the presence of HORMAD1 and other meiotic silencing factors on the asynaptic supernumerary chromosomes (Cloutier et al., 2015). Silencing these asynaptic supernumerary chromosomes does not affect the normal gene expression from the entire genome. In contrast, asynapsis of chromosomes in XO or other chromosomally unbalanced females would likely lead to the silencing of multiple housekeeping genes, oogenesis-essential genes, or critical genes. Therefore, the fate of oocytes with asynapsis probably depends on the gene content of the silenced asynapsed chromosomes (Cloutier et al., 2015; Turner, 2015). In *Spo11* ^*−/−*^ oocytes, chromosomes are extensively unsynapsed, and the MSUC takes place on only a part of them (Carofiglio et al., 2013). This MSUC might silence some essential genes (e.g., oogenesis-essential genes), leading to oocyte arrest and ultimately triggering oocyte death. The rescue of oocyte loss by the deletion of silencing components HORMADS and H2AX in *Spo11* ^*−/−*^ mice and other asynapsis models (Daniel et al., 2011; Wojtasz et al., 2012; Cloutier et al., 2015) could be explained by the restoration of standard gene expression patterns, rather than the disruption of checkpoint signaling *per se* (Turner, 2015).

Recent findings show that the CHK2-dependent DNA damage checkpoint also culls SPO11-deficient oocytes (Rinaldi et al., 2017; Rinaldi et al., 2020). These data argue against the existence of a specific synapsis-checkpoint mechanism. Most *Spo11* ^*−/−*^ oocytes have some DSBs (Carofiglio et al., 2013; Malki et al., 2014). Thus, authors speculate that it could be enough to reach the threshold to trigger the CHK2-dependent recombination checkpoint (Rinaldi et al., 2017). So, a model in which two major mechanisms are responsible for the elimination of oocytes with synapsis defect is proposed: the meiotic silencing mechanism, as discussed above, which primarily works in oocytes with a small number of asynapsed chromosomes that carry meiotic-essential genes but the amount of unrepaired DSBs does not reach the threshold (Cloutier et al., 2015). The recombination checkpoint could function in oocytes with multiple asynapsed chromosomes (e.g., *Spo11* ^*−/−*^ oocytes) that accumulate a sufficient number of DSBs to trigger the checkpoint (Rinaldi et al., 2017; Rinaldi et al., 2020).

Interestingly, the CHK2 deficiency can only restore a limited number of *Spo11* ^*−/−*^ oocytes (Rinaldi et al., 2017; Martínez-Marchal et al., 2020). Also, HORMAD2 and CHK2 are not functioning in a single linear checkpoint pathway (Rinaldi et al., 2017; Martínez-Marchal et al., 2020). Therefore, other mechanisms eliminating most of the *Spo11* ^*−/−*^ oocytes cannot be excluded, for instance, through the MSUC mechanism and/or the CHK1-dependent DNA damage checkpoint (Rinaldi et al., 2017; Martínez-Marchal et al., 2020). Moreover, the lack of both p53 and TAp63 can protect nearly all *Spo11* ^*−/−*^ oocytes from elimination. However, the deletion of the BCL-2 components (PUMA, NOXA, and BAX) does not rescue the oocyte loss in *Spo11* ^*−/−*^ females (ElInati et al., 2020). These data suggest that at least two distinct and partially overlapping genetic signaling pathways likely respond to recombination and synapsis errors in females. Noticeably, a more recent study showed that RAD51 might not be a reliable DSB marker in oocytes, and although DNA damage signaling from asynaptic axes participates in removing *Spo11* ^*−/−*^ oocytes, it does not require high numbers of SPO11-independent DSBs as suggested in the study from Carofiglio et al (Carofiglio et al., 2013; Ravindranathan et al., 2022).

Collectively, compared to the recombination checkpoint, the genetic pathways responsible for the “synapsis checkpoint” control remain much less understood in both males and females. Rather than being a typical checkpoint, the surveillance mechanisms that respond to asynapsis in mammals might be more complex. At least in females, the DNA damage signaling pathway, the MSUC-mediated checkpoint signal-transducing, and the depletion of essential genes for oocyte development and survival might conspire to drive the elimination of oocytes with asynapsed chromosomes.

### 3 Genetic cause of infertility

Successful reproduction requires the precise regulation of complex processes essential for developing reproductive organs, performing gametogenesis, acquiring neuroendocrine competency, and the ability to carry a pregnancy (Yatsenko and Rajkovic, 2019). Infertility, a common, multifactorial pathological condition defined as the inability to establish a clinical pregnancy after at least 1 year of regular unprotected sexual intercourse, affects approximately 50 million couples worldwide (Mascarenhas et al., 2012). Among the infertility cases with identified causes, one-third is due to a female factor, another third is due to a male factor, and the remaining third is due to combined female and male factors (Mallepaly et al., 2017). Furthermore, genetic defects contribute to nearly 50% of these infertility cases. More unknown genetic causes are suggested in infertility and need to be uncovered (Zorrilla and Yatsenko, 2013).

Male infertility derives etiologically from quantitative spermatogenic defects, ductal obstruction or dysfunction, hypothalamic-pituitary axis dysfunction, and qualitative spermatogenic defects (from most to least common) (Tournaye et al., 2017). Genetic factors account for at least 15% of male infertility and involve all these etiological categories (Krausz and Riera-Escamilla, 2018). Diagnosing male infertility relies on semen (and hormone) analysis, which results in two major phenotypes: oligozoospermia (reduced sperm count) and azoospermia (no spermatozoa in the ejaculate) (Tüttelmann et al., 2018). Qualitative spermatogenic defects or ductal obstruction usually manifest as azoospermia, and multiple genetic factors are validated as the causes, including numerical and structural chromosomal anomalies (e.g., Klinefelter’s syndrome, 46, XX male syndrome), Y-chromosome micro-deletions (e.g., azoospermia factor (AZF) deletions), gene mutations (e.g., TEX11 deletions), and cystic fibrosis transmembrane conductance regulator (CFTR) mutations (Krausz and Riera-Escamilla, 2018). AZF deletions are the most frequent genetic cause of azoospermia (Krausz et al., 2014). Most numerical and structural chromosomal anomalies and TEX11 deletions are thought to cause spermatogenic defects due to errors during meiosis that activate the surveillance mechanisms (Sun et al., 2007; Yang et al., 2015; Yatsenko et al., 2015). Currently, some of these genetic infertility causes can be clinically diagnosed by widely applied analyses, such as karyotyping, AZF deletion screening, and CFTR mutation analysis (Tournaye et al., 2017).

Female infertility can result from a wide range of factors affecting ovarian development, oocyte maturation, fertilization competence, and the potential of a fertilized egg for implantation and development (Yatsenko and Rajkovic, 2019). Ovulation disorders are the leading cause of female infertility, which often occur as a result of conditions classified into three categories: hypothalamic failure, dysfunction of hypothalamic-pituitary-ovarian axis-mostly polycystic ovary syndrome (PCOS), and primary ovarian insufficiency (POI) (National Institute for Health and Care Excellence, 2013). Genetic factors are suggested to play a role in all these disorders. For example, mutations of the GNRHR gene encoding the gonadotropin-releasing hormone (GnRH) receptor and genes causing Kallmann syndrome have been identified in women affected by hypothalamic amenorrhea. Alternations in multiple genes such as CYP17, CYP19, LHCGR, DENND1A are linked to PCOS, suggesting its polygenicity (reviewed in Beke, 2019).

POI has become a significant cause of female infertility due to premature exhaustion of the primordial follicular pool in most cases (Rossetti et al., 2017). The most common contributors to POI are the X chromosome-linked defects, in which Turner syndrome (TS) is the primary cause of syndromic POI. In contrast, premutation of the FMR1 (fragile X mental retardation 1) gene is the most common gene mutation associated with non-syndromic POI. In most cases of POI, the activation of the surveillance mechanisms leading to a reduced ovarian reserve are responsible for infertility. For instance, the absence of one X chromosome in TS causes oocyte loss during early meiotic prophase and ovarian development, leading to ovarian dysgenesis and primary amenorrhea since infancy (Fechner et al., 2006). In other cases, how particular mutations (e.g., FMR1 premutation) lead to POI is not clear yet. The FMR1 premutation may cause a deficiency of proteins required for oocyte or follicle development and survival (Rossetti et al., 2017). Even though nowadays POI cannot be reverted, the identification of the causative genetic alterations in POI patients is beneficial for her female relatives, who can undertake precautionary measures (e.g., egg freezing, embryo cryopreservation, anticipated pregnancy planning, *etc.*) in case of being positive in the genetic screening (Rossetti et al., 2017). This perspective is becoming increasingly important due to the modern tendency to delay childbirth in societies.

Despite the revealed genetic factors contributing to female and male infertility, many genetic causes remain unexplained for the majority of infertility cases, including idiopathic infertility cases, which are identified in 25%–30% of infertility couples and likely have a genetic etiology (Smith et al., 2003; Mallepaly et al., 2017). Furthermore, with the increasing use of assisted reproductive technology (ART), which removes the natural barrier to egg fertilization, concerns about its safety and possible adverse outcomes are rising (Davies et al., 2012). Diagnosing the genetic causes of infertility becomes more clinically significant for infertility treatment and the health of patients and their children. Thus, identifying unknown genes involved in mammalian gametogenesis, which could contribute to human infertility, is demanding and essential for clinical infertility diagnosis in the near future.

### 3.1 Mutations of meiotic prophase genes in mice

In recent years, advances in genomic approaches, particularly next-generation sequencing (NGS) technologies, allowed unbiased genomic studies of human infertility and uncovered many infertility-associated genes or gene variants in males and females (Yatsenko and Rajkovic, 2019; Precone et al., 2021; Heddar et al., 2022). Advanced filtering techniques are required for selecting the *bona fide* causes of human infertility from the discovered genes or gene variants, and mouse studies are the gold standard for defining the genotype-phenotype connection in fertility, at least in males (Houston et al., 2021). Moreover, functional studies in mouse models are usually prerequisites to attributing a disease-causing role to a newly discovered gene (Riera-Escamilla et al., 2019), thus offering a panel of strong candidate genes for screening human infertility factors.

Here, we summarized genes that are functionally involved in meiotic prophase I, and mutating any of them could trigger recombination/synapsis checkpoint, leading to spermatocyte arrest in males and/or oocyte depletion in females (Supplementary Table S1).

Of these 77 genes, many of them have essential roles in chromosome pairing, synapsis, and meiotic recombination (detailed roles are discussed above), including components of the chromosome axis or the SC, recombination factors required for DSB formation and repair, or proteins participating in telomere-mediated chromosome movements. The rest are mainly functionally related to silencing retrotransposons, chromatin modification, and transcriptional and translational regulation of essential proteins required for SC formation and DSB repair.

Intriguingly, more than half of these meiosis-deficient mutants display sexually dimorphic phenotypes. Less stringent checkpoint controls in females could explain these phenotypic differences. Consequently, oocytes could tolerate more meiotic prophase I error, which would explain why oogenesis is more error-prone than spermatogenesis (Hunt and Hassold, 2002). In male mice deficient for *Brca2*, *Mei1*, *Hormad1*, *Smc1b*, or *Sycp3* genes, spermatocytes are arrested at the pachytene stage due to the defective meiotic prophase events, resulting in male sterility (Supplementary Table S1). However, these mutant oocytes are only partially arrested at meiotic prophase I in females, and some progress beyond prophase I despite carrying asynaptic homologs, unrepaired DSBs, or other chromosomal abnormalities. Other mechanisms during oogenesis can eliminate these defective oocytes later, but some even complete meiosis and form unbalanced oocytes. As a result, some of these meiosis-deficient mutant females are even subfertile (Reinholdt and Schimenti, 2005; Daniel et al., 2011; Felipe-Medina et al., 2020).

Another explanation could be that some of these genes have sexually dimorphic roles. For example, *Hells* and *Rad21l* genes have distinct roles in males and females. While the deficient males are infertile due to meiotic prophase I arrest, the mutant females exhibit lethality (*Hells*), or subfertility (*Rad21l*), due to other defects rather than failed synapsis or incomplete meiotic recombination. On the other hand, *Asz1*, *Dnmt3l*, *Mybl1*, *Mov10l1*, *Piwil2*, *Piwil4*, *Pld6*, and *Tdrd9* are specifically required for the silencing of retrotransposons in males, while *Dmrt7* has significant roles in meiotic silencing of the XY chromosomes which only exist in spermatocytes. Thus, the disruption of these genes causes male infertility due to a complete arrest in spermatocytes, but female fertility is grossly unaffected (Supplementary Table S1).

Furthermore, some recombination factors, such as BRCA1, BRME1, MEILB2, and TEX15, recruit recombinases RAD51/DMC1 to DSB sites in spermatocytes. The mutations of these genes result in male infertility but only have mild or no effects on female fertility (Supplementary Table S1). While if they have similar roles in females is unclear or cannot be excluded, the milder phenotypes in female mutant mice might be a consequence of combined effects from the weak checkpoint control and less-required roles during meiotic recombination in oocytes.

In humans, meiotic defects typically result in non-obstructive azoospermia (NOA), whereas in females, they are usually associated with POI (Krausz et al., 2020). We searched these 77 meiotic prophase genes, the mutation of which could trigger meiotic prophase arrest in mice, in ClinVar and Pubmed, and found monogenetic mutations of 28 genes (145 variants) have been reported to be associated with human infertility conditions such as spermatogenetic failure, NOA, POI, spermatogenesis maturation arrest, pregnancy loss, *etc.* (Supplementary Table S2).

Based on the interpretations for clinical significance in ClinVar, 55 of these variants are considered as “likely pathogenic” or “uncertain significance” (Supplementary Table S2). This is mainly due to the lack of evidence or inconsistent interpretations. Of the 145 variants, 67 variants from 21 genes are classified as ‘pathogenic’, 52 variants from 16 genes have only 1 or 2 publications reporting independent probands, and single submitters provide the remainder without publications. Moreover, only five variants from five genes (*Six6os1*, *Meilb2*, *Msh5*, *Stag3*, and *Syce1*) have supporting biological evidence from knockout mouse models in which human phenotypes are recapitulated (Supplementary Table S2). Thus, in the future, more independent validation studies and functional evidence are required, including introducing gene variants using CRISPR/Cas9 genome editing technology in mice to validate the infertility-causing roles of human gene variants (Houston et al., 2021), not only for distinguishing between variants that cause disease from variants that are rare but benign (Araujo et al., 2020), but also for providing robustness to the clinical validity of these possible disease-causing genes linked to human infertility (Oud et al., 2019).

Additionally, multiple levels of evidence should also be considered to confidently link variation in individual genes to human infertility (Oud et al., 2019). Indeed, an unstructured assessment has reported three genes, including *Tex11*, that fulfill this requirement for a link to male infertility (Tüttelmann et al., 2018). Recently, in another study of an extensive literature review and standardized clinical validity assessment of a large number of genes, some of these meiotic prophase genes were shown to be associated with male infertility with ‘strong’ evidence (*Tex11* and *Tex15*), with ‘moderate’ evidence (*Sycp*3), or with ‘limited’ evidence (*Dmc1*, *Mei1*, *Meiob*, *Spo11*, *Syce1*, and *Tdrd9*) (Oud et al., 2019).

Importantly, the associated conditions of all these 28 genes in humans are well matched with the phenotypes of their mutant mice (Supplementary Table S2). For example, the mutation of SYCP3 causes male infertility with complete meiotic prophase arrest. Still, it exhibits subfertility in female mice with a sharp reduction in litter size due to the presence of aneuploid oocytes. Correspondingly, its linked conditions in humans are infertility/spermatogenetic failure in men and pregnancy loss in women. This further support the values of mouse models for attributing a disease-causing role to a new gene. Thus, the remaining meiotic prophase genes with no monogenic mutation identified in this list are worthy of screening in human patients.

However, it is essential to point out that we must be cautious when using the findings from mouse studies to interpret the causative factors and mechanisms underlying human infertility regarding considerable differences still exist between humans and mice (Azhar et al., 2021). A recent study has shown that the metaphase checkpoint is more frequently activated than the pachytene checkpoint in human males with severe spermatogenic impairment (Enguita-Marruedo et al., 2019), which is in contrast to observations in the mouse, where knockout of the meiotic prophase genes (as we summarized above) most frequently results in pachytene checkpoint arrest. The underlying reasons are not clear. It could be that the observed arrest in this study is caused mainly by mutations in proteins required for the metaphase-anaphase transition or functioning in cell cycle regulation rather than involved in meiotic prophase major events (Enguita-Marruedo et al., 2019). Alternatively, mutation of meiotic prophase genes may trigger a later metaphase arrest in humans rather than prophase arrest in mice. Differences in the pachytene surveillance mechanisms between humans and mice could cause this. Most studies (58 out of 78 publications in Supplementary Table S1) reporting mutations of meiotic prophase genes in infertile males lack detailed analysis of meiotic or testicular phenotypes. Thus, to clarify this possibility, it will be worthwhile to assess the exact spermatocyte arrest phase in infertile patients carrying meiotic prophase gene mutations in the future.
