# Supplementary material for: Genetic control of meiosis surveillance mechanisms in mammals
Source: Front Cell Dev Biol. 2023 Feb 23;11:1127440. doi: 10.3389/fcell.2023.1127440 (PMC9996228; doi:10.3389/fcell.2023.1127440)
Supplement: Supplementary file 1 [file DataSheet2.PDF]

Table S2 Meiotic essential genes that have variants identified in human patients.

Gene ID	Symbol	Variation Human	Conditions	Meiotic/testicular phenotype	Clinical significance	PMID/ClinVar accession	Comment
625662	Ankrd31	NM_001372053.1(ANKRD31):c.1565-2A>G	Genetic non-acquired premature ovarian failure		Pathogenic	VCV001120014.1	
		NM_001372053.1(ANKRD31):c.985C>T (p.Gln329Ter)	Genetic non-acquired premature ovarian failure		Pathogenic	VCV001120015.1	
13164	Dazl	NM_001351.4(DAZL):c.160A>G (p.Thr54Ala)	Spermatogenic failure, susceptibility to		risk factor	12414900	Varied phenotypes in human patients without clear MP1 arrest.
13404	Dmc1	NM_007068.4(DMC1):c.860C>A (p.Pro287His)	Azoospermia		Pathogenic	VCV001328945.1	
		NM_007068.4(DMC1):c.598A>G (p.Met200Val)	Premature ovarian failure		Benign	18166824	
		NM_007068.4(DMC1):c.364A>G (p.Thr122Ala)	Azoospermia		Pathogenic	VCV001328944.1	
19183	Hop2	NM_016556.4(PSMC3IP):c.338-15C>G	Ovarian dysgenesis 3		Benign	VCV001285798	
		NM_016556.4(PSMC3IP):c.-35C>T	Ovarian dysgenesis 3		Uncertain significance	VCV000801407	
		NM_016556.2(PSMC3IP): c.600_602del (p.Glu201del)	Ovarian dysgenesis 3		/	21963259	
384619	Kash5	NM_144688.5(KASH5):c.747G>A (p.Ala249=)	Genetic non-acquired premature ovarian failure		Likely pathogenic	VCV001232307	
		NM_144688.5(KASH5):c.1146+5G>A	Azoospermia		Pathogenic	VCV001328949	
		seq [GRCh37] del(19) (19q13.33) chr19: g.49894043-49903011del	non-obstructive azoospermia	Arrest at zygotene-like stage with a deficiency in homolog pairing and synapsis	/	35674372	From abstract of this paper
		NM_144688: c.979_980del: p. R327Sfs*21			/		
110958	M1ap	NM_001321739.2(M1AP):c.1435-1G>A	Spermatogenic failure 48, Spermatogenesis maturation arrest		Pathogenic	32017041	

Table S2 Meiotic essential genes that have variants identified in human patients.

		NM_001321739.2(M1AP):c.1289T>C (p.Leu430Pro)	Non-obstructive azoospermia		Uncertain significance	VCV000805832.2	
		NM_001321739.2(M1AP):c.1166C>T (p.Pro389Leu)	Spermatogenic failure 48, non-obstructive azoospermia	Maturation arrest at round spermatid stage	Uncertain significance	32673564	
		NM_001321739.2(M1AP):c.949G>A (p.Gly317Arg)	Spermatogenic failure 48, non-obstructive azoospermia	Maturation arrest at round spermatid stage	Uncertain significance	32673564	
		NM_001321739.2(M1AP):c.797G>A (p.Arg266Gln)	Spermatogenic failure 48, Cryptozoospermia, Non-obstructive azoospermia	Predominant meiotic arrest with occasional postmeiotic germ cells	Uncertain significance	32673564	
		NM_001321739.2(M1AP):c.676dup (p.Trp226fs)	Spermatogenic failure 48, non-obstructive azoospermia	Meiotic arrest at spermatocyte stage	Pathogenic	32673564	
		NM_001321739.2(M1AP):c.148T>C (p.Ser50Pro)	Non-obstructive azoospermia		Uncertain significance	VCV000805833.2	
74377	Meilb2*	NM_007031.2(HSF2BP):c.557T>C (p.Leu186Pro)	Premature ovarian failure 19		Likely pathogenic	VCV001224546.1	
		NM_007031.2(HSF2BP):c.500C>T (p.Ser167Leu)	Premature ovarian failure 19		Pathogenic	32845237	
		NM_007031.2(HSF2BP):c.382T>C (p.Cys128Arg)	Premature ovarian failure 19		Likely pathogenic	VCV001224547.1	
75178	Meiob	NM_001163560.3(MEIOB):c.191A>T (p.Asn64Ile)	Spermatogenic failure 22		Pathogenic	28206990	
76915	Mnd1	GRCh37/hg19 4q31.3(chr4:154316483-154325120)	Premature ovarian failure		Likely pathogenic	31042289	
83456	Mov10l1	NM_018995.3(MOV10L1):c.743+5G>A	Azoospermia		Pathogenic	VCV001328947.1	
		NM_018995.3(MOV10L1):c.2447G>T (p.Ser816Ile)	Spermatogenic failure 73		Pathogenic	35476666	
		NM_018995.3(MOV10L1):c.2542G>A (p.Gly848Arg)	Spermatogenic failure 73		Pathogenic	35476666	
55993	Msh4	NM_002440.4(MSH4):c.1025C>T (p.Thr342Ile)	Genetic non-acquired premature ovarian failure		Likely pathogenic	VCV001256043	

Table S2 Meiotic essential genes that have variants identified in human patients.

		NM_002440.4(MSH4):c.1063A>G (p.Ile355Val)	Genetic non-acquired premature ovarian failure		Likely pathogenic	VCV001255997	
		NM_002440.4(MSH4):c.1453C>T (p.Gln485Ter)	Non-obstructive azoospermia		Likely pathogenic	VCV000992887	
		NM_002440.4(MSH4):c.1686del (p.Lys562_Val563insTer)	Non-obstructive azoospermia		Likely pathogenic	VCV000992888	
		NM_002440.4(MSH4):c.1855A>G (p.Met619Val)	Genetic non-acquired premature ovarian failure		Likely pathogenic	VCV001256012	
		NM_002440.4(MSH4):c.2198C>A (p.Ser733Ter)	Non-obstructive azoospermia		Likely pathogenic	VCV000992889	
		NM_002440.4(MSH4):c.2222_2225del (p.Lys741fs)	Genetic non-acquired premature ovarian failure		Pathogenic	VCV001256044	
		NM_002440.4(MSH4):c.2261C>T (p.Ser754Leu)	Oligospermia|Premature ovarian insufficiency|non-obstructive azoospermia		Pathogenic/Likely pathogenic	33448284	
		NM_002440.4(MSH4):c.2374A>G (p.Thr792Ala)	Genetic non-acquired premature ovarian failure		Likely pathogenic	VCV001256001	
		NM_002440.4(MSH4):c.2728C>T (p.Arg910Ter)	Genetic non-acquired premature ovarian failure		Pathogenic	VCV001256045	
17687	Msh5*	NM_172166.4(MSH5):c.75dup (p.Ser26fs)	Non-obstructive azoospermia		Pathogenic	34755185	
		NM_172166.4(MSH5):c.826C>T (p.Arg276Cys)	Genetic non-acquired premature ovarian failure		Likely pathogenic	VCV001255996	
		NM_172166.4(MSH5):c.964C>T (p.Arg322Cys)	Non-obstructive azoospermia		Likely pathogenic	34755185	
		NM_172166.4(MSH5):c.1051C>G (p.Arg351Gly)	Genetic non-acquired premature ovarian failure		Pathogenic	VCV001256029	
		NM_172166.4(MSH5):c.1459G>T (p.Asp487Tyr)	Premature ovarian failure 13, nonobstructive azoospermia		Pathogenic	28175301; 34980881	
		NM_172166.4(MSH5):c.1857del (p.Ala620fs)	Non-obstructive azoospermia		Pathogenic	34755185	
3673	Rec114	NM_001042367.2(REC114):c.397T>G (p.Cys133Gly)	Oocyte maturation defect 10		Pathogenic	31704776	
		NM_001042367.2(REC114):c.546+5G>A	Oocyte maturation defect 10		Pathogenic	31704776	

Table S2 Meiotic essential genes that have variants identified in human patients.

56739	Rec8	NM_001048205.2(REC8):c.91C>T (p.Arg31Cys)	Non-obstructive azoospermia		Likely pathogenic	31479588	
		NM_001048205.2(REC8):c.624+1G>A	Premature ovarian insufficiency		Likely pathogenic	VCV001214014	
		NM_001048205.2(REC8):c.872C>T (p.Pro291Leu)	Premature ovarian insufficiency		Uncertain significance	VCV000619070	
		NM_001048205.2(REC8):c.1035_1036dup (p.Ala346fs)	Premature ovarian insufficiency		Likely pathogenic	VCV001214013	
		NM_001048205.2(REC8):c.1057A>C (p.Thr353Pro)	Premature ovarian failure		Uncertain significance	VCV000929773	
100155	shoc1	NM_001378211.1(SHOC1):c.1277_1278del (p.Glu426fs)	Non-obstructive azoospermia		Pathogenic	VCV001244232	
75801	Six6os1*	NM_174978.3(C14orf39):c.1180-3C>G	Spermatogenic failure 52, non-obstructive azoospermia	Incomplete synapsis, meiotic arrest the pachytene-like stage	Pathogenic	33508233	
		NM_174978.3(C14orf39):c.958G>T (p.Glu320Ter)	Spermatogenic failure 52, non-obstructive azoospermia	Incomplete synapsis, meiotic arrest at spermatocyte stage	Pathogenic	33508233	
		NM_174978.3(C14orf39):c.204_205del (p.His68fs)	Azoospermia, Non-obstructive azoospermia, Spermatogenic failure 52, Premature ovarian failure 18	Complete asynapsis between homologs, meiotic arrest at the pachytene-like stage	Pathogenic	33508233	
140557	Smc1b	NM_148674.5(SMC1B):c.863A>G (p.Glu288Gly)	Genetic non-acquired premature ovarian failure		Likely pathogenic	VCV001256022	
26972	Spo11	NM_012444.3(SPO11):c.744G>A (p.Thr248=)	Non-obstructive azoospermia		Likely pathogenic	VCV001244233.1	
50878	Stag3*	NM_001282717.2(STAG3):c.48G>T (p.Leu16Phe)	Spermatogenic failure 61|Premature ovarian failure 8		Benign	VCV001236675	

Table S2 Meiotic essential genes that have variants identified in human patients.

<table>
<tr><td></td><td></td><td>NM_001282717.2(STAG3):c.106A>C (p.Thr36Pro)</td><td>Spermatogenic failure 61|Premature ovarian failure 8</td><td></td><td>Benign</td><td>VCV001183740</td><td></td></tr>
<tr><td></td><td></td><td>NM_001282717.2(STAG3):c.291dup (p.Asn98fs)</td><td>Premature ovarian failure 8</td><td></td><td>Pathogenic</td><td>30006057</td><td>Heterozygous pathogenic variant</td></tr>
<tr><td></td><td></td><td>NM_001282717.2(STAG3):c.562del (p.Gln188fs)</td><td>Premature ovarian failure 8</td><td></td><td>Pathogenic</td><td>24597867</td><td></td></tr>
<tr><td></td><td></td><td>NM_001282717.2(STAG3):c.962G>A (p.Arg321His)</td><td colspan="2">Premature ovarian failure 8|Spermatogenic failure 61|PRIMARY OVARIAN FAILURE 8</td><td>Pathogenic/Likely pathogenic</td><td>32634216</td><td></td></tr>
<tr><td></td><td></td><td>NM_001282717.2(STAG3):c.1069C>T (p.Arg357Ter)</td><td>Premature ovarian failure</td><td></td><td>Pathogenic</td><td>VCV000929755</td><td></td></tr>
<tr><td></td><td></td><td>NM_001282717.2(STAG3):c.1245-26T>C</td><td>Spermatogenic failure 61|Premature ovarian failure 8|not provided</td><td></td><td>Benign</td><td>VCV001229759</td><td></td></tr>
<tr><td></td><td></td><td>NM_001282717.2(STAG3):c.1262T>G (p.Leu421Arg)</td><td>Spermatogenesis maturation arrest|non-obstructive azoospermia|Spermatogenic failure 61</td><td>Deficient chromosomal axis and SC formation</td><td>Likely pathogenic</td><td>31682730</td><td>Heterozygous pathogenic variant</td></tr>
<tr><td></td><td></td><td>NM_001282717.2(STAG3):c.1293A>C (p.Pro431=)</td><td>Spermatogenic failure 61|Premature ovarian failure 8|not provided</td><td></td><td>Benign</td><td>VCV001243986</td><td></td></tr>
<tr><td></td><td></td><td>NM_001282717.2(STAG3):c.1312C>T (p.Arg438Ter)</td><td>Spermatogenesis maturation arrest|non-obstructive azoospermia|Spermatogenic failure 61</td><td>Deficient chromosomal axis and SC formation</td><td>Likely pathogenic</td><td>31682730</td><td>Heterozygous pathogenic variant</td></tr>
<tr><td></td><td></td><td>NM_001282717.2(STAG3):c.1571del (p.Gln524fs)</td><td>Premature ovarian failure 8</td><td></td><td>Pathogenic</td><td>VCV000869148</td><td></td></tr>
<tr><td></td><td></td><td>NM_001282717.2(STAG3):c.1573+5G>A</td><td>Premature ovarian failure 8</td><td></td><td>Pathogenic</td><td>28393351</td><td></td></tr>
<tr><td></td><td></td><td>NM_001282717.2(STAG3):c.1573+41C>G</td><td>Spermatogenic failure 61|Premature ovarian failure 8|not provided</td><td></td><td>Benign</td><td>VCV001287786</td><td></td></tr>
</table>

Table S2 Meiotic essential genes that have variants identified in human patients.

<table>
<tr><td rowspan="12"></td><td rowspan="12"></td><td>NM_001282717.2(STAG3):c.1936dup (p.Ala646fs)</td><td>Spermatogenic failure 61</td><td rowspan="2">Persistence of meiotic DSBs and a failure to complete chromosome pairing</td><td>Pathogenic</td><td>31125047</td><td rowspan="2">Compound heterozygosis, heterozygous for the first variant and homozygous for the second variant</td></tr>
<tr><td>NM_001282717.2(STAG3):c.2394+1G>A</td><td>Spermatogenic failure 61</td><td>Pathogenic</td><td>31125047</td></tr>
<tr><td>NM_001282717.2(STAG3):c.1942G>A (p.Ala648Thr)</td><td>Non-obstructive azoospermia|Premature ovarian insufficiency</td><td></td><td>Uncertain significance</td><td>35176428</td><td rowspan="2">Double homozygous for both variants</td></tr>
<tr><td>NM_001282717.2(STAG3):c.1953_1955del (p.Leu652del)</td><td>Non-obstructive azoospermia|Premature ovarian insufficiency</td><td></td><td>Likely pathogenic</td><td>35176428</td></tr>
<tr><td>NM_001282717.2(STAG3):c.1947_1948dup (p.Tyr650fs)</td><td>Premature ovarian failure 8</td><td></td><td>Pathogenic</td><td>26059840</td><td></td></tr>
<tr><td>NM_001282717.2(STAG3):c.1950C>A (p.Tyr650Ter)</td><td>Premature ovarian failure 8</td><td></td><td>Pathogenic</td><td>30006057</td><td>Heterozygous pathogenic variant</td></tr>
<tr><td>NM_001282717.2(STAG3):c.2445T>A (p.Ile815=)</td><td>Spermatogenic failure 61|Premature ovarian failure 8|not provided</td><td></td><td>Benign</td><td>VCV001230818</td><td></td></tr>
<tr><td>NM_001282717.2(STAG3):c.2627G>A (p.Gly876Glu)</td><td>Premature ovarian failure 8</td><td></td><td>Uncertain significance</td><td>VCV001214010</td><td></td></tr>
<tr><td>NM_001282717.2(STAG3):c.2776C>T (p.Arg926Ter)</td><td colspan="2">not provided|Premature ovarian insufficiency|Abnormality of the ovary|Premature ovarian insufficiency|Female infertility|Premature ovarian failure 8</td><td>Likely pathogenic</td><td>VCV000374000</td><td></td></tr>
<tr><td>NM_001282717.2(STAG3):c.3381_3384del (p.Glu1128fs)</td><td>PRIMARY OVARIAN FAILURE 8</td><td></td><td>Pathogenic</td><td>34828315</td><td></td></tr>
<tr><td>74075</td><td>Syce1*</td><td>NM_001143764.3(SYCE1):c.721C>T (p.Gln241Ter)</td><td>Premature ovarian failure 12</td><td></td><td>Pathogenic</td><td>25062452; 32917591</td><td></td></tr>
<tr><td></td><td></td><td>NM_001143764.3(SYCE1):c.197-2A>G</td><td>Spermatogenic failure 15</td><td></td><td>Pathogenic</td><td>25899990</td><td></td></tr>
</table>

Table S2 Meiotic essential genes that have variants identified in human patients.

320558	Sycp2	NM_014258.4(SYCP2):c.3067_3071del (p.Lys1023fs)	Oligosynaptic infertility, Early spermatogenesis maturation arrest, non-obstructive azoospermia	Meiotic arrest at the pachytene spermatocyte stage	Pathogenic/Likely pathogenic	31866047	Heterozygous pathogenic variant
		NM_014258.4(SYCP2):c.2793_2797del (p.Lys932fs)	Cryptozoospermia, Oligosynaptic infertility, non-obstructive azoospermia		Pathogenic/Likely pathogenic	31866047	Heterozygous pathogenic variant
		NM_014258.4(SYCP2):c.2022_2025del (p.Lys674fs)	Cryptozoospermia, Oligosynaptic infertility, non-obstructive azoospermia		Pathogenic/Likely pathogenic	31866047	Heterozygous pathogenic variant
20962	Sycp3	NM_001177949.2(SYCP3):c.657T>C (p.Thr219=)	PREGNANCY LOSS 4		Pathogenic	19110213	Heterozygous pathogenic variant
		NM_001177949.2(SYCP3):c.553-21_553-18del	PREGNANCY LOSS 4		Pathogenic	19110213	Heterozygous pathogenic variant
		NM_001177949.2(SYCP3):c.524_527del (p.Ile175fs)	Spermatogenic failure 4		Uncertain significance	29713536; 28801929	
		NM_001177949.2(SYCP3):c.454-13_454-9del	Spermatogenic Failure		Benign	VCV000306763	
		NM_001177949.2(SYCP3):c.435A>G (p.Glu145=)	Spermatogenic failure 4		Benign	VCV000306764	
		NM_001177949.2(SYCP3):c.241A>C (p.Ile81Leu)	Male infertility		Uncertain significance	VCV000869113	
		NM_001177949.2(SYCP3):c.80T>C (p.Phe27Ser)	Spermatogenic failure 4		Likely benign	VCV000880979	
		NM_001177949.2(SYCP3):c.59A>G (p.Gln20Arg)	Spermatogenic failure 4		Benign	VCV000880980	
		NM_001177949.2(SYCP3):c.28A>T (p.Arg10Trp)	Spermatogenic failure 4		Uncertain significance	VCV000880981	
		NM_001177949.2(SYCP3):c.-53C>G	Spermatogenic failure 4		Benign	VCV000880982	
		NM_001177949.2(SYCP3):c.-64C>T	Spermatogenic failure 4		Uncertain significance	VCV000306765	

Table S2 Meiotic essential genes that have variants identified in human patients.

		NM_001177949.2(SYCP3):c.-74T>C	Spermatogenic failure 4		Uncertain significance	VCV000882343	
		NM_001177949.2(SYCP3):c.-106A>G	Spermatogenic failure 4		Benign	VCV000882344	
		NM_001177949.2(SYCP3):c.-122T>A	Spermatogenic failure 4		Benign	VCV000306766	
		SYCP3, 1-BP DEL, 643A	Spermatogenic failure 4		Pathogenic	14643120	
74691	Tdrd9	NM_153046.3(TDRD9):c.46A>C (p.Ile16Leu)	Spermatogenic failure 30		Uncertain significance	VCV001027806	
		NM_153046.3(TDRD9):c.448G>A (p.Val150Met)	Spermatogenic failure 30		Uncertain significance	VCV001027805	
		NM_153046.3(TDRD9):c.720_723del (p.Ser241fs)	Azoospermia|Spermatogenic failure 30		Pathogenic	28536242	
		NM_153046.3(TDRD9):c.2106+2T>A	Spermatogenic failure 30		Uncertain significance	VCV001301818	
		NM_153046.3(TDRD9):c.3483_3484dup (p.Ser1162fs)	Azoospermia		Pathogenic	VCV001328950	
320022	Terb1	NM_001136505.2(TERB1):c.1813C>T (p.Arg605Ter)	Spermatogenic failure 60	Aberrant γH2AX pattern	Pathogenic	32741963	Double homozygous for both variants
		NM_001136505.2(TERB1):c.289_290del (p.Leu97fs)	Spermatogenic failure 60		Pathogenic	32741963	
		NM_001136505.2(TERB1):c.1703C>G (p.Ser568Ter)	Non-obstructive azoospermia|Spermatogenic failure 60	Arrest at spermatocyte stage	Pathogenic	33211200	
		NM_001136505.2(TERB1):c.733G>A (p.Gly245Arg)	Azoospermia		Pathogenic	VCV001328957	
74401	Terb2	NM_152448.3(TERB2):c.434G>A (p.Ser145Asn)	Non-obstructive azoospermia		Likely pathogenic	VCV001244236	
		NM_152448.3(TERB2):c.457_458del (p.Thr153fs)	Spermatogenic failure 59		Pathogenic	33211200	
		NM_152448.3(TERB2):c.544dup (p.Met182fs)	Spermatogenic failure 59		Pathogenic	33211200	
83558	Tex11	NM_031276.3(TEX11):c.2568G>T (p.Trp856Cys)	Non-obstructive azoospermia		Pathogenic	25970010	
		NM_031276.3(TEX11):c.2047G>A (p.Ala683Thr)	Spermatogenic failure, X-linked, 2|not specified	Partial meiotic arrest with very few postmeiotic cells detected.	Uncertain significance	25970010	

Table S2 Meiotic essential genes that have variants identified in human patients.

		NM_031276.3(TEX11):c.1751+2T>G	Non-obstructive azoospermia		Pathogenic	25970010	
		NM_031276.3(TEX11):c.1381-1G>A	Non-obstructive azoospermia		Pathogenic	25970010	
		NM_031276.3(TEX11):c.1208dup (p.Asn403fs)	Non-obstructive azoospermia		Pathogenic	25970010	
		NM_031276.3(TEX11):c.1006G>T (p.Glu336Ter)	Non-obstructive azoospermia		Pathogenic	25970010	
		NM_031276.3(TEX11):c.812del (p.Lys271fs)	Non-obstructive azoospermia		Pathogenic	25970010	
		NM_031276.3(TEX11):c.466A>G (p.Met156Val)	not specified|Spermatogenic failure, X-linked, 2	Meiotic arrest at spermatocyte stage	Uncertain significance	25970010	
		NM_031276.3(TEX11):c.405C>T (p.Ala135=)	Spermatogenic failure, X-linked, 2|not provided		Benign	25970010	
		NM_031276.3(TEX11):c.253del (p.Val85fs)	Non-obstructive azoospermia		Pathogenic	25970010	
		NC_000023.10:g.69954448_70045530del	Spermatogenic failure, X-linked, 2	Mixed testicular atrophy with meiotic arrest	Pathogenic	25970010	
104271	Tex15	NM_001350162.2(TEX15):c.9448C>T (p.Arg3150Ter)	Non-obstructive azoospermia		Uncertain significance	VCV001244246	
		NM_001350162.2(TEX15):c.9223G>A (p.Gly3075Arg)	not provided|non-obstructive azoospermia		Conflicting interpretations of pathogenicity	31479588	
		NM_001350162.1:c.9223G>A(;)7118G>A	Non-obstructive azoospermia		Uncertain significance	VCV000684732	
		NM_001350162.2(TEX15):c.8197_8198del (p.Glu2733fs)	Non-obstructive azoospermia		Pathogenic	VCV001244247	
		NM_001350162.2(TEX15):c.8083C>T (p.Arg2695Ter)	Spermatogenic failure 25		Pathogenic	28303806	
		NM_001350162.2(TEX15):c.7777A>G (p.Thr2593Ala)	Non-obstructive azoospermia		Uncertain significance	VCV001244248	
		NM_001350162.2(TEX15):c.7118G>A (p.Ser2373Asn)	Non-obstructive azoospermia		Likely benign	31479588	

Table S2 Meiotic essential genes that have variants identified in human patients.

		NM_001350162.2(TEX15):c.5170G>A (p.Ala1724Thr)	Non-obstructive azoospermia		Uncertain significance	VCV001285393	
		NM_001350162.2(TEX15):c.4189del (p.Ser1397fs)	Spermatogenic failure 25		Pathogenic	28355598	
		NM_001350162.2(TEX15):c.3568A>T (p.Lys1190Ter)	Spermatogenic failure 25		Pathogenic	28355598	
		NM_001350162.2(TEX15):c.3323T>C (p.Leu1108Pro)	Spermatogenic failure 25		Uncertain significance	VCV001030942	
		NM_001350162.2(TEX15):c.3279T>G (p.Tyr1093Ter)	Spermatogenic failure 25|Oligosynaptic infertility	Arrest at the primary spermatocyte stage	Pathogenic	26199321	
		NM_001350162.2(TEX15):c.1261G>A (p.Gly421Ser)	Spermatogenic failure 25		Uncertain significance	VCV001339092	
69716	Trip13	NM_004237.4(TRIP13):c.77A>G (p.His26Arg)	Oocyte maturation defect 9		Pathogenic	32473092	
		NM_004237.4(TRIP13):c.518G>A (p.Arg173Gln)	Oocyte maturation defect 9		Pathogenic	32473092	
		NM_004237.4(TRIP13):c.592A>G (p.Ile198Val)	Oocyte maturation defect 9		Pathogenic	32473092	
		NM_004237.4(TRIP13):c.608+39T>G	not provided|Mosaic variegated aneuploidy syndrome 3|Oocyte maturation defect 9		Benign	VCV001241500	
		NM_004237.4(TRIP13):c.673-1G>C	Mosaic variegated aneuploidy syndrome 3		Pathogenic	28553959	
		NM_004237.4(TRIP13):c.712G>A (p.Asp238Asn)	Mosaic variegated aneuploidy syndrome 3		Uncertain significance	VCV001031392	
		NM_004237.4(TRIP13):c.739G>A (p.Val247Met)	Oocyte maturation defect 9		Pathogenic	32473092	
		NM_004237.4(TRIP13):c.907G>A (p.Glu303Lys)	Oocyte maturation defect 9		Pathogenic	32473092	
		NM_004237.4(TRIP13):c.1060C>T (p.Arg354Ter)	Mosaic variegated aneuploidy syndrome 3		Pathogenic	28553959	
