# Supplementary material for: Genetic control of meiosis surveillance mechanisms in mammals
Source: Front Cell Dev Biol. 2023 Feb 23;11:1127440. doi: 10.3389/fcell.2023.1127440 (PMC9996228; doi:10.3389/fcell.2023.1127440)
Supplement: Supplementary file 2 [file Table1.docx]

| **Gene ID** | **Symbol** | **Function in meiotic prophase I** | **Function category** | **Phenotypes in male mutants** | | **Phenotypes in female mutants** | | **PMID** | **Comments** |
| --- | --- | --- | --- | --- | --- | --- | --- | --- | --- |
| 625662 | **Ankrd31** | Regulates DSB formation by stabilizing REC114 and other factors on axes | Programmed DSB formation | Partial pachytene arrest, sterile | Defective synapsis, delayed early recombination process, failed CO formation between XY chromosomes | Reduced oocyte reserve, premature ovarian failure | Defective synapsis, delayed early recombination process | 31003867; 31000436 |  |
| 74068 | Asz1 | A structural role of nuage; regulates epigenetic and post-transcriptional silencing of retrotransposons by stabilizing MILI in nuage | Silencing of retrotransposon | Complete arrest at zygotene-pachytene stage, sterile | Absence of sex body, loss of MILI | Normal fertility |  | 19730684 |  |
| 245000 | Atr | Promotes the loading of strand-exchange proteins at DSBs and accumulation of recombination markers on the axes of unsynapsed chromosomes; required for correct timing of crossover formation and elongation of the synaptonemal complex | Meiotic recombination; SC formation | Arrest at epithelial stage IV/mid-pachytene stage | Asynapsis , reduced RPA, RAD51 and DMC1 foci during early recombination, elevated RNF212 foci at pachynema | *N.A.* |  | 29977027; 29976923 | Germline-specific deletion |
| 11920 | Atm | DNA-damage checkpoint protein in response to SPO11-induced DSB, supressing DSB formation via a negative feedback loop | Programmed DSB formation; DSB sensing | Complete arrest at early pachytene | Defective synapsis, abnormal assembly of ATR, DMC1 and RAD51, reduced γH2AX signals from leptotene to zygotene, rarely found sex body | Complete arrest at dictyate stage, oocyte depletion by 4 dpp, infertile | | 9735362; 16055729; 15640358 |  |
| 12189 | Brca1 | Majorly promotes the spreading of DNA damage response proteins and chromatin changes in meiotic silencing; has a minor role in meiotic recombination as regulating the timing of crossover formation | Meiotic silencing; meiotic recombination | Complete arrest after mid-pachytene, sterile | Decreased numbers of MSH4 foci and the delayed appearance of MLH1 foci, largely depleted H2AX signals at the X-centromeric end and its surrounding pericentric region | Normal fertility |  | 24914237 | Conditional, germline-specific deletion |
| 12190 | Brca2 | Required for the recruitment of recombinases on DSBs in meiotic recombination | Meiotic recombination | Complete arrest at zygotene-pachytene. sterile | Lack of extensive synapsis, reduced number of RAD51 foci but abundant RPA foci | Some could progress through meiotic prophase I, with a high frequency of nuclear abnormalities, massive oocyte loss for 2-3 weeks postnatally, infertile | | 14660434 | Homozygous mutant carrying a human BRCA2 gene |
| 74666 | Brme1 | Stabilizes MEILB2; recruits recombinases via BRCA2-MEILB2-BRME1 complex during in meiotic recombination | Meiotic recombination | Arrest at pachytene with a small subset cell entering diplotene and undergoing apoptosis later, sterile | Disrupted DSB repair with decreased number of HSF2BP and DMC1/RAD51 foci, leading to failed synapsis and crossover recombination | Normal fertility |  | 32463460; 32345962 |  |
| 229776 | Cdc14a | Dual-specificity phosphatase that counteracts CDK and is required for meiotic recombination initiation | Meiotic recombination initiation | Partial arrest at pachytene stage, subfertile | Decreased γH2ax signals in germ cells | Fertile |  | 32679235 |  |
| 12566 | Cdk2 | A serine/threonine protein kinase that governs the structure of the nuclear envelope and the telomere-led chromosome movements; essential for homolog pairing, synapsis and DSB processing in males; promotes proper dynamics of SYCP3 in females | Chromosome movements; homolog pairing and synapsis; DSB processing | Complete arrest prior to mid-pachytene, sterile | Incomplete chromosome pairing, an extensive non-homologous synapsis unrepaired DSBs; in some of these spermatocytes, telomeres do not attach to the nuclear envelope, and sex chromosomes do not form a sex body | Arrest at dictyate stage, oocyte depletion by 14 dpp, infertile | Improper distribution of SYCP3 | 19494131;12923533 |  |
| 28135 | Cep63 | A centrosomal protein that might be required for chromosome dynamics in males | chromosome movements | Partial pachytene arrest, sterile | Aberrant DNA damage response triggered by centrosome-based mitotic errors. Impaired meiotic recombination, numerical and structural centrosome aberrations, chromosome entanglements and defective telomere clustering | Reduced follicles, fertile |  | 26158450 |  |
| 13164 | **Dazl** | RNA-binding protein that mediates the translation of key proteins for the SC formation and DSB repair during spermatogenesis | SC formation; meiotic DSB repair | Arrest at early pachytene/pachytene-like stage, sterile | *N.A.* | Arrest at pachytene-diplotene stage, depletion of oocytes by 4dpp, infertile | | 31355046;9288969; 14611631 | Germ cell specific conditional knockout |
| 13404 | **Dmc1** | Meiosis-specific recombinase that catalyses strand invasion into intact homologous duplexes | Meiotic recombination | Mostly arrest at zygotene/zygotene-like stage, sterile | Failed homolog pairing and synapsis, persistent RAD51 foci | Arrest at pachytene-like stage, most eliminated by 4 pp, nearly devoid of oocytes by 18 dpp, infertile | | 9660953; 9660954; 25636482 |  |
| 54427 | Dnmt3l | Mediates DNA methylation that influences molecular interactions important for chromatin compaction in spermatocytes | DNA methylation; chromatin modification | Complete arrest at early pachytene, sterile | Failed maternal methylation imprint establishment, aberrant chromatin packaging and failed homolog pairing and synapsis | Fertile |  | 11934864; 15753313 |  |
| 71241 | Dmrt7 | Mediates a transition in histone modifications that maintains transcriptional silencing of the sex chromosomes during prophase I in males | Meiotic silencing | Arrest at early-mid pachytene stage with a small proportion can progress to diplotene or beyond, sterile | Normal meiotic pairing and recombination and sex body formation, abnormal sex chromatin | Normal fertility |  | 17098235; 17447844 | The function is concluded due to the surviving diplotene cells, thus it's likely not linking to the arrest; or these transition defects elicit arrest or there might be other functions to detect or more. |
| 94244 | Fkbp6 | Co-chaperone that is required to repress transposable elements and prevent their mobilization during spermatogenesis; acts via the piRNA metabolic process; controls homologous chromosomes pairing and synapsis specifically in males. | Silencing of retrotransposon | Complete arrest at pachytene stage, sterile | Abnormal pairing and misalignments between homologous chromosomes, nonhomologous partner switches, and autosynapsis of X chromosome cores | Normal fertility |  | 12764197 |  |
| 60611 | Foxj2 | Transcriptional activator that regulates likely the DSB repair genes during meiotic recombination | Meiotic recombination | Complete arrest at pachytene stage, sterile | Failed chromosomal synapsis and DSB repair | *N.A.* |  | 27316861 |  |
| 15270 | H2ax | Variant histone H2A that involves in DSB repair factors assembly, meiotic silencing, checkpoint signalling transducing and telomere clustering | Meiotic DSB repair, meiotic silencing; meiotic checkpoint; chromosome movements | Arrest at pachytene stage, with some could reach diplotene, sterile | Failed sex body formation and MSCI initiation, severe defects in meiotic pairing | Subfertile |  | 11934988; 12689589; 14530383 |  |
| 15201 | Hells | Forms a pioneer complex with PRDM9 to open chromatin at meiotic recombination hot spots in males; essential for the transcriptional repression of repetitive elements in females. | Programmed DSB formation in males; transcriptional repression in females | Arrest at early- mid pachytene, sterile. | Defective homologous chromosome synapsis, lack of XY body | Lethal after birth | Incomplete chromosome synapsis associated with persistent RAD51 foci and γH2AX phosphorylation, failure to load crossover-associated foci | 21349825; 17115026 |  |
| 19183 | **Hop2** | Required for proper homologous chromosome pairing and efficient DSB repair and cross-over during meiosis; stimulates both DMC1- and RAD51-mediated strand assimilation when forming heterodimer with Mnd1; may alone functions as a recombinase to promote formation of strand invasion by the NCO pathway, independently of DMC1 and RAD51 | Meiotic recombination; homolog paring | Complete arrest at pachytene-like stage, sterile | Defective synapsis and DSB repair with different extents | Absence of follicles in adult ovaries, infertile | | 14667414; 24304900 |  |
| 67981 | Hormad1 | Axis component, promotes DSB formation; promotes SC formation independently of its role in homology search; plays a key role in the male mid-pachytene checkpoint and the female meiotic prophase checkpoint | SC formation; meiotic DSB repair; meiotic checkpoint | Complete arrest at pachytene stage, sterile | Incomplete homolog pairing and synapsis, lack of XY body; disrupted meiotic recombination (less DSB formation, reduced number of RPA, RAD51 and DMC1 foci, dramatically reduced MLH1) | Grossly normal ovarian development, arrest at blastocytes stage due to aneuploidy leading to infertility | Incomplete homolog pairing and synapsis, disrupted meiotic recombination (less DSB formation, reduced number of RPA, RAD51 and DMC1 foci) | 21079677; 21478856 |  |
| 75828 | Hormad2 | Promotes the accumulation of DDR components along the unsynapsed chromatin, but not at DNA DSBs or on DNA DSB-associated chromatin loops, thereby ensuring meiotic silencing; essential for meiotic checkpoint in response to asynapsis | Meiotic silencing and meiotic checkpoint | Complete arrest at mid-pachytene stage, sterile | Grossly normal homologous synapsis and meiotic recombination (DSB formation, early recombination processes), defective XY body, no presence of cells forming COs | Normal fertility |  | 23039116; 22549958 |  |
| 434438 | Iho1 | Required for DSB formation; probably acts by forming a complex with MEI4 and REC114, which activates DSBs formation, an essential step to ensure completion of synapsis | Programmed DSB formation; synapsis | Arrest at mid pachytene stage, sterile | Defective DSB formation and homologous synapsis | Nearly complete absence of oocytes in ovaries at 6-weeks, infertile | Defective homologous synapsis | 27723721 |  |
| 69260 | Ing2 | Regulates chromatin modification and functionally interacts p53 | Chromatin modification | Arrest before/at pachytene stage, sterile | Incomplete recombination | Normal fertility |  | 21124965 |  |
| 384619 | **Kash5** | Bridges the nuclear envelope and telomeres through forming LINC complex with SUN1/2 proteins, thereby promoting chromosomal movements and homolog pairing | Chromosome movements; homolog pairing | Arrest at zygotene/pachytene stage, sterile | Defective pairing and synapsis, failed DSB repair | Depletion of follicles in adult ovaries, infertile | | 24062341; 24586178;22826121 |  |
| 110958 | **M1ap** | Unknown function, required for meiosis I progression during spermatogenesis | Unknown | Partially at the zygotene/pachytene stage, while most cells advanced to metaphase I before arresting and entering apoptosis, sterile | Defective synapsis, DSB repair and CO formation | Normal fertility |  | 23269666 |  |
| 98558 | Mael | A component of nuage, essential for transposon silencing in germ cells | Silencing of retrotransposon | Complete arrest at early pachytene stage, sterile | defective synapsis, persistent SPO11-independent DNA damage, delayed meiotic entry | 3-fold reduction in the number of fetal oocytes at birth, increased aneuploidy | Defective homologous chromosome synapsis, DSB repair and CO formation | 18694567;24882376 |  |
| 622554 | Majin | Links telomere-nuclear envelope through forming TERB1/2-MAJIN complex; promotes meiotic chromosome movements and homologous pairing | Chromosome movements; homolog pairing | Complete arrest at zygotene stage, sterile | Impaired homolog pairing and synapsis | Complete arrest at zygotene stage; depletion of follicles in adult ovaries, infertile | Impaired homolog pairing and synapsis | 26548954; 30718482 |  |
| 240697 | Mcmdc2 | Plays an important role in meiotic recombination; associated with the formation, or the stabilization, of DNA strand invasion events that promote homolog pairing and DSB repair | Meiotic recombination | Complete arrest at early pachytene stage, sterile. | Defective homolog pairing, synapsis and DSB repair, absent CO formation | Oocyte loss perinatally /soon after birth, complete devoid of oocytes in ovaries at 6-weeks, infertile | Defective homolog pairing, synapsis and DSB repair, absent CO formation | 27760146;27986806 |  |
| 74369 | Mei1 | Required for normal meiotic chromosome synapsis; epistatic to *Dmc1,* likely involved in the meiotic DSB formation in spermatocytes | Programmed DSB formation; synapsis | Complete arrest at zygotene/pachytene stage, sterile | Defective homolog synapsis and DSB formation, intact recombinational DSB repair | Arrest at zygotene/pachytene stage, with a small number progressing to metaphase I with unpaired homologs and attempting the first meiotic division, reduced numbers of follicles, infertile | Defective homolog pairing and synapsis | 11820814; 14668445; 15928951 |  |
| 75033 | Mei4 | Required for DSB formation; probably acts by forming a complex with IHO1 and REC114, which activates DSB formation, an essential step to ensure completion of synapsis | Programmed DSB formation; synapsis | Complete arrest at mid/late pachytene stage, sterile | Defective DSB formation and homologous synapsis | Nearly complete of follicles in ovaries at 8-weeks, infertile | | 20551173; 25795304;27723721 |  |
| 74377 | **Meilb2** | Binds and localizes BRCA2 to DSB sites; required for proper recombinase recruitment to DSB sites via BRCA2-MEILB2-BRME1 complex during in meiotic recombination | Meiotic recombination | Complete arrest at zygotene-pachytene. sterile | Abolished localization of RAD51 and DMC1 in spermatocytes | Partial meiotic prophase arrest, survived oocyte complete recombination with normal numbers of bivalent chromosomes, reduced oocyte reserve, subfertile | Impaired recombinase DMC1 and RAD51 recruitment, but milder than that in males | 30760716 |  |
| 75178 | **Meiob** | Single-stranded DNA-binding protein that is required for homologous recombination and promotion of faithful and complete synapsis in meiosis I; forms complex with SPATA22, likely to ensure the stabilization of recombinases; displays single-stranded DNA 3'-5' exonuclease activity *in vitro* | Meiotic recombination; synapsis | Complete arrest at zygotene-like stage, sterile | Defective homolog synapsis, DSB repair and CO formation | Complete oocyte loss by 2 dpp, infertile | Defective homolog synapsis, DSB repair and CO formation | 24240703; 4068956 |  |
| 76915 | **Mnd1** | Required for proper homologous chromosome pairing and efficient DSB repair and cross-over during meiosis; stimulates both DMC1- and RAD51-mediated strand assimilation when forming heterodimer with HOP2 | Meiotic recombination; homolog paring | Arrest at zygotene/pachytene like stage, sterile | Defective synapsis and DSB repair with different extents | Absence of follicles in adult ovaries, infertile | | 24304900 |  |
| 240069 | Morc2b | Required for chromosomal synapsis and meiotic recombination in males and females | Programmed DSB formation; synapsis | Complete arrest at pachytene-like stage, sterile | Defective synapsis and meiotic recombination | Complete oocyte loss by 2 dpp, infertile | Defective synapsis and meiotic recombination | 29329290 |  |
| 83456 | **Mov10l1** | ATP-dependent RNA helicase required during spermatogenesis to repress transposable elements and prevent their mobilization, which is essential for germline integrity | Silencing of retrotransposon | Complete arrest at zygotene-like stage, sterile | Defective synapsis, loss of DNA methylation and subsequent derepression of retrotransposons | Normal fertility |  | 20534472; 20547853  23166510; 25762440 | Conditional mutant |
| 17535 | Mre11 | Component of the MRN complex, which governs meiotic DSB repair by facilitating DSB end resection and checkpoint signalling via activation of the ATM kinase during meiosis | Meiotic DSB repair; meiotic checkpoint | Temporal disturbance in meiotic progression, fertile | Defective synapsis and DSB repair | Temporal disturbance in meiotic progression, subfertile | Defective synapsis and DSB repair | 14690604; 17291760 | Hypomorphic mutant |
| 55993 | **Msh4** | DNA mismatch repair protein that locates at recombination nodules, facilitating homolog paring and crossover formation | Homolog paring; crossover formation | Complete arrest at zygotene stage, sterile | Failed homolog pairing and synapsis, incomplete meiotic recombination | Loss of the majority oocytes by 4 dpp, before dictyate stage, infertile | Failed homolog pairing and synapsis, incomplete meiotic recombination | 10809667 |  |
| 17687 | **Msh5** | DNA mismatch repair protein that Locates at recombination nodules, facilitating homolog paring and crossover formation | Homolog paring; crossover formation | Complete arrest at zygotene stage, sterile | Failed homolog synapsis | Arrest at zygotene stage with some cells surviving to a post-pachytene stage, absence of follicles and oocytes in adult ovaries, infertile | Failed homologous synapsis | 10072381 |  |
| 17864 | Mybl1 | Transcription factor that promotes expression of piRNAs in male meiosis, which form complexes with Piwi proteins, mediating the repression of transposable elements | Silencing of retrotransposon | Arrest at epithelial Stage IV/mid-pachytene stage, sterile | Defective synapsis and DSB repair, lack of meiotic crossovers | Normal fertility | Normal progressions of synapsis, DSB repair and CO formation | 21750041 |  |
| 27354 | Nbs1 | Component of the MRN complex, which governs meiotic DSB repair by facilitating DSB end resection and checkpoint signalling via activation of the ATM kinase during meiosis | Meiotic DSB repair; meiotic checkpoint | Complete arrest at zygotene stage, sterile | Defective synapsis and homologous recombination repair | Depletion of oocytes at diplotene stage, absence of oocytes in adult ovaries, infertile | Synapsis is completed in pachytene oocytes | 31965061 | Male-conditional germ-specific knockout; female-knockout mice rescued by BAC containing human NBS1 mutant |
| 57746 | Piwil2 | Endoribonuclease that mediates the repression of transposable elements during meiosis by forming complexes composed of piRNAs and Piwi proteins and govern the methylation and subsequent repression of transposons | Silencing of retrotransposon | Complete arrest at zygotene/pachytene stage, sterile | *N.A.* | Normal fertility |  | 14736746 |  |
| 330890 | Piwil4 | Mediates the repression of transposable elements during meiosis by forming complexes composed of piRNAs and Piwi proteins and govern the methylation and subsequent repression of transposons | Silencing of retrotransposon | Complete arrest prior to pachytene stage, sterile | Defective synapsis and DSB repair | Normal fertility |  | 17395546 |  |
| 194908 | Pld6 | Presents endonuclease activity and plays a critical role in piRNA biogenesis, which mediates the repression of transposable elements during male meiosis | Silencing of retrotransposon | Complete arrest at zygotene/early pachytene stage, sterile. | Defective synapsis | Normal fertility |  | 21397848;21397847 |  |
| 213389 | Prdm9 | Histone methyltransferase that determines hotspot localization for DSB formation via binding specific DNA sequences through its zinc finger domains, thereby promoting meiotic recombination; during meiotic progression hotspot-bound PRDM9 interacts with several complexes, subsequently controlling the DSB repair pathway, pairing of homologous chromosomes and sex body formation | Meiotic recombination | Complete arrest at pachytene stage, sterile. | Severe impaired DSB repair, deficient pairing of homologous chromosomes and impaired sex body formation | Arrest at pachytene stage, massive oocyte loss perinatally, absence of follicles at 5-weeks, infertile | Severe impaired DSB repair and deficient pairing of homologous chromosomes | 16292313; 27932493 |  |
| 668929 | Rad21l | Meiosis-specific component of some cohesin complex; required during the initial steps of prophase I in male meiosis, including synaptonemal complex assembly, synapsis initiation and crossover recombination between homologous chromosomes; uniquely required for normal pericentromeric heterochromatin clustering events; not required for meiosis in females in young mice, while it is required later as mice age | Component of cohesin complex; SC formation; synapsis initiation; meiotic recombination | Complete arrest at zygotene-like stage, sterile | Defective homologous chromosome synapsis and DSB repair and absence of CO formation | Fertile with an age-dependent sterility/subfertile | Mild defects in homologous synapsis | 21743440; 27172213 |  |
| 114714 | Rad51c | Facilitates RAD51 formation, thereby promoting RAD51-mediated meiotic recombination; possibly plays a role in HJ resolution during late phase of meiotic recombination | Meiotic recombination | Incomplete arrest at pachytene stage, sterile | Defective synapsis and DSB repair on limited chromosomes, reduced CO formation | Infertile due to ovulation block | After superovulation, defects occur during metaphase I, leading to chromosomal abnormality | 17312021 | Hypomorphic mutation |
| 3673 | **Rec114** | Required for DSB formation; probably acts by forming a complex with IHO1 and MEI4, which activates DSBs formation, an essential step to ensure completion of synapsis | Programmed DSB formation; synapsis | Complete arrest at zygotene-like stage, sterile | Defective DSB formation and homologous synapsis | Complete arrest at zygotene-like stage, nearly complete absence of follicles in ovaries at 8-weeks, infertile | Defective DSB formation and homologous synapsis | 30569039; 27723721 |  |
| 56739 | **Rec8** | Key component of the meiotic cohesin complex that limits synapsis between homologous chromosomes; required for homologous recombination and the separation of homologous chromosomes | Component of cohesin complex; SC elongation; meiotic recombination; separation of homologous chromosomes | Complete arrest at zygotene-like stage, sterile | Disrupted homologous chromosome synapsis, likely defective DSB repair and absence of CO formation. | Arrest at zygotene-like stage, absence of oocytes and follicles by 5dpp, infertile | Defective homolog synapsis | 15935783 |  |
| 108086 | Rnf216 | E3 ubiquitin ligase that regulates PKA stability during meiosis | Ubiquitination | Incomplete arrest at zygotene stage, sterile | *N.A.* | Normal fertility |  | 33724554 |  |
| 100155 | **shoc1** | ATPase required for the formation of crossover recombination intermediates in meiotic prophase I in male and female germ cells | Meiotic recombination | Complete arrest at zygotene-like stage, sterile | Incomplete synapsis, failed meiotic recombination with persistent recombination intermediates and absence of crossing over | Rapid oocyte loss since E17.5-1dpp, complete depletion of oocytes by 6dpp, infertile | Incomplete synapsis, failed meiotic recombination with persistent recombination intermediates and absence of COs | 30272023 |  |
| 75801 | **Six6os1** | Central element component of the synaptonemal complex | Element of SC | Complete arrest at pachytene-like stage, sterile | Failed synapsis between homologs, most of which are properly paired. Persistent DSBs, RAD51/DMC1 foci and MSH4, absence of MLH1. | Complete arrest at pachytene-like stage, oocyte depletion by 6 dpp, infertile | Failed synapsis between homologs, most of which are properly paired, persistent DSBs, RAD51/DMC1 foci and MSH4, absence of MLH1 | 27796301 |  |
| 21402 | Skp1 | Ubiquitin E3 ligase that plays crucial and distinct roles in meiotic DSB homeostasis, synapsis initiation and maintenance and sister chromatid cohesion during meiotic prophase I | Meiotic DSB homeostasis; synapsis initiation and maintenance; sister chromatid cohesion | Complete arrest at zygotene-like stage | Defective synapsis and early meiotic recombination | Arrest at pachytene-like stage, loss of the majority oocytes at 1dpp and complete loss by 6 weeks | Defective synapsis, less defective DSB repair compared to males | 35489071 | Conditional inactivation in germ cells prior to meiosis |
| 73333 | Slc25a31 | ADP: ATP antiporter that mediates the translocation of ADP and ATP across the inner mitochondrial membrane; supports DSB repair and synapsis progression that require sufficient ATP supplies during meiotic prophase I | Meiotic DSB repair; synapsis | Partially arrest at pachytene stage, sterile | Defective synapsis and DSB repair in partial pachytene cells, the other cells likely be eliminated due to the deficiency of ATP supplies from mitochondria. | Normal fertility |  | 17681941; 19556438 |  |
| 140557 | **Smc1b** | Meiosis-specific component of cohesin complex; required for the maintenance of meiotic cohesion and only to a minor extent, for its establishment; required for stable telomere attachment to the nuclear envelope; contributes to AE formation and the organization of chromatin loops along the AE | Component of cohesin complex; synapsis; meiotic recombination; chromosome movements | Arrest at early-mid pachytene stage, sterile | Incomplete synapsis (shortened SC, discontinuous SC and unsynapsed AEs), defective DSB repair on asynapsed chromosomes, absence of COs. | Progress to dictyate stage but exhibit massive aneuploidy during the meiotic divisions, absence of follicles in 5-7 months old ovaries | Incomplete synapsis/reduced SC length and reduced CO numbers | 15146193; 18180366; 19841137;16258540 | |
| 380709 | Spata22 | Meiosis-specific protein that forms complex with MEIOB, likely to ensure the stabilization of recombinases during meiotic recombination | Meiotic recombination | Complete arrest at zygotene-like stage/before mid-pachytene stage/epithelial stage IV, sterile | Impaired homolog synapsis and DSB repair. | Almost complete absence of oocytes by 10 dpp, infertile | Impaired homolog synapsis and DSB repair | 22011390; 24240703 |  |
| 70891 | Spdya | Regulates telomere-NE attachment and NE structure by interacting with CDK2 and SUN1; critical for homolog pairing and synapsis | Homolog pairing and synapsis | Complete arrest epithelial stage IV, mostly before mid pachytene stage, sterile | Defective homolog synapsis with extensive non-homologous pairing and telomere fusions, impaired late-recombination stages, and absence of COs. | Arrest at pachytene/pachytene-like stage, absence of follicle in adult ovaries, infertile | Defective homolog synapsis with extensive non-homologous pairing and telomere fusions, reduced CO formation | 27025256; 34039995 |  |
| 26972 | **Spo11** | Component of a component of TOPOVIL complex, with TOPOVIBL; mediates DNA cleavage that forms the DSB together with TOPOVIBL; Essential for the phosphorylation of SMC3, HORMAD1 and HORMAD2 | Programmed DSB formation | Complete arrest at zygotene-like/early-mid pachytene stage, sterile | No synapsis or little synapsis between non-homologous chromosomes, meiotic recombination doesn't initiate. | Survive to diplotene stage with reduced numbers of oocyte, complete depletion of oocytes in 2-3 months old ovaries, infertile | No synapsis or little synapsis between non-homologous chromosomes, meiotic recombination doesn't initiate | 11106739; 11106738; 15640358; 16055729 | |
| 50878 | **Stag3** | Meiosis specific component of cohesin complex; required for AE formation and the stability of meiosis-specific cohesins | Component of cohesin complex; SC formation | Complete arrest at zygotene-like stage without reaching pachytene/stage IV, sterile | Disrupted AE formation and homologous chromosome synapsis, failed DSB repair. | Complete depletion of oocytes by 7 dpp, infertile | Disrupted AE formation, homologous synapsis, failed DSB repair | 24597867; 24608227; 24992337; 27172213; 24797475 | Common phenotypes summarized; phenotypes observed in these studies differ somewhat depending on the knockout allele of Stag3 |
| 77053 | Sun1 | Bridges the nuclear envelope and telomeres through forming LINC complex with KASH5 proteins, thereby promoting chromosomal movements and homolog pairing | Chromosome movements; homolog pairing | Complete arrest at pachytene-like stage, sterile | Disrupted telomere NE Attachment, impaired homologous chromosome pairing, synapsis, and recombination | Complete arrest at pachytene-like stage, oocyte depletion by 5 dpp, infertile | Disrupted telomere NE Attachment, impaired homologous chromosome pairing and synapsis | 17543860 |  |
| 74075 | **Syce1** | Major component of the transverse central element of SC; essential for synapsis initiation; have roles in the the assembly and stabilization of SC and the stabilization of homolog interactions during meiotic recombination | Element of SC | Arrest at epithelial stage IV, sterile | Absent SC and sex body, aligned homologs at variable distances, incomplete DSB repair, absence of COs | Complete depletion of follicles in adult ovaries, infertile | Absent SC and sex body, aligned homologs at variable distances, incomplete DSB repair, absence of COs | 19247432 |  |
| 71846 | Syce2 | Major component of the transverse central element of SC; required for propagating synapsis along the paired chromosome axes | Element of SC | Arrest at epithelial stage IV, sterile | Failed SC formation between aligned homologs with small regions of synapsis, absence of sex body, incomplete DSB processing, absence of COs | Minute adult ovaries, infertile. | Failed SC formation between aligned homologs with small regions of synapsis, incomplete DSB processing and absence of COs | 17339376 |  |
| 75459 | Syce3 | Major component of the transverse central element of SC; required for the loading of other central element proteins, and for initiating synapsis between homologous chromosomes | Element of SC | Arrest at epithelial stage IV, sterile | Failed synapsis between mostly aligned AEs, persistent DSB, incomplete DSB repair and absence of COs | Complete depletion of follicles in adult ovaries, infertile | Affected homolog pairing and failed synapsis between homologs, persistent DSB, incomplete DSB repair and absence of COs | 21637789 |  |
| 20957 | Sycp1 | Major component of the transverse filaments of synaptonemal complexes. Essential for synapsis initiation and required for SC assembly and normal centromere pairing. | Element of SC | The majority arrest at pachytene stage with a proportion reaches diplotene or, exceptionally metaphase I, sterile | Absent SC between aligned homologs, persistent DNA damage and absence of COs and sex body | Absence of growing follicles and oocyte, infertile | | 5937223 |  |
| 320558 | **Sycp2** | Major component of the axial/lateral elements of SC; required for the SC assembly; may be involved in the organization of chromatin | Element of SC | Complete arrest at zygotene/zygotene-like stage, sterile | Failed AE formation and the subsequent disruption of homolog synapsis | Subfertile | Interrupted homolog synapsis but exhibiting SYCP1 localization, nuclear aggregates of SYCP3 | 16717126 |  |
| 20962 | **Sycp3** | Structural component of the axial/lateral element of the SC; required for centromere pairing in males and for chiasmata formation and for the structural integrity of meiotic chromosomes in females; required for efficient phosphorylation of HORMAD1 and HORMAD2 during meiotic prophase I | Element of SC | Complete arrest at zygotene, sterile | Failed homolog pairing, AE, and SC formation, affected distribution of RPA/RAD51 | Subfertile, severely reduced oocyte pool, exhibits a sharp reduction in litter size that increases with advancing maternal age due to aneuploid oocytes | Disrupted homolog synapsis but exhibiting SYCP1 localization, defective meiotic chromosome segregation | 10678170; 12004129 |  |
| 74691 | **Tdrd9** | TP-binding RNA helicase that acts functionally with MIWI2 via the piRNA metabolic process, thereby repressing transposable elements and preventing their mobilization | Silencing of retrotransposon | Complete arrest at zygotene stage, sterile | Failed homolog synapsis and incomplete meiotic recombination | Normal fertility |  | 20059948 |  |
| 320022 | **Terb1** | Links telomere-nuclear envelope through forming TERB1/2-MAJIN complex; promotes meiotic chromosome movements and homolog pairing | chromosome movements; homolog pairing | Complete arrest at zygotene-like stage, sterile | Defective homolog pairing and synapsis, impaired DSB repair and absent CO formation, disrupted telomere NE Attachment | Complete arrest at zygotene-like stage; depletion of follicles in adult ovaries, infertile | Defective homolog pairing and synapsis | 24413433; 30718482 |  |
| 74401 | **Terb2** | Links telomere-nuclear envelope through forming TERB1/2-MAJIN complex; promotes meiotic chromosome movements and homolog pairing | chromosome movements; homolog pairing | Complete arrest at zygotene stage, sterile | Impaired homolog pairing and synapsis | Complete arrest at zygotene stage; depletion of follicles in adult ovaries, infertile | Impaired homolog pairing and synapsis | 26548954; 30718482 |  |
| 17771 | Tesmin | Translocates into the nuclei around the zygotene-pachytene transition and promotes meiosis progression beyond the mid-pachytene stage during spermatogenesis. | Meiosis progression | Complete arrest at early pachytene stage, sterile | Roughly normal meiotic recombination progression and minor defects in synapsis | Fertile |  | 34388164; 31916570 |  |
| 83558 | **Tex11** | Involves in initiation and/or maintenance of chromosome synapsis and formation of crossovers during meiosis | Synapsis; crossover formation | Partially arrest at pachytene stage, the remaining cells arrest at anaphase I, sterile | Asynapsis in partial spermatocytes and reduced COs | Fertile with reduced litter size | Asynapsis in partial oocytes and reduced COs | 18316482 |  |
| 66654 | Tex12 | Major component of the transverse central element of SC; Required for propagating synapsis along the paired chromosome axes | Element of SC | Complete arrest at epithelial stage IV, sterile | Failed elongation of synapsis between homologs, normal loading of early recombination markers with absence of sex body | Complete depletion of follicles by 7 dpp, infertile | Failed elongation of synapsis between homologs, impaired DSB processing and absence of COs | 18611960 |  |
| 104271 | **Tex15** | Required for normal chromosomal synapsis and the formation of DMC1 and RAD51 foci on meiotic chromosomes; essential executor of PIWIL4-piRNA pathway directed transposon DNA methylation and silencing in males | Meiotic recombination; synapsis; silencing of retrotransposon | Complete arrest at early pachytene stage, sterile | Failed homolog synapsis and meiotic recombination with the lack of RAD51/DMC1 | Normal fertility |  | 18283110; 32381626; 32719317 |  |
| 73679 | Tex19.1 | Promotes SPO11-dependent meiotic recombination with UBR2 in males and maintain sister chromatid cohesion postnatally in females; participates in the repression of retrotransposable elements and preventing their mobilization in males | Meiotic recombination; sister chromatid cohesion; silencing of retrotransposon | Heterogenous phenotypes, from partial to complete meiotic arrest at pachytene stage, varied fertility from subfertile to infertile | Defective early meiotic recombination, failed homology synapsis | Subfertile | Unaffected early recombination and homolog synapsis, premature sister chromatid separation and homologue missegregation during meiosis I | 18802469; 21103378; 28708824; 32232464 | |
| 381196 | Top6bl | Component of a topoisomerase 6 complex with SPO11; together with SPO11, mediates DSC formation in meiotic recombination | Meiotic recombination; homolog synapsis | Complete arrest before or at mid-pachytene stage, sterile | Defective homologous synapsis and little synapsis between non-homologous chromosomes; failed recombination initiation | Largely depletion of primordial and primary follicles at 30dpp |  | 26917764 |  |
| 69716 | **Trip13** | Promotes early steps upstream of the assembly of RAD51 complexes in meiotic recombination that leads to non-crossovers pathways; needed for efficient completion of homologous synapsis and SC formation; required for development of higher-order chromosome structures; required for depletion of HORMAD1 and HORMAD2 from synapsed chromosomes | Meiotic recombination; synapsis | Complete (*Trip13^sev/sev^*) or incomplete (*Trip13^mod/mod^*) arrest at pachytene stage/ epithelial stage IV, sterile | Defective synapsis between autosomal homologs (only displayed in *Trip13^sev/sev^*), failed XY body formation and DSB repair, reduced COs (only displayed in *Trip13^sev/sev^*) | Depletion of follicles by 21dpp in *Trip13^sev/sev^;* by 2 months in *Trip13^mod/mod^*, infertile | Defective synapsis between autosomal homologs and reduced COs (only displayed in *Trip13^sev/sev^*) failed DSB repair | 17696610; 20711356 |  |
| 224826 | Ubr2 | E3 ubiquitin-protein ligase that plays a critical role in chromatin inactivation and chromosome-wide transcriptional silencing during meiosis via ubiquitination of histone H2A; promotes SPO11-dependent recombination foci to accumulate and drive robust homologous chromosome synapsis with Tex19.1; involves in inhibiting LINE-1 retrotransposon mobilization | Meiotic recombination; synapsis; silencing of retrotransposon; meiotic silencing | Incomplete arrest at pachytene stage, sterile | Defective during early DSB repair, disrupted homolog pairing and synapsis | Surviving females from embryonic lethality are fertile | | 14585983; 22616001; 28708824 | Variable defects in spermatogenesis are identified in mice with other strain background PMID 20080676 |
| 381678 | Zcwpw1 | Dual histone methylation reader specific for PRDM9-catalyzed histone marks (H3K4me3 and H3K36me3) that facilitates the repair of PRDM9-induced meiotic DSB in males | Meiotic DSB repair | Complete arrest at zygotene/pachytene stage, sterile | Complete failed homology synapsis, accompanied by incomplete DSB repair and lack of COs | Normal fertility until mid-adulthood | Completion of meiosis with delayed meiotic prophase I | 31453335; 32374261; 2352380; 32744506 | |
| 22697 | Zscan21 | Strong transcriptional activator that plays an important role in the progression of meiotic prophase I in spermatocytes | Transcriptional activator |  | Failed homolog synapsis, impaired DSB with decreased RAD51 foci, reduced COs | *N.A.* |  | 27492080 | Spermatocyte-specific gene knockout |

Genes that are functional during meiotic prophase I and the mutations of which cause meiotic prophase arrest in mice are summarized. **Bold** genes in mice that have the corresponding variants identified in human patients (Table S2). ^a^ Spermatocytes arrest due to pachytene checkpoint response are sometimes found at cytologically zygotene/zygotene-like stage, which are equivalent to histologically pachytene stage in seminiferous tubules.
