# Supplementary material for: Genetic control of meiosis surveillance mechanisms in mammals
Source: Front Cell Dev Biol. 2023 Feb 23;11:1127440. doi: 10.3389/fcell.2023.1127440 (PMC9996228; doi:10.3389/fcell.2023.1127440)
Supplement: Supplementary file 3 [file Table2.docx]

| **Gene ID** | **Symbol** | **Variation Human** | **Conditions** | **Meiotic/testicular phenotype** | **Clinical significance** | **PMID/ClinVar accession** | **Comment** |
| --- | --- | --- | --- | --- | --- | --- | --- |
| 625662 | Ankrd31 | NM_001372053.1(ANKRD31):c.1565-2A>G | Genetic non-acquired premature ovarian failure |  | Pathogenic | VCV001120014.1 |  |
|  |  | NM_001372053.1(ANKRD31):c.985C>T (p.Gln329Ter) | Genetic non-acquired premature ovarian failure |  | Pathogenic | VCV001120015.1 |  |
| 13164 | Dazl | NM_001351.4(DAZL):c.160A>G (p.Thr54Ala) | Spermatogenic failure, susceptibility to |  | risk factor​ | 12414900 | Varied phenotypes in human patients without clear MP1 arrest. |
| 13404 | Dmc1 | NM_007068.4(DMC1):c.860C>A (p.Pro287His) | Azoospermia |  | Pathogenic | VCV001328945.1 |  |
|  |  | NM_007068.4(DMC1):c.598A>G (p.Met200Val) | Premature ovarian failure |  | Benign​ | 18166824 |  |
|  |  | NM_007068.4(DMC1):c.364A>G (p.Thr122Ala) | Azoospermia |  | Pathogenic | VCV001328944.1 |  |
| 19183 | Hop2 | NM_016556.4(PSMC3IP):c.338-15C>G | Ovarian dysgenesis 3 |  | Benign​ | VCV001285798 |  |
|  |  | NM_016556.4(PSMC3IP):c.-35C>T | Ovarian dysgenesis 3 |  | Uncertain significance​ | VCV000801407 |  |
|  |  | NM_016556.2(PSMC3IP): c.600_602del (p.Glu201del) | Ovarian dysgenesis 3 |  | / | 21963259 |  |
| 384619 | Kash5 | NM_144688.5(KASH5):c.747G>A (p.Ala249=) | Genetic non-acquired premature ovarian failure |  | Likely pathogenic​​ | VCV001232307 |  |
|  |  | NM_144688.5(KASH5):c.1146+5G>A | Azoospermia |  | Pathogenic | VCV001328949 |  |
|  |  | seq [GRCh37] del(19) (19q13.33) chr19: g.49894043-49903011del | non-obstructive azoospermia | Arrest at zygotene-like stage with a deficiency in homolog pairing and synapsis | / | 35674372 | From abstract of this paper |
|  |  | NM_144688: c.979_980del: p. R327Sfs*21 |  |  | / |  |  |
| 110958 | M1ap | NM_001321739.2(M1AP):c.1435-1G>A | Spermatogenic failure 48, Spermatogenesis maturation arrest |  | Pathogenic | 32017041 |  |
|  |  | NM_001321739.2(M1AP):c.1289T>C (p.Leu430Pro) | Non-obstructive azoospermia |  | Uncertain significance​ | VCV000805832.2 |  |
|  |  | NM_001321739.2(M1AP):c.1166C>T (p.Pro389Leu) | Spermatogenic failure 48, non-obstructive azoospermia | Maturation arrest at round spermatid stage | Uncertain significance​ | 32673564 |  |
|  |  | NM_001321739.2(M1AP):c.949G>A (p.Gly317Arg) | Spermatogenic failure 48, non-obstructive azoospermia | Maturation arrest at round spermatid stage | Uncertain significance​ | 32673564 |  |
|  |  | NM_001321739.2(M1AP):c.797G>A (p.Arg266Gln) | Spermatogenic failure 48, Cryptozoospermia, Non-obstructive azoospermia | Predominant meiotic arrest with occasional postmeiotic germ cells | Uncertain significance​ | 32673564 |  |
|  |  | NM_001321739.2(M1AP):c.676dup (p.Trp226fs) | Spermatogenic failure 48, non-obstructive azoospermia | Meiotic arrest at spermatocyte stage | Pathogenic | 32673564 |  |
|  |  | NM_001321739.2(M1AP):c.148T>C (p.Ser50Pro) | Non-obstructive azoospermia |  | Uncertain significance​ | VCV000805833.2 |  |
| 74377 | Meilb2* | NM_007031.2(HSF2BP):c.557T>C (p.Leu186Pro) | Premature ovarian failure 19 |  | Likely pathogenic​​ | VCV001224546.1 |  |
|  |  | NM_007031.2(HSF2BP):c.500C>T (p.Ser167Leu) | Premature ovarian failure 19 |  | Pathogenic | 32845237 |  |
|  |  | NM_007031.2(HSF2BP):c.382T>C (p.Cys128Arg) | Premature ovarian failure 19 |  | Likely pathogenic​​ | VCV001224547.1 |  |
| 75178 | Meiob | NM_001163560.3(MEIOB):c.191A>T (p.Asn64Ile) | Spermatogenic failure 22 |  | Pathogenic | 28206990 |  |
| 76915 | Mnd1 | GRCh37/hg19 4q31.3(chr4:154316483-154325120) | Premature ovarian failure |  | Likely pathogenic​​ | 31042289 |  |
| 83456 | Mov10l1 | NM_018995.3(MOV10L1):c.743+5G>A | Azoospermia |  | Pathogenic | VCV001328947.1 |  |
|  |  | NM_018995.3(MOV10L1):c.2447G>T (p.Ser816Ile) | Spermatogenic failure 73 |  | Pathogenic | 35476666 |  |
|  |  | NM_018995.3(MOV10L1):c.2542G>A (p.Gly848Arg) | Spermatogenic failure 73 |  | Pathogenic | 35476666 |  |
| 55993 | Msh4 | NM_002440.4(MSH4):c.1025C>T (p.Thr342Ile) | Genetic non-acquired premature ovarian failure |  | Likely pathogenic​​ | VCV001256043 |  |
|  |  | NM_002440.4(MSH4):c.1063A>G (p.Ile355Val) | Genetic non-acquired premature ovarian failure |  | Likely pathogenic​​ | VCV001255997 |  |
|  |  | NM_002440.4(MSH4):c.1453C>T (p.Gln485Ter) | Non-obstructive azoospermia |  | Likely pathogenic​​ | VCV000992887 |  |
|  |  | NM_002440.4(MSH4):c.1686del (p.Lys562_Val563insTer) | Non-obstructive azoospermia |  | Likely pathogenic​​ | VCV000992888 |  |
|  |  | NM_002440.4(MSH4):c.1855A>G (p.Met619Val) | Genetic non-acquired premature ovarian failure |  | Likely pathogenic​​ | VCV001256012 |  |
|  |  | NM_002440.4(MSH4):c.2198C>A (p.Ser733Ter) | Non-obstructive azoospermia |  | Likely pathogenic​​ | VCV000992889 |  |
|  |  | NM_002440.4(MSH4):c.2222_2225del (p.Lys741fs) | Genetic non-acquired premature ovarian failure |  | Pathogenic | VCV001256044 |  |
|  |  | NM_002440.4(MSH4):c.2261C>T (p.Ser754Leu) | Oligospermia\|Premature ovarian insufficiency\|non-obstructive azoospermia |  | Pathogenic/Likely pathogenic​ | 33448284 |  |
|  |  | NM_002440.4(MSH4):c.2374A>G (p.Thr792Ala) | Genetic non-acquired premature ovarian failure |  | Likely pathogenic​​ | VCV001256001 |  |
|  |  | NM_002440.4(MSH4):c.2728C>T (p.Arg910Ter) | Genetic non-acquired premature ovarian failure |  | Pathogenic | VCV001256045 |  |
| 17687 | Msh5* | NM_172166.4(MSH5):c.75dup (p.Ser26fs) | Non-obstructive azoospermia |  | Pathogenic | 34755185 |  |
|  |  | NM_172166.4(MSH5):c.826C>T (p.Arg276Cys) | Genetic non-acquired premature ovarian failure |  | Likely pathogenic​​ | VCV001255996 |  |
|  |  | NM_172166.4(MSH5):c.964C>T (p.Arg322Cys) | Non-obstructive azoospermia |  | Likely pathogenic​​ | 34755185 |  |
|  |  | NM_172166.4(MSH5):c.1051C>G (p.Arg351Gly) | Genetic non-acquired premature ovarian failure |  | Pathogenic | VCV001256029 |  |
|  |  | NM_172166.4(MSH5):c.1459G>T (p.Asp487Tyr) | Premature ovarian failure 13, nonobstructive azoospermia |  | Pathogenic | 28175301; 34980881 |  |
|  |  | NM_172166.4(MSH5):c.1857del (p.Ala620fs) | Non-obstructive azoospermia |  | Pathogenic | 34755185 |  |
| 3673 | Rec114 | NM_001042367.2(REC114):c.397T>G (p.Cys133Gly) | Oocyte maturation defect 10 |  | Pathogenic | 31704776 |  |
|  |  | NM_001042367.2(REC114):c.546+5G>A | Oocyte maturation defect 10 |  | Pathogenic | 31704776 |  |
| 56739 | Rec8 | NM_001048205.2(REC8):c.91C>T (p.Arg31Cys) | Non-obstructive azoospermia |  | Likely pathogenic​​ | 31479588 |  |
|  |  | NM_001048205.2(REC8):c.624+1G>A | Premature ovarian insufficiency |  | Likely pathogenic​​ | VCV001214014 |  |
|  |  | NM_001048205.2(REC8):c.872C>T (p.Pro291Leu) | Premature ovarian insufficiency |  | Uncertain significance​ | VCV000619070 |  |
|  |  | NM_001048205.2(REC8):c.1035_1036dup (p.Ala346fs) | Premature ovarian insufficiency |  | Likely pathogenic​​ | VCV001214013 |  |
|  |  | NM_001048205.2(REC8):c.1057A>C (p.Thr353Pro) | Premature ovarian failure |  | Uncertain significance​ | VCV000929773 |  |
| 100155 | shoc1 | NM_001378211.1(SHOC1):c.1277_1278del (p.Glu426fs) | Non-obstructive azoospermia |  | Pathogenic | VCV001244232 |  |
| 75801 | Six6os1* | NM_174978.3(C14orf39):c.1180-3C>G | Spermatogenic failure 52, non-obstructive azoospermia | Incomplete synapsis, meiotic arrest the pachytene-like stage | Pathogenic | 33508233 |  |
|  |  | NM_174978.3(C14orf39):c.958G>T (p.Glu320Ter) | Spermatogenic failure 52, non-obstructive azoospermia | Incomplete synapsis, meiotic arrest at spermatocyte stage | Pathogenic | 33508233 |  |
|  |  | NM_174978.3(C14orf39):c.204_205del (p.His68fs) | Azoospermia, Non-obstructive azoospermia, Spermatogenic failure 52, Premature ovarian failure 18 | Complete asynapsis between homologs, meiotic arrest at the pachytene-like stage | Pathogenic | 33508233 |  |
| 140557 | Smc1b | NM_148674.5(SMC1B):c.863A>G (p.Glu288Gly) | Genetic non-acquired premature ovarian failure |  | Likely pathogenic​​ | VCV001256022 |  |
| 26972 | Spo11 | NM_012444.3(SPO11):c.744G>A (p.Thr248=) | Non-obstructive azoospermia |  | Likely pathogenic​​ | VCV001244233.1 |  |
| 50878 | Stag3* | NM_001282717.2(STAG3):c.48G>T (p.Leu16Phe) | Spermatogenic failure 61\|Premature ovarian failure 8 |  | Benign​ | VCV001236675 |  |
|  |  | NM_001282717.2(STAG3):c.106A>C (p.Thr36Pro) | Spermatogenic failure 61\|Premature ovarian failure 8 |  | Benign​ | VCV001183740 |  |
|  |  | NM_001282717.2(STAG3):c.291dup (p.Asn98fs) | Premature ovarian failure 8 |  | Pathogenic | 30006057 | Heterozygous pathogenic variant |
|  |  | NM_001282717.2(STAG3):c.562del (p.Gln188fs) | Premature ovarian failure 8 |  | Pathogenic | 24597867 |  |
|  |  | NM_001282717.2(STAG3):c.962G>A (p.Arg321His) | Premature ovarian failure 8\|Spermatogenic failure 61\|PRIMARY OVARIAN FAILURE 8 | | Pathogenic/Likely pathogenic​ | 32634216 |  |
|  |  | NM_001282717.2(STAG3):c.1069C>T (p.Arg357Ter) | Premature ovarian failure |  | Pathogenic | VCV000929755 |  |
|  |  | NM_001282717.2(STAG3):c.1245-26T>C | Spermatogenic failure 61\|Premature ovarian failure 8\|not provided |  | Benign​ | VCV001229759 |  |
|  |  | NM_001282717.2(STAG3):c.1262T>G (p.Leu421Arg) | Spermatogenesis maturation arrest\|non-obstructive azoospermia\|Spermatogenic failure 61 | Deficient chromosomal axis and SC formation | Likely pathogenic​​ | 31682730 | Heterozygous pathogenic variant |
|  |  | NM_001282717.2(STAG3):c.1293A>C (p.Pro431=) | Spermatogenic failure 61\|Premature ovarian failure 8\|not provided |  | Benign​ | VCV001243986 |  |
|  |  | NM_001282717.2(STAG3):c.1312C>T (p.Arg438Ter) | Spermatogenesis maturation arrest\|non-obstructive azoospermia\|Spermatogenic failure 61 | Deficient chromosomal axis and SC formation | Likely pathogenic​​ | 31682730 | Heterozygous pathogenic variant |
|  |  | NM_001282717.2(STAG3):c.1571del (p.Gln524fs) | Premature ovarian failure 8 |  | Pathogenic | VCV000869148 |  |
|  |  | NM_001282717.2(STAG3):c.1573+5G>A | Premature ovarian failure 8 |  | Pathogenic | 28393351 |  |
|  |  | NM_001282717.2(STAG3):c.1573+41C>G | Spermatogenic failure 61\|Premature ovarian failure 8\|not provided |  | Benign​ | VCV001287786 |  |
|  |  | NM_001282717.2(STAG3):c.1936dup (p.Ala646fs) | Spermatogenic failure 61 | Persistence of meiotic DSBs and a failure to complete chromosome pairing | Pathogenic | 31125047 | Compound heterozygosis, heterozygous for the first variant and homozygous for the second variant |
|  |  | NM_001282717.2(STAG3):c.2394+1G>A | Spermatogenic failure 61 |  | Pathogenic | 31125047 |  |
|  |  | NM_001282717.2(STAG3):c.1942G>A (p.Ala648Thr) | Non-obstructive azoospermia\|Premature ovarian insufficiency |  | Uncertain significance​ | 35176428 | Double homozygous for both variants |
|  |  | NM_001282717.2(STAG3):c.1953_1955del (p.Leu652del) | Non-obstructive azoospermia\|Premature ovarian insufficiency |  | Likely pathogenic​​ | 35176428 |  |
|  |  | NM_001282717.2(STAG3):c.1947_1948dup (p.Tyr650fs) | Premature ovarian failure 8 |  | Pathogenic | 26059840 |  |
|  |  | NM_001282717.2(STAG3):c.1950C>A (p.Tyr650Ter) | Premature ovarian failure 8 |  | Pathogenic | 30006057 | Heterozygous pathogenic variant |
|  |  | NM_001282717.2(STAG3):c.2445T>A (p.Ile815=) | Spermatogenic failure 61\|Premature ovarian failure 8\|not provided |  | Benign​ | VCV001230818 |  |
|  |  | NM_001282717.2(STAG3):c.2627G>A (p.Gly876Glu) | Premature ovarian failure 8 |  | Uncertain significance​ | VCV001214010 |  |
|  |  | NM_001282717.2(STAG3):c.2776C>T (p.Arg926Ter) | not provided\|Premature ovarian insufficiency\|Abnormality of the ovary\|Premature ovarian insufficiency\|Female infertility\|Premature ovarian failure 8 | | Likely pathogenic​​ | VCV000374000 |  |
|  |  | NM_001282717.2(STAG3):c.3381_3384del (p.Glu1128fs) | PRIMARY OVARIAN FAILURE 8 |  | Pathogenic | 34828315 |  |
| 74075 | Syce1* | NM_001143764.3(SYCE1):c.721C>T (p.Gln241Ter) | Premature ovarian failure 12 |  | Pathogenic | 25062452; 32917591 |  |
|  |  | NM_001143764.3(SYCE1):c.197-2A>G | Spermatogenic failure 15 |  | Pathogenic | 25899990 |  |
| 320558 | Sycp2 | NM_014258.4(SYCP2):c.3067_3071del (p.Lys1023fs) | Oligosynaptic infertility, Early spermatogenesis maturation arrest, non-obstructive azoospermia | Meiotic arrest at the pachytene spermatocyte stage | Pathogenic/Likely pathogenic​ | 31866047 | Heterozygous pathogenic variant |
|  |  | NM_014258.4(SYCP2):c.2793_2797del (p.Lys932fs) | Cryptozoospermia, Oligosynaptic infertility, non-obstructive azoospermia |  | Pathogenic/Likely pathogenic​ | 31866047 | Heterozygous pathogenic variant |
|  |  | NM_014258.4(SYCP2):c.2022_2025del (p.Lys674fs) | Cryptozoospermia, Oligosynaptic infertility, non-obstructive azoospermia |  | Pathogenic/Likely pathogenic​ | 31866047 | Heterozygous pathogenic variant |
| 20962 | Sycp3 | NM_001177949.2(SYCP3):c.657T>C (p.Thr219=) | PREGNANCY LOSS 4 |  | Pathogenic | 19110213 | Heterozygous pathogenic variant |
|  |  | NM_001177949.2(SYCP3):c.553-21_553-18del | PREGNANCY LOSS 4 |  | Pathogenic | 19110213 | Heterozygous pathogenic variant |
|  |  | NM_001177949.2(SYCP3):c.524_527del (p.Ile175fs) | Spermatogenic failure 4 |  | Uncertain significance​ | 29713536; 28801929 |  |
|  |  | NM_001177949.2(SYCP3):c.454-13_454-9del | Spermatogenic Failure |  | Benign​ | VCV000306763 |  |
|  |  | NM_001177949.2(SYCP3):c.435A>G (p.Glu145=) | Spermatogenic failure 4 |  | Benign​ | VCV000306764 |  |
|  |  | NM_001177949.2(SYCP3):c.241A>C (p.Ile81Leu) | Male infertility |  | Uncertain significance​ | VCV000869113 |  |
|  |  | NM_001177949.2(SYCP3):c.80T>C (p.Phe27Ser) | Spermatogenic failure 4 |  | Likely benign | VCV000880979 |  |
|  |  | NM_001177949.2(SYCP3):c.59A>G (p.Gln20Arg) | Spermatogenic failure 4 |  | Benign​ | VCV000880980 |  |
|  |  | NM_001177949.2(SYCP3):c.28A>T (p.Arg10Trp) | Spermatogenic failure 4 |  | Uncertain significance​ | VCV000880981 |  |
|  |  | NM_001177949.2(SYCP3):c.-53C>G | Spermatogenic failure 4 |  | Benign​ | VCV000880982 |  |
|  |  | NM_001177949.2(SYCP3):c.-64C>T | Spermatogenic failure 4 |  | Uncertain significance​ | VCV000306765 |  |
|  |  | NM_001177949.2(SYCP3):c.-74T>C | Spermatogenic failure 4 |  | Uncertain significance​ | VCV000882343 |  |
|  |  | NM_001177949.2(SYCP3):c.-106A>G | Spermatogenic failure 4 |  | Benign​ | VCV000882344 |  |
|  |  | NM_001177949.2(SYCP3):c.-122T>A | Spermatogenic failure 4 |  | Benign​ | VCV000306766 |  |
|  |  | SYCP3, 1-BP DEL, 643A | Spermatogenic failure 4 |  | Pathogenic | 14643120 |  |
| 74691 | Tdrd9 | NM_153046.3(TDRD9):c.46A>C (p.Ile16Leu) | Spermatogenic failure 30 |  | Uncertain significance​ | VCV001027806 |  |
|  |  | NM_153046.3(TDRD9):c.448G>A (p.Val150Met) | Spermatogenic failure 30 |  | Uncertain significance​ | VCV001027805 |  |
|  |  | NM_153046.3(TDRD9):c.720_723del (p.Ser241fs) | Azoospermia\|Spermatogenic failure 30 |  | Pathogenic | 28536242 |  |
|  |  | NM_153046.3(TDRD9):c.2106+2T>A | Spermatogenic failure 30 |  | Uncertain significance​ | VCV001301818 |  |
|  |  | NM_153046.3(TDRD9):c.3483_3484dup (p.Ser1162fs) | Azoospermia |  | Pathogenic | VCV001328950 |  |
| 320022 | Terb1 | NM_001136505.2(TERB1):c.1813C>T (p.Arg605Ter) | Spermatogenic failure 60 | Aberrant γH2AX pattern | Pathogenic | 32741963 | Double homozygous for both variants |
|  |  | NM_001136505.2(TERB1):c.289_290del (p.Leu97fs) | Spermatogenic failure 60 |  | Pathogenic | 32741963 |  |
|  |  | NM_001136505.2(TERB1):c.1703C>G (p.Ser568Ter) | Non-obstructive azoospermia\|Spermatogenic failure 60 | Arrest at spermatocyte stage | Pathogenic | 33211200 |  |
|  |  | NM_001136505.2(TERB1):c.733G>A (p.Gly245Arg) | Azoospermia |  | Pathogenic | VCV001328957 |  |
| 74401 | Terb2 | NM_152448.3(TERB2):c.434G>A (p.Ser145Asn) | Non-obstructive azoospermia |  | Likely pathogenic​​ | VCV001244236 |  |
|  |  | NM_152448.3(TERB2):c.457_458del (p.Thr153fs) | Spermatogenic failure 59 |  | Pathogenic | 33211200 |  |
|  |  | NM_152448.3(TERB2):c.544dup (p.Met182fs) | Spermatogenic failure 59 |  | Pathogenic | 33211200 |  |
| 83558 | Tex11 | NM_031276.3(TEX11):c.2568G>T (p.Trp856Cys) | Non-obstructive azoospermia |  | Pathogenic | 25970010 |  |
|  |  | NM_031276.3(TEX11):c.2047G>A (p.Ala683Thr) | Spermatogenic failure, X-linked, 2\|not specified | Partial meiotic arrest with very few postmeiotic cells detected. | Uncertain significance​ | 25970010 |  |
|  |  | NM_031276.3(TEX11):c.1751+2T>G | Non-obstructive azoospermia |  | Pathogenic | 25970010 |  |
|  |  | NM_031276.3(TEX11):c.1381-1G>A | Non-obstructive azoospermia |  | Pathogenic | 25970010 |  |
|  |  | NM_031276.3(TEX11):c.1208dup (p.Asn403fs) | Non-obstructive azoospermia |  | Pathogenic | 25970010 |  |
|  |  | NM_031276.3(TEX11):c.1006G>T (p.Glu336Ter) | Non-obstructive azoospermia |  | Pathogenic | 25970010 |  |
|  |  | NM_031276.3(TEX11):c.812del (p.Lys271fs) | Non-obstructive azoospermia |  | Pathogenic | 25970010 |  |
|  |  | NM_031276.3(TEX11):c.466A>G (p.Met156Val) | not specified\|Spermatogenic failure, X-linked, 2 | Meiotic arrest at spermatocyte stage | Uncertain significance​ | 25970010 |  |
|  |  | NM_031276.3(TEX11):c.405C>T (p.Ala135=) | Spermatogenic failure, X-linked, 2\|not provided |  | Benign​ | 25970010 |  |
|  |  | NM_031276.3(TEX11):c.253del (p.Val85fs) | Non-obstructive azoospermia |  | Pathogenic | 25970010 |  |
|  |  | NC_000023.10:g.69954448_70045530del | Spermatogenic failure, X-linked, 2 | Mixed testicular atrophy with meiotic arrest | Pathogenic | 25970010 |  |
| 104271 | Tex15 | NM_001350162.2(TEX15):c.9448C>T (p.Arg3150Ter) | Non-obstructive azoospermia |  | Uncertain significance​ | VCV001244246 |  |
|  |  | NM_001350162.2(TEX15):c.9223G>A (p.Gly3075Arg) | not provided\|non-obstructive azoospermia |  | Conflicting interpretations of pathogenicity | 31479588 |  |
|  |  | NM_001350162.1:c.9223G>A(;)7118G>A | Non-obstructive azoospermia |  | Uncertain significance​ | VCV000684732 |  |
|  |  | NM_001350162.2(TEX15):c.8197_8198del (p.Glu2733fs) | Non-obstructive azoospermia |  | Pathogenic | VCV001244247 |  |
|  |  | NM_001350162.2(TEX15):c.8083C>T (p.Arg2695Ter) | Spermatogenic failure 25 |  | Pathogenic | 28303806 |  |
|  |  | NM_001350162.2(TEX15):c.7777A>G (p.Thr2593Ala) | Non-obstructive azoospermia |  | Uncertain significance​ | VCV001244248 |  |
|  |  | NM_001350162.2(TEX15):c.7118G>A (p.Ser2373Asn) | Non-obstructive azoospermia |  | Likely benign | 31479588 |  |
|  |  | NM_001350162.2(TEX15):c.5170G>A (p.Ala1724Thr) | Non-obstructive azoospermia |  | Uncertain significance​ | VCV001285393 |  |
|  |  | NM_001350162.2(TEX15):c.4189del (p.Ser1397fs) | Spermatogenic failure 25 |  | Pathogenic | 28355598 |  |
|  |  | NM_001350162.2(TEX15):c.3568A>T (p.Lys1190Ter) | Spermatogenic failure 25 |  | Pathogenic | 28355598 |  |
|  |  | NM_001350162.2(TEX15):c.3323T>C (p.Leu1108Pro) | Spermatogenic failure 25 |  | Uncertain significance​ | VCV001030942 |  |
|  |  | NM_001350162.2(TEX15):c.3279T>G (p.Tyr1093Ter) | Spermatogenic failure 25\|Oligosynaptic infertility | Arrest at the primary spermatocyte stage | Pathogenic | 26199321 |  |
|  |  | NM_001350162.2(TEX15):c.1261G>A (p.Gly421Ser) | Spermatogenic failure 25 |  | Uncertain significance​ | VCV001339092 |  |
| 69716 | Trip13 | NM_004237.4(TRIP13):c.77A>G (p.His26Arg) | Oocyte maturation defect 9 |  | Pathogenic | 32473092 |  |
|  |  | NM_004237.4(TRIP13):c.518G>A (p.Arg173Gln) | Oocyte maturation defect 9 |  | Pathogenic | 32473092 |  |
|  |  | NM_004237.4(TRIP13):c.592A>G (p.Ile198Val) | Oocyte maturation defect 9 |  | Pathogenic | 32473092 |  |
|  |  | NM_004237.4(TRIP13):c.608+39T>G | not provided\|Mosaic variegated aneuploidy syndrome 3\|Oocyte maturation defect 9 | | Benign​ | VCV001241500 |  |
|  |  | NM_004237.4(TRIP13):c.673-1G>C | Mosaic variegated aneuploidy syndrome 3 |  | Pathogenic | 28553959 |  |
|  |  | NM_004237.4(TRIP13):c.712G>A (p.Asp238Asn) | Mosaic variegated aneuploidy syndrome 3 |  | Uncertain significance​ | VCV001031392 |  |
|  |  | NM_004237.4(TRIP13):c.739G>A (p.Val247Met) | Oocyte maturation defect 9 |  | Pathogenic | 32473092 |  |
|  |  | NM_004237.4(TRIP13):c.907G>A (p.Glu303Lys) | Oocyte maturation defect 9 |  | Pathogenic | 32473092 |  |
|  |  | NM_004237.4(TRIP13):c.1060C>T (p.Arg354Ter) | Mosaic variegated aneuploidy syndrome 3 |  | Pathogenic | 28553959 |  |

Gene variants in humans, the related conditions, and the clinical significance are extracted using Pubmed-clinvar; the phenotypes and others are summarized based on the associated publications. *Human phenotypes are recapitulated in mouse knockout models.
