# Supplementary material for: Genetic control of meiosis surveillance mechanisms in mammals
Source: Front Cell Dev Biol. 2023 Feb 23;11:1127440. doi: 10.3389/fcell.2023.1127440 (PMC9996228; doi:10.3389/fcell.2023.1127440)
Supplement: Supplementary file 4 [file DataSheet1.PDF]

Table S1 Meiotic prophase genes essential for mouse fertility.

**Gene ID**	**Symbol**	**Function in meiotic prophase I**	**Function category**	**Phenotypes in male mutants**		**Phenotypes in female mutants**		**PMID**	**Comments**
625662	**Ankrd31**	Regulates DSB formation by stabilizing REC114 and other factors on axes	Programmed DSB formation	Partial pachytene arrest, sterile	Defective synapsis, delayed early recombination process, failed CO formation between XY chromosomes	Reduced oocyte reserve, premature ovarian failure	Defective synapsis, delayed early recombination process	31003867; 31000436	
74068	Asz1	A structural role of nuage; regulates epigenetic and post-transcriptional silencing of retrotransposons by stabilizing MILI in nuage	Silencing of retrotranspos on	Complete arrest at zygotene-pachytene stage, sterile	Absence of sex body, loss of MILI	Normal fertility		19730684	
245000	Atr	Promotes the loading of strand-exchange proteins at DSBs and accumulation of recombination markers on the axes of unsynapsed chromosomes; required for correct timing of crossover formation and elongation of the synaptonemal complex	Meiotic recombination ; SC formation	Arrest at epithelial stage IV/mid-pachytene stage	Asynapsis , reduced RPA, RAD51 and DMC1 foci during early recombination, elevated RNF212 foci at pachynema	*N.A.*		29977027; 29976923	Germline-specific deletion
11920	Atm	DNA-damage checkpoint protein in response to SPO11-induced DSB, supressing DSB	Programmed DSB formation; DSB sensing	Complete arrest at early pachytene	Defective synapsis, abnormal assembly of ATR, DMC1 and RAD51, reduced γH2AX signals from	Complete arrest at dictyate stage, oocyte depletion by 4 dpp, infertile		9735362; 16055729; 15640358	

Table S1 Meiotic prophase genes essential for mouse fertility.

		formation via a negative feedback loop			leptotene to zygotene, rarely found sex body				
12189	Brca1	Majorly promotes the spreading of DNA damage response proteins and chromatin changes in meiotic silencing; has a minor role in meiotic recombination as regulating the timing of crossover formation	Meiotic silencing; meiotic recombination	Complete arrest after mid-pachytene, sterile	Decreased numbers of MSH4 foci and the delayed appearance of MLH1 foci, largely depleted H2AX signals at the X-centromeric end and its surrounding pericentric region	Normal fertility		24914237	Conditional, germline-specific deletion
12190	Brca2	Required for the recruitment of recombinases on DSBs in meiotic recombination	Meiotic recombination	Complete arrest at zygotene-pachytene. sterile	Lack of extensive synapsis, reduced number of RAD51 foci but abundant RPA foci	Some could progress through meiotic prophase I, with a high frequency of nuclear abnormalities, massive oocyte loss for 2-3 weeks postnatally, infertile		14660434	Homozygous mutant carrying a human BRCA2 gene
74666	Brme1	Stabilizes MEILB2; recruits recombinases via BRCA2-MEILB2-BRME1 complex during in meiotic recombination	Meiotic recombination	Arrest at pachytene with a small subset cell entering diplotene and undergoing apoptosis later, sterile	Disrupted DSB repair with decreased number of HSF2BP and DMC1/RAD51 foci, leading to failed synapsis and crossover recombination	Normal fertility		32463460; 32345962	
229776	Cdc14a	Dual-specificity phosphatase that counteracts CDK and is required for meiotic	Meiotic recombination initiation	Partial arrest at pachytene stage, subfertile	Decreased γH2ax signals in germ cells	Fertile		32679235	

Table S1 Meiotic prophase genes essential for mouse fertility.

		recombination initiation							
12566	Cdk2	A serine/threonine protein kinase that governs the structure of the nuclear envelope and the telomere-led chromosome movements; essential for homolog pairing, synapsis and DSB processing in males; promotes proper dynamics of SYCP3 in females	Chromosome movements; homolog pairing and synapsis; DSB processing	Complete arrest prior to mid-pachytene, sterile	Incomplete chromosome pairing, an extensive non-homologous synapsis unrepaired DSBs; in some of these spermatocytes, telomeres do not attach to the nuclear envelope, and sex chromosomes do not form a sex body	Arrest at dictyate stage, oocyte depletion by 14 dpp, infertile	Improper distribution of SYCP3	19494131; 12923533	
28135	Cep63	A centrosomal protein that might be required for chromosome dynamics in males	chromosome movements	Partial pachytene arrest, sterile	Aberrant DNA damage response triggered by centrosome-based mitotic errors. Impaired meiotic recombination, numerical and structural centrosome aberrations, chromosome entanglements and defective telomere clustering	Reduced follicles, fertile		26158450	
13164	**Dazl**	RNA-binding protein that mediates the translation of key proteins for the SC	SC formation; meiotic DSB repair	Arrest at early pachytene/pachytene-like stage, sterile	*N.A.*	Arrest at pachytene-diplotene stage, depletion of oocytes by 4dpp, infertile		31355046; 9288969; 14611631	Germ cell specific conditional knockout

Table S1 Meiotic prophase genes essential for mouse fertility.

		formation and DSB repair during spermatogenesis							
13404	**Dmc1**	Meiosis-specific recombinase that catalyses strand invasion into intact homologous duplexes	Meiotic recombination	Mostly arrest at zygotene/zygotene-like stage, sterile	Failed homolog pairing and synapsis, persistent RAD51 foci	Arrest at pachytene-like stage, most eliminated by 4 pp, nearly devoid of oocytes by 18 dpp, infertile		9660953; 9660954; 25636482	
54427	Dnmt3l	Mediates DNA methylation that influences molecular interactions important for chromatin compaction in spermatocytes	DNA methylation; chromatin modification	Complete arrest at early pachytene, sterile	Failed maternal methylation imprint establishment, aberrant chromatin packaging and failed homolog pairing and synapsis	Fertile		11934864; 15753313	
71241	Dmrt7	Mediates a transition in histone modifications that maintains transcriptional silencing of the sex chromosomes during prophase I in males	Meiotic silencing	Arrest at early-mid pachytene stage with a small proportion can progress to diplotene or beyond, sterile	Normal meiotic pairing and recombination and sex body formation, abnormal sex chromatin	Normal fertility		17098235; 17447844	The function is concluded due to the surviving diplotene cells, thus it's likely not linking to the arrest; or these transition defects elicit arrest or there might be other

Table S1 Meiotic prophase genes essential for mouse fertility.

									functions to detect or more.
94244	Fkbp6	Co-chaperone that is required to repress transposable elements and prevent their mobilization during spermatogenesis; acts via the piRNA metabolic process; controls homologous chromosomes pairing and synapsis specifically in males.	Silencing of retrotranspos on	Complete arrest at pachytene stage, sterile	Abnormal pairing and misalignments between homologous chromosomes, nonhomologous partner switches, and autosynapsis of X chromosome cores	Normal fertility		12764197	
60611	Foxj2	Transcriptional activator that regulates likely the DSB repair genes during meiotic recombination	Meiotic recombination	Complete arrest at pachytene stage, sterile	Failed chromosomal synapsis and DSB repair	*N.A.*		27316861	
15270	H2ax	Variant histone H2A that involves in DSB repair factors assembly, meiotic silencing, checkpoint signalling transducing and telomere clustering	Meiotic DSB repair, meiotic silencing; meiotic checkpoint; chromosome movements	Arrest at pachytene stage, with some could reach diplotene, sterile	Failed sex body formation and MSCI initiation, severe defects in meiotic pairing	Subfertile		11934988; 12689589; 14530383	
15201	Hells	Forms a pioneer complex with PRDM9 to open	Programmed DSB formation in males;	Arrest at early-mid pachytene, sterile.	Defective homologous chromosome	Lethal after birth	Incomplete chromosome synapsis	21349825; 17115026	

		chromatin at meiotic recombination hot spots in males; essential for the transcriptional repression of repetitive elements in females.	transcriptional repression in females		synapsis, lack of XY body		associated with persistent RAD51 foci and γH2AX phosphorylation, failure to load crossover-associated foci		
19183	**Hop2**	Required for proper homologous chromosome pairing and efficient DSB repair and cross-over during meiosis; stimulates both DMC1- and RAD51-mediated strand assimilation when forming heterodimer with Mnd1; may alone functions as a recombinase to promote formation of strand invasion by the NCO pathway, independently of DMC1 and RAD51	Meiotic recombination ; homolog paring	Complete arrest at pachytene-like stage, sterile	Defective synapsis and DSB repair with different extents	Absence of follicles in adult ovaries, infertile		14667414; 24304900	
67981	Hormad1	Axis component, promotes DSB formation; promotes SC formation independently of its	SC formation; meiotic DSB repair; meiotic checkpoint	Complete arrest at pachytene stage, sterile	Incomplete homolog pairing and synapsis, lack of XY body; disrupted meiotic recombination (less	Grossly normal ovarian development, arrest at blastocytes stage due to aneuploidy	Incomplete homolog pairing and synapsis, disrupted meiotic recombination (less DSB	21079677; 21478856	

Table S1 Meiotic prophase genes essential for mouse fertility.

		role in homology search; plays a key role in the male mid-pachytene checkpoint and the female meiotic prophase checkpoint			DSB formation, reduced number of RPA, RAD51 and DMC1 foci, dramatically reduced MLH1)	leading to infertility	formation, reduced number of RPA, RAD51 and DMC1 foci)		
75828	Hormad2	Promotes the accumulation of DDR components along the unsynapsed chromatin, but not at DNA DSBs or on DNA DSB-associated chromatin loops, thereby ensuring meiotic silencing; essential for meiotic checkpoint in response to asynapsis	Meiotic silencing and meiotic checkpoint	Complete arrest at mid-pachytene stage, sterile	Grossly normal homologous synapsis and meiotic recombination (DSB formation, early recombination processes), defective XY body, no presence of cells forming COs	Normal fertility		23039116; 22549958	
434438	Iho1	Required for DSB formation; probably acts by forming a complex with MEI4 and REC114, which activates DSBs formation, an essential step to ensure completion of synapsis	Programmed DSB formation; synapsis	Arrest at mid pachytene stage, sterile	Defective DSB formation and homologous synapsis	Nearly complete absence of oocytes in ovaries at 6-weeks, infertile	Defective homologous synapsis	27723721	
69260	Ing2	Regulates chromatin modification and	Chromatin modification	Arrest before/at pachytene stage, sterile	Incomplete recombination	Normal fertility		21124965	

		functionally interacts p53							
384619	**Kash5**	Bridges the nuclear envelope and telomeres through forming LINC complex with SUN1/2 proteins, thereby promoting chromosomal movements and homolog pairing	Chromosome movements; homolog pairing	Arrest at zygotene/pachytene stage, sterile	Defective pairing and synapsis, failed DSB repair	Depletion of follicles in adult ovaries, infertile		24062341; 24586178; 22826121	
110958	**M1ap**	Unknown function, required for meiosis I progression during spermatogenesis	Unknown	Partially at the zygotene/pachytene stage, while most cells advanced to metaphase I before arresting and entering apoptosis, sterile	Defective synapsis, DSB repair and CO formation	Normal fertility		23269666	
98558	Mael	A component of nuage, essential for transposon silencing in germ cells	Silencing of retrotransposon	Complete arrest at early pachytene stage, sterile	defective synapsis, persistent SPO11-independent DNA damage, delayed meiotic entry	3-fold reduction in the number of fetal oocytes at birth, increased aneuploidy	Defective homologous chromosome synapsis, DSB repair and CO formation	18694567; 24882376	
622554	Majin	Links telomere-nuclear envelope through forming TERB1/2-MAJIN complex; promotes meiotic chromosome	Chromosome movements; homolog pairing	Complete arrest at zygotene stage, sterile	Impaired homolog pairing and synapsis	Complete arrest at zygotene stage; depletion of follicles in adult ovaries, infertile	Impaired homolog pairing and synapsis	26548954; 30718482	

Table S1 Meiotic prophase genes essential for mouse fertility.

		movements and homologous pairing							
240697	Mcmdc2	Plays an important role in meiotic recombination; associated with the formation, or the stabilization, of DNA strand invasion events that promote homolog pairing and DSB repair	Meiotic recombination	Complete arrest at early pachytene stage, sterile.	Defective homolog pairing, synapsis and DSB repair, absent CO formation	Oocyte loss perinatally /soon after birth, complete devoid of oocytes in ovaries at 6-weeks, infertile	Defective homolog pairing, synapsis and DSB repair, absent CO formation	27760146; 27986806	
74369	Mei1	Required for normal meiotic chromosome synapsis; epistatic to *Dmc1,* likely involved in the meiotic DSB formation in spermatocytes	Programmed DSB formation; synapsis	Complete arrest at zygotene/pachytene stage, sterile	Defective homolog synapsis and DSB formation, intact recombinational DSB repair	Arrest at zygotene/pachytene stage, with a small number progressing to metaphase I with unpaired homologs and attempting the first meiotic division, reduced numbers of follicles, infertile	Defective homolog pairing and synapsis	11820814; 14668445; 15928951	
75033	Mei4	Required for DSB formation; probably acts by forming a complex with IHO1 and REC114, which activates DSB formation, an essential step to ensure completion of synapsis	Programmed DSB formation; synapsis	Complete arrest at mid/late pachytene stage, sterile	Defective DSB formation and homologous synapsis	Nearly complete of follicles in ovaries at 8-weeks, infertile		20551173; 25795304; 27723721	

Table S1 Meiotic prophase genes essential for mouse fertility.

74377	**Meilb2**	Binds and localizes BRCA2 to DSB sites; required for proper recombinase recruitment to DSB sites via BRCA2-MEILB2-BRME1 complex during in meiotic recombination	Meiotic recombination	Complete arrest at zygotene-pachytene. sterile	Abolished localization of RAD51 and DMC1 in spermatocytes	Partial meiotic prophase arrest, survived oocyte complete recombination with normal numbers of bivalent chromosomes, reduced oocyte reserve, subfertile	Impaired recombinase DMC1 and RAD51 recruitment, but milder than that in males	30760716	
75178	**Meiob**	Single-stranded DNA-binding protein that is required for homologous recombination and promotion of faithful and complete synapsis in meiosis I; forms complex with SPATA22, likely to ensure the stabilization of recombinases; displays single-stranded DNA 3'-5' exonuclease activity *in vitro*	Meiotic recombination ; synapsis	Complete arrest at zygotene-like stage, sterile	Defective homolog synapsis, DSB repair and CO formation	Complete oocyte loss by 2 dpp, infertile	Defective homolog synapsis, DSB repair and CO formation	24240703; 4068956	
76915	**Mnd1**	Required for proper homologous chromosome pairing and efficient DSB repair and cross-over during meiosis; stimulates both	Meiotic recombination ; homolog paring	Arrest at zygotene/pachy tene like stage, sterile	Defective synapsis and DSB repair with different extents	Absence of follicles in adult ovaries, infertile		24304900	

Table S1 Meiotic prophase genes essential for mouse fertility.

		DMC1- and RAD51-mediated strand assimilation when forming heterodimer with HOP2							
240069	Morc2b	Required for chromosomal synapsis and meiotic recombination in males and females	Programmed DSB formation; synapsis	Complete arrest at pachytene-like stage, sterile	Defective synapsis and meiotic recombination	Complete oocyte loss by 2 dpp, infertile	Defective synapsis and meiotic recombination	29329290	
83456	**Mov10l1**	ATP-dependent RNA helicase required during spermatogenesis to repress transposable elements and prevent their mobilization, which is essential for germline integrity	Silencing of retrotransposon	Complete arrest at zygotene-like stage, sterile	Defective synapsis, loss of DNA methylation and subsequent derepression of retrotransposons	Normal fertility		20534472; 20547853 23166510; 25762440	Conditional mutant
17535	Mre11	Component of the MRN complex, which governs meiotic DSB repair by facilitating DSB end resection and checkpoint signalling via activation of the ATM kinase during meiosis	Meiotic DSB repair; meiotic checkpoint	Temporal disturbance in meiotic progression, fertile	Defective synapsis and DSB repair	Temporal disturbance in meiotic progression, subfertile	Defective synapsis and DSB repair	14690604; 17291760	Hypomorphic mutant

Table S1 Meiotic prophase genes essential for mouse fertility.

55993	**Msh4**	DNA mismatch repair protein that locates at recombination nodules, facilitating homolog paring and crossover formation	Homolog paring; crossover formation	Complete arrest at zygotene stage, sterile	Failed homolog pairing and synapsis, incomplete meiotic recombination	Loss of the majority oocytes by 4 dpp, before dictyate stage, infertile	Failed homolog pairing and synapsis, incomplete meiotic recombination	10809667	
17687	**Msh5**	DNA mismatch repair protein that Locates at recombination nodules, facilitating homolog paring and crossover formation	Homolog paring; crossover formation	Complete arrest at zygotene stage, sterile	Failed homolog synapsis	Arrest at zygotene stage with some cells surviving to a post-pachytene stage, absence of follicles and oocytes in adult ovaries, infertile	Failed homologous synapsis	10072381	
17864	Mybl1	Transcription factor that promotes expression of piRNAs in male meiosis, which form complexes with Piwi proteins, mediating the repression of transposable elements	Silencing of retrotransposon	Arrest at epithelial Stage IV/mid-pachytene stage, sterile	Defective synapsis and DSB repair, lack of meiotic crossovers	Normal fertility	Normal progressions of synapsis, DSB repair and CO formation	21750041	
27354	Nbs1	Component of the MRN complex, which governs meiotic DSB repair by facilitating DSB end resection and checkpoint signalling via activation of the	Meiotic DSB repair; meiotic checkpoint	Complete arrest at zygotene stage, sterile	Defective synapsis and homologous recombination repair	Depletion of oocytes at diplotene stage, absence of oocytes in adult ovaries, infertile	Synapsis is completed in pachytene oocytes	31965061	Male-conditional germ-specific knockout; female-knockout mice rescued by BAC

Table S1 Meiotic prophase genes essential for mouse fertility.

		ATM kinase during meiosis							containing human NBS1 mutant
57746	Piwil2	Endoribonuclease that mediates the repression of transposable elements during meiosis by forming complexes composed of piRNAs and Piwi proteins and govern the methylation and subsequent repression of transposons	Silencing of retrotransposon	Complete arrest at zygotene/pachytene stage, sterile	*N.A.*	Normal fertility		14736746	
330890	Piwil4	Mediates the repression of transposable elements during meiosis by forming complexes composed of piRNAs and Piwi proteins and govern the methylation and subsequent repression of transposons	Silencing of retrotransposon	Complete arrest prior to pachytene stage, sterile	Defective synapsis and DSB repair	Normal fertility		17395546	
194908	Pld6	Presents endonuclease activity and plays a critical role in piRNA biogenesis, which	Silencing of retrotransposon	Complete arrest at zygotene/early pachytene stage, sterile.	Defective synapsis	Normal fertility		21397848; 21397847	

Table S1 Meiotic prophase genes essential for mouse fertility.

		mediates the repression of transposable elements during male meiosis							
213389	Prdm9	Histone methyltransferase that determines hotspot localization for DSB formation via binding specific DNA sequences through its zinc finger domains, thereby promoting meiotic recombination; during meiotic progression hotspot-bound PRDM9 interacts with several complexes, subsequently controlling the DSB repair pathway, pairing of homologous chromosomes and sex body formation	Meiotic recombination	Complete arrest at pachytene stage, sterile.	Severe impaired DSB repair, deficient pairing of homologous chromosomes and impaired sex body formation	Arrest at pachytene stage, massive oocyte loss perinatally, absence of follicles at 5-weeks, infertile	Severe impaired DSB repair and deficient pairing of homologous chromosomes	16292313; 27932493	
668929	Rad21l	Meiosis-specific component of some cohesin complex; required during the initial steps of prophase I in male	Component of cohesin complex; SC formation; synapsis initiation;	Complete arrest at zygotene-like stage, sterile	Defective homologous chromosome synapsis and DSB repair and absence of CO formation	Fertile with an age-dependent sterility/subfertile	Mild defects in homologous synapsis	21743440; 27172213	

Table S1 Meiotic prophase genes essential for mouse fertility.

		meiosis, including synaptonemal complex assembly, synapsis initiation and crossover recombination between homologous chromosomes; uniquely required for normal pericentromeric heterochromatin clustering events; not required for meiosis in females in young mice, while it is required later as mice age	meiotic recombination						
114714	Rad51c	Facilitates RAD51 formation, thereby promoting RAD51-mediated meiotic recombination; possibly plays a role in HJ resolution during late phase of meiotic recombination	Meiotic recombination	Incomplete arrest at pachytene stage, sterile	Defective synapsis and DSB repair on limited chromosomes, reduced CO formation	Infertile due to ovulation block	After superovulation, defects occur during metaphase I, leading to chromosomal abnormality	17312021	Hypomorphic mutation
3673	**Rec114**	Required for DSB formation; probably acts by forming a complex with IHO1 and MEI4, which activates DSBs formation, an	Programmed DSB formation; synapsis	Complete arrest at zygotene-like stage, sterile	Defective DSB formation and homologous synapsis	Complete arrest at zygotene-like stage, nearly complete absence of follicles in ovaries at 8-weeks, infertile	Defective DSB formation and homologous synapsis	30569039; 27723721	

Table S1 Meiotic prophase genes essential for mouse fertility.

		essential step to ensure completion of synapsis							
56739	**Rec8**	Key component of the meiotic cohesin complex that limits synapsis between homologous chromosomes; required for homologous recombination and the separation of homologous chromosomes	Component of cohesin complex; SC elongation; meiotic recombination ; separation of homologous chromosomes	Complete arrest at zygotene-like stage, sterile	Disrupted homologous chromosome synapsis, likely defective DSB repair and absence of CO formation.	Arrest at zygotene-like stage, absence of oocytes and follicles by 5dpp, infertile	Defective homolog synapsis	15935783	
108086	Rnf216	E3 ubiquitin ligase that regulates PKA stability during meiosis	Ubiquitination	Incomplete arrest at zygotene stage, sterile	*N.A.*	Normal fertility		33724554	
100155	**shoc1**	ATPase required for the formation of crossover recombination intermediates in meiotic prophase I in male and female germ cells	Meiotic recombination	Complete arrest at zygotene-like stage, sterile	Incomplete synapsis, failed meiotic recombination with persistent recombination intermediates and absence of crossing over	Rapid oocyte loss since E17.5-1dpp, complete depletion of oocytes by 6dpp, infertile	Incomplete synapsis, failed meiotic recombination with persistent recombination intermediates and absence of COs	30272023	
75801	**Six6os1**	Central element component of the synaptonemal complex	Element of SC	Complete arrest at pachytene-like stage, sterile	Failed synapsis between homologs, most of which are properly paired. Persistent DSBs, RAD51/DMC1 foci and MSH4, absence of MLH1.	Complete arrest at pachytene-like stage, oocyte depletion by 6 dpp, infertile	Failed synapsis between homologs, most of which are properly paired, persistent DSBs, RAD51/DMC1 foci	27796301	

Table S1 Meiotic prophase genes essential for mouse fertility.

							and MSH4, absence of MLH1		
21402	Skp1	Ubiquitin E3 ligase that plays crucial and distinct roles in meiotic DSB homeostasis, synapsis initiation and maintenance and sister chromatid cohesion during meiotic prophase I	Meiotic DSB homeostasis; synapsis initiation and maintenance; sister chromatid cohesion	Complete arrest at zygotene-like stage	Defective synapsis and early meiotic recombination	Arrest at pachytene-like stage, loss of the majority oocytes at 1dpp and complete loss by 6 weeks	Defective synapsis, less defective DSB repair compared to males	35489071	Conditional inactivation in germ cells prior to meiosis
73333	Slc25a31	ADP: ATP antiporter that mediates the translocation of ADP and ATP across the inner mitochondrial membrane; supports DSB repair and synapsis progression that require sufficient ATP supplies during meiotic prophase I	Meiotic DSB repair; synapsis	Partially arrest at pachytene stage, sterile	Defective synapsis and DSB repair in partial pachytene cells, the other cells likely be eliminated due to the deficiency of ATP supplies from mitochondria.	Normal fertility		17681941; 19556438	
140557	**Smc1b**	Meiosis-specific component of cohesin complex; required for the maintenance of meiotic cohesion and only to a minor extent, for its establishment; required for stable telomere attachment to the	Component of cohesin complex; synapsis; meiotic recombination; chromosome movements	Arrest at early-mid pachytene stage, sterile	Incomplete synapsis (shortened SC, discontinuous SC and unsynapsed AEs), defective DSB repair on asynapsed chromosomes, absence of COs.	Progress to dictyate stage but exhibit massive aneuploidy during the meiotic divisions, absence of follicles in 5-7 months old ovaries	Incomplete synapsis/reduced SC length and reduced CO numbers	15146193; 18180366; 19841137;16258540	

Table S1 Meiotic prophase genes essential for mouse fertility.

		nuclear envelope; contributes to AE formation and the organization of chromatin loops along the AE							
380709	Spata22	Meiosis-specific protein that forms complex with MEIOB, likely to ensure the stabilization of recombinases during meiotic recombination	Meiotic recombination	Complete arrest at zygotene-like stage/before mid-pachytene stage/epithelial stage IV, sterile	Impaired homolog synapsis and DSB repair.	Almost complete absence of oocytes by 10 dpp, infertile	Impaired homolog synapsis and DSB repair	22011390; 24240703	
70891	Spdya	Regulates telomere-NE attachment and NE structure by interacting with CDK2 and SUN1; critical for homolog pairing and synapsis	Homolog pairing and synapsis	Complete arrest epithelial stage IV, mostly before mid pachytene stage, sterile	Defective homolog synapsis with extensive non-homologous pairing and telomere fusions, impaired late-recombination stages, and absence of COs.	Arrest at pachytene/pachytene-like stage, absence of follicle in adult ovaries, infertile	Defective homolog synapsis with extensive non-homologous pairing and telomere fusions, reduced CO formation	27025256; 34039995	
26972	**Spo11**	Component of a component of TOPOVIL complex, with TOPOVIBL; mediates DNA cleavage that forms the DSB together with TOPOVIBL; Essential for the phosphorylation of SMC3, HORMAD1 and HORMAD2	Programmed DSB formation	Complete arrest at zygotene-like/early-mid pachytene stage, sterile	No synapsis or little synapsis between non-homologous chromosomes, meiotic recombination doesn't initiate.	Survive to diplotene stage with reduced numbers of oocyte, complete depletion of oocytes in 2-3 months old ovaries, infertile	No synapsis or little synapsis between non-homologous chromosomes, meiotic recombination doesn't initiate	11106739; 11106738; 15640358; 16055729	

Table S1 Meiotic prophase genes essential for mouse fertility.

50878	**Stag3**	Meiosis specific component of cohesin complex; required for AE formation and the stability of meiosis-specific cohesins	Component of cohesin complex; SC formation	Complete arrest at zygotene-like stage without reaching pachytene/stage IV, sterile	Disrupted AE formation and homologous chromosome synapsis, failed DSB repair.	Complete depletion of oocytes by 7 dpp, infertile	Disrupted AE formation, homologous synapsis, failed DSB repair	24597867; 24608227; 24992337; 27172213; 24797475	Common phenotypes summarized; phenotypes observed in these studies differ somewhat depending on the knockout allele of Stag3
77053	Sun1	Bridges the nuclear envelope and telomeres through forming LINC complex with KASH5 proteins, thereby promoting chromosomal movements and homolog pairing	Chromosome movements; homolog pairing	Complete arrest at pachytene-like stage, sterile	Disrupted telomere NE Attachment, impaired homologous chromosome pairing, synapsis, and recombination	Complete arrest at pachytene-like stage, oocyte depletion by 5 dpp, infertile	Disrupted telomere NE Attachment, impaired homologous chromosome pairing and synapsis	17543860	
74075	**Syce1**	Major component of the transverse central element of SC; essential for synapsis initiation; have roles in the the assembly and stabilization of SC and the stabilization of homolog	Element of SC	Arrest at epithelial stage IV, sterile	Absent SC and sex body, aligned homologs at variable distances, incomplete DSB repair, absence of COs	Complete depletion of follicles in adult ovaries, infertile	Absent SC and sex body, aligned homologs at variable distances, incomplete DSB repair, absence of COs	19247432	

Table S1 Meiotic prophase genes essential for mouse fertility.

		interactions during meiotic recombination							
71846	Syce2	Major component of the transverse central element of SC; required for propagating synapsis along the paired chromosome axes	Element of SC	Arrest at epithelial stage IV, sterile	Failed SC formation between aligned homologs with small regions of synapsis, absence of sex body, incomplete DSB processing, absence of COs	Minute adult ovaries, infertile.	Failed SC formation between aligned homologs with small regions of synapsis, incomplete DSB processing and absence of COs	17339376	
75459	Syce3	Major component of the transverse central element of SC; required for the loading of other central element proteins, and for initiating synapsis between homologous chromosomes	Element of SC	Arrest at epithelial stage IV, sterile	Failed synapsis between mostly aligned AEs, persistent DSB, incomplete DSB repair and absence of COs	Complete depletion of follicles in adult ovaries, infertile	Affected homolog pairing and failed synapsis between homologs, persistent DSB, incomplete DSB repair and absence of COs	21637789	
20957	Sycp1	Major component of the transverse filaments of synaptonemal complexes. Essential for synapsis initiation and required for SC assembly and normal centromere pairing.	Element of SC	The majority arrest at pachytene stage with a proportion reaches diplotene or, exceptionally metaphase I, sterile	Absent SC between aligned homologs, persistent DNA damage and absence of COs and sex body	Absence of growing follicles and oocyte, infertile		5937223	
320558	**Sycp2**	Major component of the axial/lateral	Element of SC	Complete arrest at	Failed AE formation and the subsequent	Subfertile	Interrupted homolog synapsis	16717126	

Table S1 Meiotic prophase genes essential for mouse fertility.

		elements of SC; required for the SC assembly; may be involved in the organization of chromatin		zygotene/zygotene-like stage, sterile	disruption of homolog synapsis		but exhibiting SYCP1 localization, nuclear aggregates of SYCP3		
20962	**Sycp3**	Structural component of the axial/lateral element of the SC; required for centromere pairing in males and for chiasmata formation and for the structural integrity of meiotic chromosomes in females; required for efficient phosphorylation of HORMAD1 and HORMAD2 during meiotic prophase I	Element of SC	Complete arrest at zygotene, sterile	Failed homolog pairing, AE, and SC formation, affected distribution of RPA/RAD51	Subfertile, severely reduced oocyte pool, exhibits a sharp reduction in litter size that increases with advancing maternal age due to aneuploid oocytes	Disrupted homolog synapsis but exhibiting SYCP1 localization, defective meiotic chromosome segregation	10678170; 12004129	
74691	**Tdrd9**	TP-binding RNA helicase that acts functionally with MIWI2 via the piRNA metabolic process, thereby repressing transposable elements and preventing their mobilization	Silencing of retrotransposon	Complete arrest at zygotene stage, sterile	Failed homolog synapsis and incomplete meiotic recombination	Normal fertility		20059948	

Table S1 Meiotic prophase genes essential for mouse fertility.

320022	**Terb1**	Links telomere-nuclear envelope through forming TERB1/2-MAJIN complex; promotes meiotic chromosome movements and homolog pairing	chromosome movements; homolog pairing	Complete arrest at zygotene-like stage, sterile	Defective homolog pairing and synapsis, impaired DSB repair and absent CO formation, disrupted telomere NE Attachment	Complete arrest at zygotene-like stage; depletion of follicles in adult ovaries, infertile	Defective homolog pairing and synapsis	24413433; 30718482	
74401	**Terb2**	Links telomere-nuclear envelope through forming TERB1/2-MAJIN complex; promotes meiotic chromosome movements and homolog pairing	chromosome movements; homolog pairing	Complete arrest at zygotene stage, sterile	Impaired homolog pairing and synapsis	Complete arrest at zygotene stage; depletion of follicles in adult ovaries, infertile	Impaired homolog pairing and synapsis	26548954; 30718482	
17771	Tesmin	Translocates into the nuclei around the zygotene-pachytene transition and promotes meiosis progression beyond the mid-pachytene stage during spermatogenesis.	Meiosis progression	Complete arrest at early pachytene stage, sterile	Roughly normal meiotic recombination progression and minor defects in synapsis	Fertile		34388164; 31916570	
83558	**Tex11**	Involves in initiation and/or maintenance of chromosome synapsis and formation of crossovers during meiosis	Synapsis; crossover formation	Partially arrest at pachytene stage, the remaining cells arrest at anaphase I, sterile	Asynapsis in partial spermatocytes and reduced COs	Fertile with reduced litter size	Asynapsis in partial oocytes and reduced COs	18316482	

Table S1 Meiotic prophase genes essential for mouse fertility.

66654	Tex12	Major component of the transverse central element of SC; Required for propagating synapsis along the paired chromosome axes	Element of SC	Complete arrest at epithelial stage IV, sterile	Failed elongation of synapsis between homologs, normal loading of early recombination markers with absence of sex body	Complete depletion of follicles by 7 dpp, infertile	Failed elongation of synapsis between homologs, impaired DSB processing and absence of COs	18611960	
104271	**Tex15**	Required for normal chromosomal synapsis and the formation of DMC1 and RAD51 foci on meiotic chromosomes; essential executor of PIWIL4-piRNA pathway directed transposon DNA methylation and silencing in males	Meiotic recombination ; synapsis; silencing of retrotranspos on	Complete arrest at early pachytene stage, sterile	Failed homolog synapsis and meiotic recombination with the lack of RAD51/DMC1	Normal fertility		18283110; 32381626; 32719317	
73679	Tex19.1	Promotes SPO11-dependent meiotic recombination with UBR2 in males and maintain sister chromatid cohesion postnatally in females; participates in the repression of retrotransposable elements and preventing their mobilization in males	Meiotic recombination ; sister chromatid cohesion; silencing of retrotranspos on	Heterogenous phenotypes, from partial to complete meiotic arrest at pachytene stage, varied fertility from subfertile to infertile	Defective early meiotic recombination, failed homology synapsis	Subfertile	Unaffected early recombination and homolog synapsis, premature sister chromatid separation and homologue missegregation during meiosis I	18802469; 21103378; 28708824; 32232464	

Table S1 Meiotic prophase genes essential for mouse fertility.

381196	Top6bl	Component of a topoisomerase 6 complex with SPO11; together with SPO11, mediates DSC formation in meiotic recombination	Meiotic recombination ; homolog synapsis	Complete arrest before or at mid-pachytene stage, sterile	Defective homologous synapsis and little synapsis between non-homologous chromosomes; failed recombination initiation	Largely depletion of primordial and primary follicles at 30dpp		26917764	
69716	**Trip13**	Promotes early steps upstream of the assembly of RAD51 complexes in meiotic recombination that leads to non-crossovers pathways; needed for efficient completion of homologous synapsis and SC formation; required for development of higher-order chromosome structures; required for depletion of HORMAD1 and HORMAD2 from synapsed chromosomes	Meiotic recombination ; synapsis	Complete ($Trip13^{sev/sev}$) or incomplete ($Trip13^{mod/mod}$) arrest at pachytene stage/ epithelial stage IV, sterile	Defective synapsis between autosomal homologs (only displayed in $Trip13^{sev/sev}$), failed XY body formation and DSB repair, reduced COs (only displayed in $Trip13^{sev/sev}$)	Depletion of follicles by 21dpp in $Trip13^{sev/sev}$; by 2 months in $Trip13^{mod/mod}$, infertile	Defective synapsis between autosomal homologs and reduced COs (only displayed in $Trip13^{sev/sev}$) failed DSB repair	17696610; 20711356	
224826	Ubr2	E3 ubiquitin-protein ligase that plays a critical role in chromatin	Meiotic recombination ; synapsis; silencing of	Incomplete arrest at pachytene stage, sterile	Defective during early DSB repair, disrupted homolog	Surviving females from embryonic lethality are fertile		14585983; 22616001; 28708824	Variable defects in spermatog enesis are

Table S1 Meiotic prophase genes essential for mouse fertility.

		inactivation and chromosome-wide transcriptional silencing during meiosis via ubiquitination of histone H2A; promotes SPO11-dependent recombination foci to accumulate and drive robust homologous chromosome synapsis with Tex19.1; involves in inhibiting LINE-1 retrotransposon mobilization	retrotransposon; meiotic silencing		pairing and synapsis				identified in mice with other strain background PMID 20080676
381678	Zcwpw1	Dual histone methylation reader specific for PRDM9-catalyzed histone marks (H3K4me3 and H3K36me3) that facilitates the repair of PRDM9-induced meiotic DSB in males	Meiotic DSB repair	Complete arrest at zygotene/pachytene stage, sterile	Complete failed homology synapsis, accompanied by incomplete DSB repair and lack of COs	Normal fertility until mid-adulthood	Completion of meiosis with delayed meiotic prophase I	31453335; 32374261; 2352380; 32744506	
22697	Zscan21	Strong transcriptional activator that plays an important role in the progression of meiotic prophase I in spermatocytes	Transcriptional activator		Failed homolog synapsis, impaired DSB with decreased RAD51 foci, reduced COs	*N.A.*		27492080	Spermatocyte-specific gene knockout

Table S1 Meiotic prophase genes essential for mouse fertility.
